# Identification of CD84 as a potent survival factor in acute myeloid leukemia

**DOI:** 10.1172/JCI176818

**Published:** 2025-04-08

**Authors:** Yinghui Zhu, Mariam Murtadha, Miaomiao Liu, Enrico Caserta, Ottavio Napolitano, Le Xuan Truong Nguyen, Huafeng Wang, Milad Moloudizargari, Lokesh Nigam, Theophilus Tandoh, Xuemei Wang, Alex Pozhitkov, Rui Su, Xiangjie Lin, Marc Denisse Estepa, Raju Pillai, Joo Song, James F. Sanchez, Yu-Hsuan Fu, Lianjun Zhang, Man Li, Bin Zhang, Ling Li, Ya-Huei Kuo, Steven Rosen, Guido Marcucci, John C. Williams, Flavia Pichiorri

**Affiliations:** 1 State Key Laboratory of Cardiovascular Diseases and Medical Innovation Center, Shanghai East Hospital, School of Life Sciences and Technology, Frontier Science Center for Stem Cell Research, Tongji University, Shanghai, China.; 2 Judy and Bernard Briskin Center for Multiple Myeloma Research, Department of Hematology and Hematopoietic Cell Transplantation, and; 3 Department of Hematologic Malignancies Translational Science, Beckman Research Institute, City of Hope, Duarte, California, USA.; 4 Department of Hematology, the First Affiliated Hospital, Zhejiang University School of Medicine, Zhejiang Provincial Key Laboratory of Hematopoietic Malignancy, Zhejiang University, Hangzhou, Zhejiang, China.; 5 Department of Systems Biology, Beckman Research Institute,; 6 Department of Pathology, and; 7 Department of Cancer Biology and Molecular Medicine, Beckman Research Institute, City of Hope, Duarte, California, USA.

**Keywords:** Cell biology, Hematology, Oncology, Antigen, Bone marrow, Cancer immunotherapy

## Abstract

Acute myeloid leukemia (AML) is an aggressive and often deadly malignancy associated with proliferative immature myeloid blasts. Here, we identified CD84 as a critical survival regulator in AML. High levels of CD84 expression provided a survival advantage to leukemia cells, whereas CD84 downregulation disrupted their proliferation, clonogenicity, and engraftment capabilities in both human cell lines and patient-derived xenograft cells. Critically, loss of CD84 also markedly blocked leukemia engraftment and clonogenicity in MLL-AF9 and inv(16) AML mouse models, highlighting its pivotal role as a survival factor across species. Mechanistically, CD84 regulated leukemia cells’ energy metabolism and mitochondrial dynamics. Depletion of CD84 altered mitochondrial ultrastructure and function of leukemia cells, and it caused downmodulation of both oxidative phosphorylation and fatty acid oxidation pathways. CD84 knockdown induced a block of Akt phosphorylation and downmodulation of nuclear factor erythroid 2-related factor 2 (NRF2), impairing AML antioxidant defense. Conversely, CD84 overexpression stabilized NRF2 and promoted its transcriptional activation, thereby supporting redox homeostasis and mitochondrial function in AML. Collectively, our findings indicate that AML cells depend on CD84 to support antioxidant prosurvival pathways, highlighting a therapeutic vulnerability of leukemia cells.

## Abstract 2

CD84 is a key survival protein for leukemia cells and a potential target to treat AML.

## Introduction

Acute myeloid leukemia (AML) is a malignancy that rapidly progresses and presents with uncontrollable accumulation of immature myeloid blasts in the BM and peripheral blood (PB). Chemotherapy has been used to date as a standard treatment for AML (1), and recently the development of novel therapeutic interventions, including the introduction of the BCL2 inhibitor venetoclax, have positively impacted overall response rates in AML, especially among younger patients (2). However, while the 5-year survival rate for young patients is around 30%, for elderly patients, it is as low as 5%–10% (3), indicating that further understanding of the disease biology is needed. Although AML is a very heterogeneous disease characterized by cancer cell clones carrying different molecular and cytogenetic abnormalities (4), it has been abundantly shown that deregulation in the cellular redox networks associated with high ROS levels are common features for AML cells (5). Several published data have shown that an increase in redox state correlates with mutational events supporting oncogene activation, tumor suppressor gene downmodulation, increased aerobic metabolism, and mitochondrial dysfunction (6). AML cells can survive high ROS levels by compensating with molecular mechanisms that upregulate active antioxidant systems to avoid excessive ROS accumulation and protect leukemic cells from oxidative stress–induced cell death (7, 8). Hence, downregulation of the antioxidative pathways renders AML cells more vulnerable compared with normal cells (9).

In AML cells, nuclear factor erythroid 2-related factor 2 (NRF2) plays a pivotal role in regulating oxidative stress pathways by transcriptionally activating antioxidant genes, essentially protecting the cancer cells from damage caused by ROS and contributing to their resistance to chemotherapy drugs (10, 11). Previously published data have shown that NRF2 activation, protein stability, and nuclear translocation strongly depend on the PI3K/AKT pathway, which is a survival pathway frequently activated in AML patient blasts (12). When NRF2 translocates into the nucleus, it binds to the antioxidant response element–dependent (ARE-dependent) cytoprotective genes (13). This process leads to overexpression of several genes including antioxidant genes, antiapoptotic genes, and detoxifying genes (13), which contribute to cancer cell growth and therapeutic resistance (12, 14, 15).

CD84 (SLAM5) is a member of the signaling lymphocyte activation molecule (SLAM) family and is expressed at different levels in the normal hematopoietic lineage (16, 17). Biochemical and structural studies indicate that CD84 forms homophilic dimers by self-association (18) and in doing so, enhances IFN-γ secretion (19) and induces programmed cell death ligand 1 (PD-L1) upregulation on chronic lymphocytic leukemia (CLL) cells, resulting in suppression of T cell activation against cancer cells (20). Moreover, it has been found that CD84 is an essential survival factor for CLL, acting by activating a signaling cascade that involves CD84 tyrosine phosphorylation, EAT-2 recruitment, and increased AKT phosphorylation, resulting in BCL2 upregulation (21); importantly, this survival cascade also plays a pivotal role in AML pathophysiology (22). Recently, we (17) and others (17, 23) have identified CD84 to be a robust myeloid-derived suppressor cell (MDSCs) surface marker, but its role in myeloid malignancies has yet to be investigated.

Here, we report that CD84 is a highly expressed tumor-associated target in AML. We also report that CD84 suppression strongly limits AML cell growth and extends the survival of AML xenografted mice. Finally, we observed that CD84 knockdown induces the downregulation of antioxidant genes involved in glutathione (GSH) metabolism through AKT phosphorylation and NRF2 downmodulation. Our findings suggest that CD84 is a critical survival factor regulating metabolic processes in leukemia cells, highlighting its role as a potential therapeutic target for AML.

## Results

### CD84 is overexpressed in AML cells.

**Figure 1:**
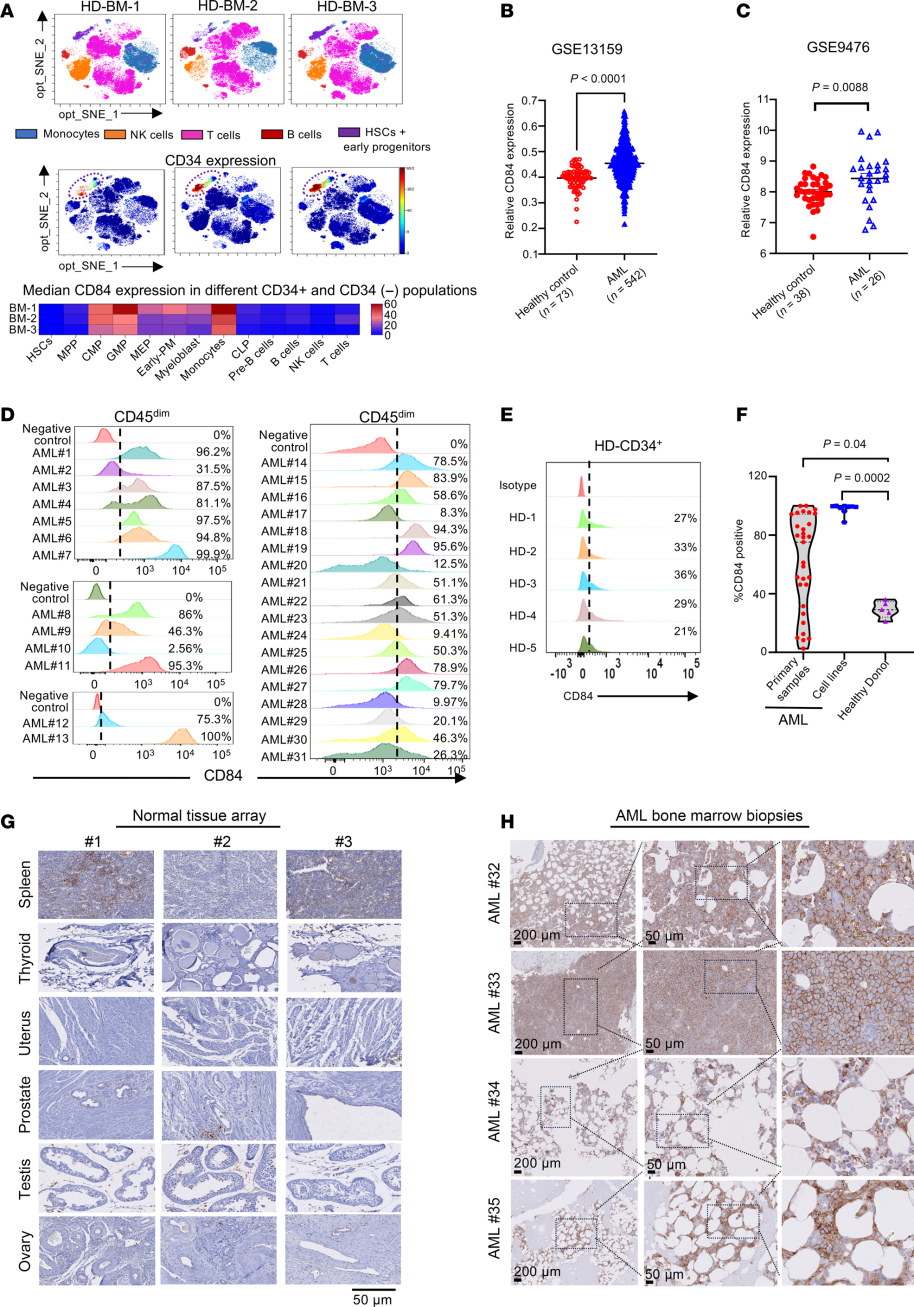
CD84 is overexpressed in AML. (**A**) BM cells were subjected to CyTOF immunophenotyping comprising 39 surface markers tailored to detect different immune subsets. Analysis was performed with Cytobank platform in independent healthy donors (*n* = 3). (**B**) Scatter plots of CD84 mRNA expression in BM-MNCs from patients with AML (*n* = 542) and healthy donors (*n* = 73) (from GEO GSE13159 dataset) indicating increased CD84 expression in AML specimens. Graphs are presented as mean ± SEM. Statistical significance was assessed by 2-tailed unpaired *t* test. (**C**) Scatter plots of CD84 mRNA expression in leukemia blasts from patients with AML (*n* = 26) and CD34^+^ cells isolated from healthy donors (*n* = 38) (from GEO GSE9476 dataset) indicating increased CD84 expression in AML specimens. Graphs are presented as mean ± SEM. Statistical significance was assessed by 2-tailed unpaired *t* test. (**D**) Histogram showing CD84 surface protein expression in different AML patient specimens (*n* = 31) as analyzed by flow cytometry, highlighting that CD84 is highly expressed in AML primary patient cells. PE anti-human CD84 (clone CD84.1.21; BioLegend) was used (1 μl/test). (**E**) Histogram showing flow cytometry profiles of CD84 expression in healthy donors from the CD34^+^ cellular population. The analysis was conducted in independent donors (*n* = 5). (**F**) Violin plot shown the percentage of CD84-expressing cells among AML primary patients (*n* = 31), AML cell lines (*n* = 9), and healthy donor cells (*n* = 5). Data are represented as mean ± SEM. Statistical significance was assessed by 1-way ANOVA. (**G**) Representative images of immunohistochemical staining of CD84 performed in normal tissue array. Each normal tissue stained for CD84 was obtained from a minimum of 3 independent normal donors. Scale bars: 50 μm. (**H**) Representative images of immunohistochemical staining of CD84 in AML BM. Original magnification, ×200. Scale bars: 200 μm. The analysis was conducted in 15 independent AML donor biopsies (see also Supplemental Figure 2G).

To comprehensively characterize the expression pattern of CD84 in the hematopoietic system, we first examined its mRNA expression across various hematopoietic lineages using the BloodSpot database (NCBI’s Gene Expression Omnibus [GEO] GSE42519) (Supplemental Figure 1A; supplemental material available online with this article; https://doi.org/10.1172/JCI176818DS1), and we also performed single-cell mass cytometry analysis of BM cells isolated from healthy donors (*n* = 3) (Figure 1A and Supplemental Table 1). The analysis revealed that CD84 is almost completely absent in normal hematopoietic stem cells (HSCs) and hematopoietic multipotent progenitors (MPPs). Conversely, CD84 levels are markedly upregulated in common myeloid progenitors (CMPs), granulocyte-monocyte progenitors (GMPs), early promyelocytes (early-PMs), and monocytes. Aligned with recently published data (24), significantly lower CD84 expression was observed in common lymphoid progenitors (CLPs) and the mature lymphoid lineage (Figure 1A and Supplemental Figure 1A). These findings indicate that CD84 is predominantly expressed in early myeloid progenitors and their immediate derivatives, suggesting a potential role for CD84 in myeloid lineage early commitment. Because AML is characterized by the accumulation of clonal myeloid progenitors (25), we decided to investigate CD84 expression in this setting. Based on the gene-expression profiling datasets that include large cohorts of patients with AML (GEO GSE13159 and GSE9476), we observed that BM mononuclear cells (BM-MNCs) of AML specimens showed statistically significant increases in *CD84* mRNA levels compared with those of normal healthy counterparts (Figure 1, B and C). By using the DepMap portal (https://depmap.org/portal/depmap/) (26), we analyzed *CD84* mRNA levels across the entire spectrum of human cancer cell lines (*n* =1,197) and found a distinct elevated expression of CD84 in leukemia, especially in AML cell lines (*n* = 44), which we also confirmed by flow cytometry analysis (*n* = 9) (Supplemental Figure 1, B and C). Consistently in AML cell lines we found statistically significant correlations between mRNA and protein expression (*R* = 0.85, *P* = 0.01, *n* = 6) (Supplemental Figure 1D). GEPIA (http://gepia.cancer-pku.cn) presented AML as the predominant expresser of *CD84* (Supplemental Figure 1E). Importantly, according to PRECOG database analysis (https://precog.stanford.edu/) (27) and KMPlot database analysis (https://kmplot.com/analysis/) (28), elevated *CD84* mRNA expression is associated with shorter overall survival in patients with AML (GEO GSE10358, *P* = 0.01; KMPlot database, *P* = 0.028) (Supplemental Figure 1, F and G) but this was not observed when The Cancer Genomic Atlas (TCGA) and Beat AML (https://www.cancer.gov/ccg/blog/2019/beataml) genomic data sets were interrogated (29). We analyzed the correlation between CD84 expression and AML subtypes based on either molecular classification, French-American-British (FAB) subtypes, or mutational status. According to GSE13159 dataset (GEO), the relative expression of *CD84* over control was greater in karyotypes like inv(16), t(11q23)/mixed-lineage leukemia (MLL) and normal karyotype (*P* < 0.0001), compared with t(15;17) and complex karyotype (*P* < 0.05), but absence of upregulation was found in t(8;21) (Supplemental Figure 2A). AML subtypes with different mutations show comparable expressions (Supplemental Figure 2, B and C). FAB subtype analysis revealed statistically significant lower *CD84* expression in M3 compared with other subtypes (*P* < 0.001), with comparable levels across non-M3 subtypes (Supplemental Figure 2D). These findings reinforce the relevance of CD84 in different AML subtypes. Flow cytometry analysis confirmed that CD84 is upregulated in primary AML samples obtained from different sources (*n* = 31) (Figure 1D) and AML cell lines (*n* = 9) (Supplemental Figure 1B) compared with levels in healthy donor CD34^+^ cells (*n* = 5) (Figure 1, E and F), independent of disease status or cytogenetic abnormalities (Supplemental Table 2). Notably, high-surface CD84 positivity (> 70%) was found in more than 50% of the AML samples we analyzed (Supplemental Table 3). Conversely to AML cell lines, in primary AML samples obtained from different sources, we did not find a direct correlation between mRNA and protein expression (*R^2^* = 0.003, *P* = 0.8, *n* = 15), suggesting that the heterogenicity of the primary sample population, which is different between cohorts, may affect this analysis.

Aligned with this observation, a mass cytometry (CyTOF) panel was constructed to further investigate CD84 expression across the cellular composition of AML samples (Supplemental Table 1). Using FlowSOM (30) analysis, we observed CD84 to be predominantly within AML blast populations, but that variable levels of noncancer immune subsets were still detectable in AML primary samples (Supplemental Figure 2E). To further establish CD84 as a potential selective target in leukemogenesis, we employed a tissue array assay to examine endogenous expression of CD84 in normal tissue as well as AML BM. IHC analysis showed that, in normal tissue, CD84 positivity was exclusively detected in the spleen (SP), a major lymphoid organ (Figure 1G); this observation is in agreement with the reported presence of variable expression of CD84 in the hematopoietic lineage (16), as is also confirmed by flow analysis in normal immune subsets isolated from healthy donors (Supplemental Figure 2F). Importantly, a strong CD84 signal was detected in almost 100% of the blasts present in the BM biopsies obtained from patients with relapsing AML (*n* = 15) carrying different genetic abnormalities (Figure 1H and Supplemental Figure 2G).

### CD84 downregulation impairs AML cell survival.

**Figure 2:**
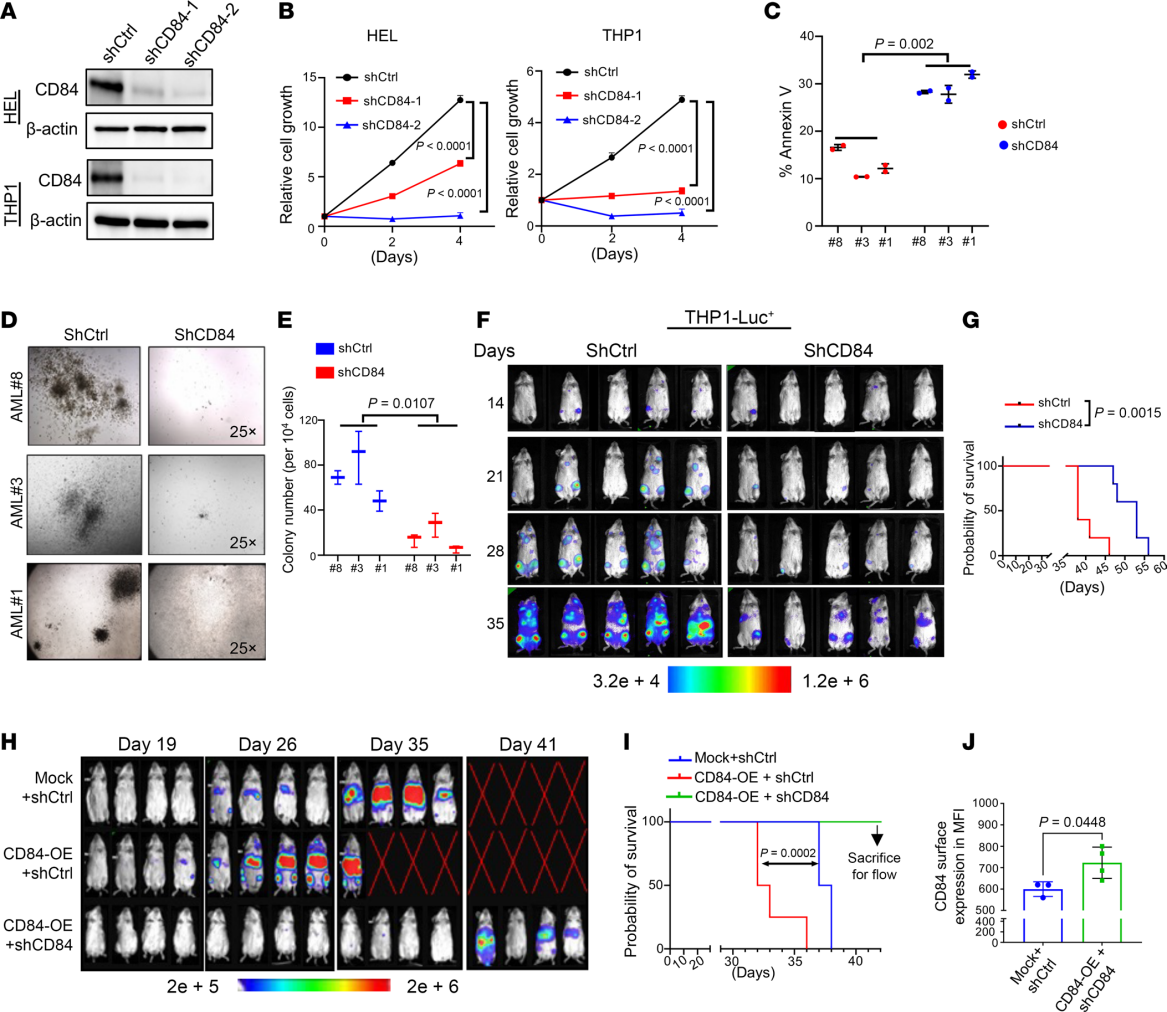
CD84 deletion dampens AML survival in both AML cell lines and cell-derived xenograft. (**A**) Western blot of the indicated proteins in THP1 cells and HEL cells transduced with 2 shRNAs against CD84 (shCD84-1; shCD84-2) or scramble control (shCtrl). Data are representative of at least 2 independent experiments. (**B**) Cell proliferative analysis of THP1 cells and HEL cells transduced with shCtrl or shCD84 lentiviral vectors. (**C**) Bar chart showing apoptosis levels indicated by annexin-APC/DAPI in 3 AML patient specimens transduced with shCtrl or shCD84 lentiviral vector. (**D** and E) AML cells obtained from 3 different donors (AML #1, #3, #8) were transduced with shCtrl or shCD84 lentivirus. Representative colony images are in **D**. The graph in **E** shows AML colony-formation cell (CFC) frequencies after 10 days of culture. *n* = 3 independent replicates per sample. (**F**) Bioluminescent imaging showing the tumor burden in xenograft NSG mice on days 14–35 following shCtrl- or shCD84-transduced THP1-luciferase cell transplantation (*n* = 5 per group). (**G**) Kaplan-Meier analysis of survival of THP1-luciferase cell–transplanted (shCtrl or shCD84) NSG mice. Each group consisted of 5 mice. (**H**) Bioluminescent imaging showing the tumor burden in xenograft NSG mice on days 19–41 following mock/shCtrl-, CD84-OE/shCtrl–, or CD84-OE/shCD84–transduced THP1-luciferase cell transplantation (*n* = 4 per group). (**I**) Kaplan-Meier analysis of survival of THP1-luciferase cell–transplanted (mock/shCtrl, CD84-OE/shCtrl, or CD84-OE/shCD84) NSG mice (*n* = 4 per group). (**J**) Bar chart showing the CD84 surface expression in BM cells from NSG mice xenografted with THP1 luciferase cells transduced with mock/shCtrl or CD84-OE/shCD84. Data are represented as mean ± SEM and are representative of 3 biological replicates (**B** and **E**) and 3 independent experiments (**C**). Each dot in **J** represents 1 mouse. Statistical significance was assessed by 2-way ANOVA (**B**, **D**, and **E**); 2-way ANOVA (mix model; **C**); log-rank test (**G** and **I**); and 2-tailed unpaired t test (**J**).

**Figure 3:**
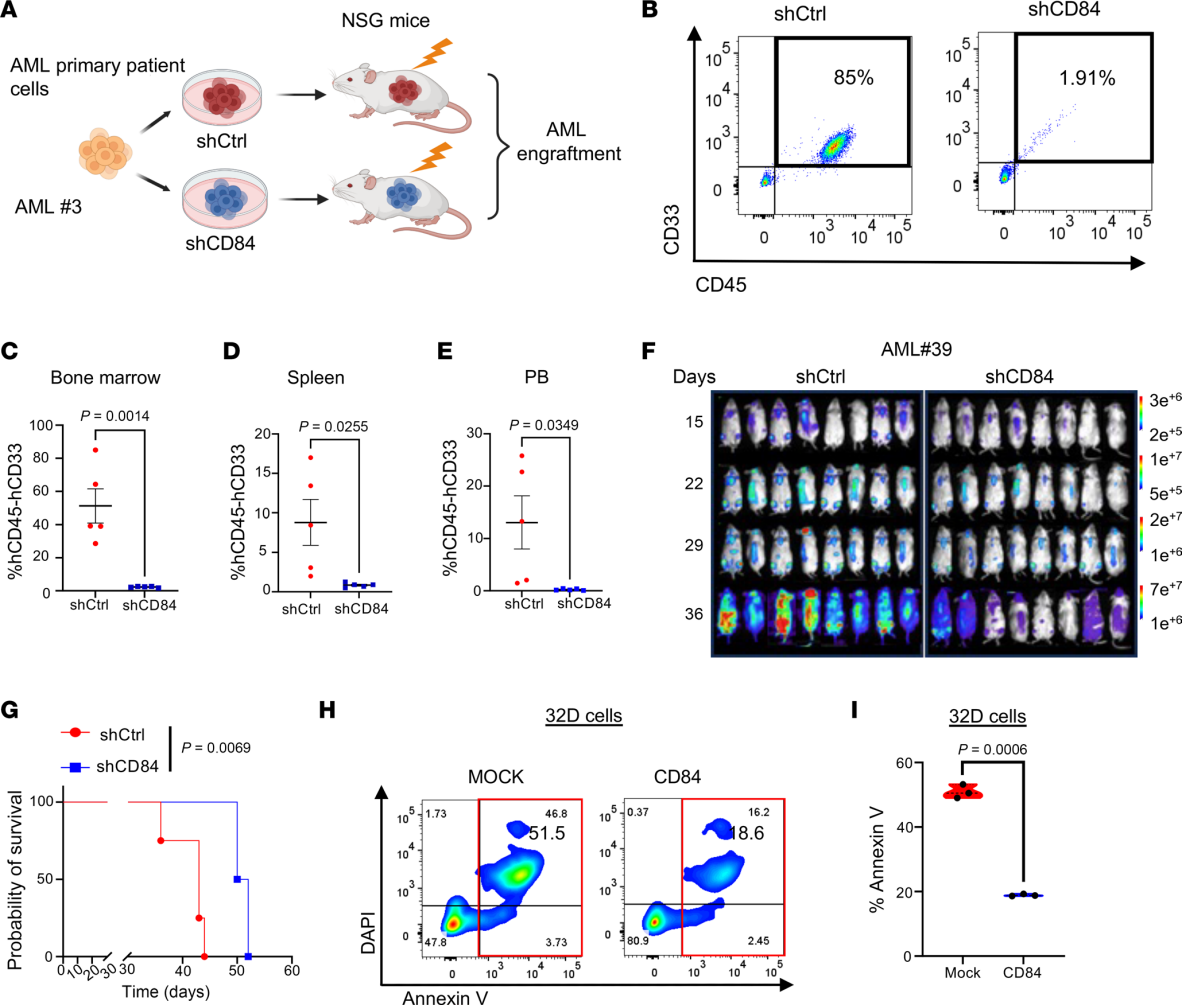
CD84 loss impairs AML development in PDX. (**A**) Schematic of the design and procedures of generating a CD84 knockdown, AML PDX model. AML primary patient cells were transduced with shCtrl or shCD84 lentivirus. After puromycin selection, shCtrl or shCD84 AML primary cells were injected into irradiated NSG mice. (**B**) Representative flow cytometry profile of human AML cells (human CD45^+^/CD33^+^) engrafted in BM. (**C**–**E**) Scatter plots showing the percentage of human AML cells (human CD45^+^/CD33^+^) engrafted in BM (**C**), SP (**D**), and PB (**E**) of recipient NSG mice (*n* = 5 per group). Data are represented as mean ± SEM and are representative of 5 individual mice per group. Statistical significance was assessed by 2-tailed unpaired *t* test. (**F**) Bioluminescent imaging showing the tumor burden in xenograft NSG mice (frontal and dorsal) following shCtrl- or shCD84-transduced AML PDX-luciferase cell transplantation (*n* = 4 per group). (**G**) Kaplan-Meier survival analysis of AML PDX-luciferase cell–transplanted (shCtrl or shCD84) NSG mice (*n* = 4 per group). Statistical significance was assessed by log-rank test. (**H**) Flow cytometry profile showing apoptosis levels indicated by annexin-APC/DAPI in 32D cells transfected with lentivirus including CD823-mock vector or CD823-CD84 WT. (**I**) Violin plot showing apoptosis levels indicated by annexin V-APC/DAPI in 32D cells transduced with mock or CD84 WT. Data are represented as mean ± SEM and are representative of 3 biological replicates. Statistical significance was assessed by 2-tailed unpaired *t* test.

To investigate the role of CD84 in AML, we conducted both gain- and loss-of-function studies. We used the lentiviral vector–based shRNA system to knock down the expression of CD84 (shCD84-1 and shCD84-2) (Figure 2A) and demonstrated that CD84 downregulation caused a statistically significant inhibition of cell growth (Figure 2B) as well as induction of apoptosis in AML cell lines (Supplemental Figure 3, A and B). While CD84 knockdown did not affect the clonogenic activity of healthy donor–derived CD34^+^ cells (Supplemental Figure 3C), in AML primary patient cells, we found that its downregulation substantially induced cell apoptosis (Figure 2C and Supplemental Figure 3, D and E) and inhibited cell colony formation (Figure 2, D and E). To understand whether CD84 knockdown could also affect the ability of AML cells to engraft, we downregulated CD84 in luciferase-expressing THP1 cells and transplanted the cells into immunodeficient NSG mice. Attenuated tumor burden (Figure 2F) as well as prolonged survival (Figure 2G) were observed in recipients of these cells, relative to the control (*P* = 0.0015). To further assess the importance of CD84 in regulating AML cell engraftment capabilities, we ectopically overexpressed CD84 (CD84-OE) in THP1-luciferase cells (Supplemental Figure 3, F and G). Our in vivo data show that mice engrafted with THP1 CD84-OE cells had a statistically significant reduction in survival, compared with the control group that was transduced with an empty viral vector (mock) (Figure 2, H and I, *P* = 0.002), supporting that CD84 provided a further survival advantage in these cells. Notably, the early mortality observed in the CD84-OE group was completely abolished when CD84 overexpression in THP1 cells was knocked down by shRNA (CD84 OE+shCD84) (Supplemental Figure 3, F and G, and Figure 2, H and I). At the time of relapse (~41 days) mice engrafted with CD84 OE+shCD84 were euthanized to assess CD84 expression in the AML cells. Notably, CD84 OE+shCD84 mice carried THP1 cells that at relapse not only lost CD84 silencing, but maintained statistically significant CD84 upregulation compared with the mock/shCtrl engrafted mice (*P* = 0.045) (Figure 2J and Supplemental Figure 3H), further supporting that CD84 overexpression facilitates AML progression, this effect being specifically mitigated by CD84 deletion. To further investigate the role of CD84 in AML patient–derived cells, we transplanted AML primary patient cells transduced with shCD84 or shCtrl plasmid into NSG mice (Figure 3A and Supplemental Figure 3I). We observed that the AML burden was statistically significantly lower in the BM (Figure 3, B and C; *P* = 0.001, 51.2% versus 2.3%), SP (Figure 3D; *P* = 0.026, 8.79% versus 0.84%), and PB (Figure 3E; *P* = 0.035,13.06% vs 0.24%) in the CD84 knockdown group compared with levels in the control recipient animals. We also found reduced SP weight in CD84-knockdown mice (Supplemental Figure 3, J and K) at the time the mice were sacrificed (*P* = 0.0416; 0.064 g versus 0.032 g). Moreover, we knocked down CD84 in a luciferase-expressing AML patient-derived xenograft (PDX) cells and transplanted them into immunodeficient NSG mice (Supplemental Figure 3L). Consistently, mice receiving CD84-knockdown cells exhibited reduced tumor burden (Figure 3F) and extended survival (Figure 3G) compared with control animals. We further validated in this experiment that the relapse observed in the shCD84 group might be attributed to escape from shRNA knockdown (Supplemental Figure 3M). To assess the function of CD84 in AML cell maintenance, we employed a murine IL-3–dependent myeloid cell line, 32D, for functional analysis, as endogenous CD84 is undetectable in this line. We ectopically overexpressed WT CD84 in 32D cells, or empty vector (EV) (mock) as control. In the absence of murine IL-3, there was a more than 60% decrease in apoptosis in CD84-expressing 32D cells induced by IL-3 deprivation relative to mock cells (*P* = 0.0006) (Figure 3, H and I, and Supplemental Figure 3N). Our findings indicate that CD84 is required for a distinct AML phenotype, including proliferation, clonogenicity, and leukemic engraftment.

### CD84 is essential for leukemia cell maintenance in AML mouse models.

**Figure 4:**
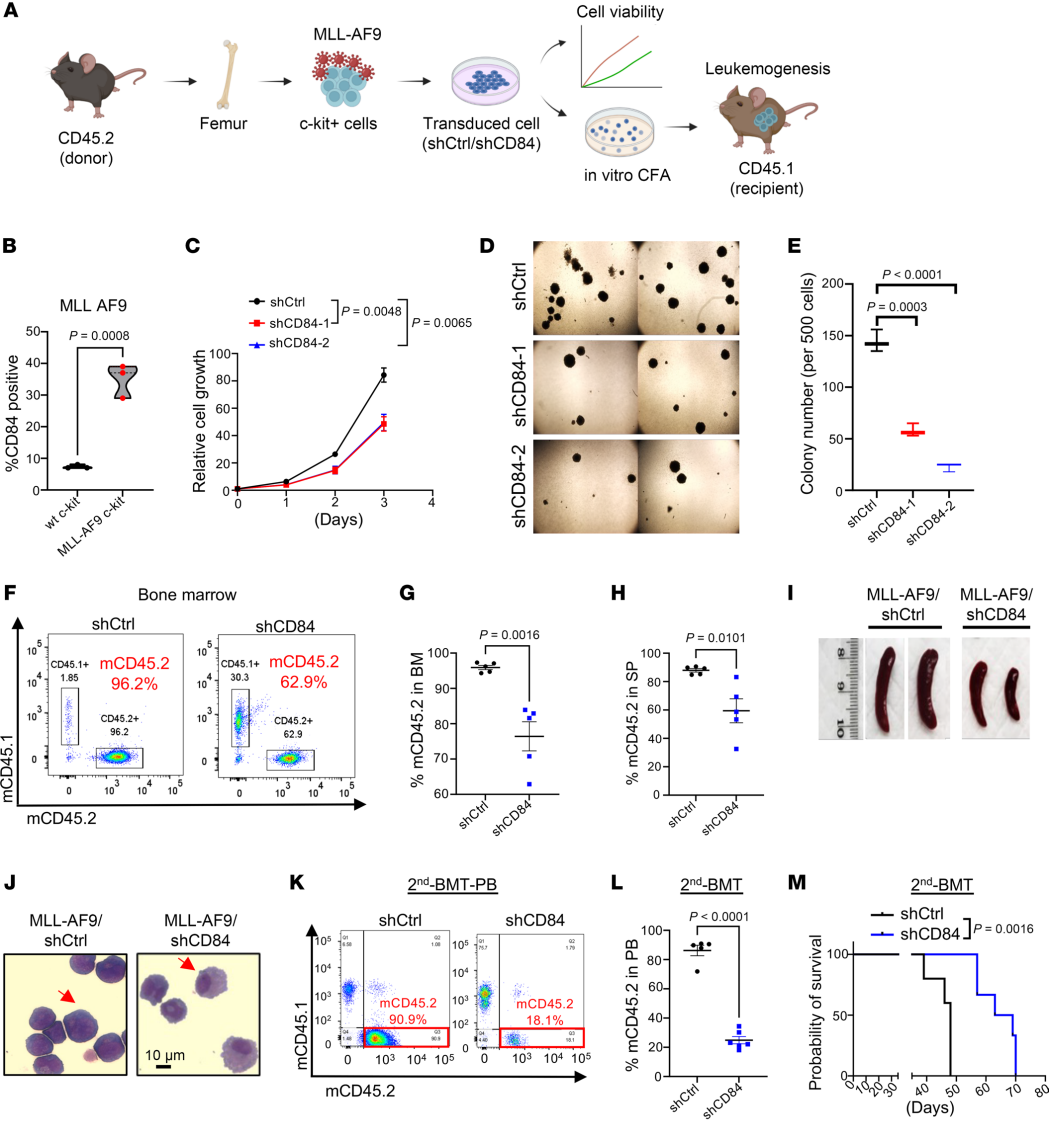
CD84 is essential for AML maintenance in vivo. (**A**) Design and procedures of generating an MLL-AF9 AML mouse model with CD84 knockdown. (**B**) Violin chart showing CD84 expression in c-kit^+^ cells before and after MLL-AF9 transduction. (**C**) Connecting line graph representing cell-proliferative analysis of MLL-AF9 AML cells transduced with shCtrl or shCD84 (shCD84-1; shCD84-2) lentiviral vector. (**D**) Representative colony formation images of MLL-AF9 c-kit^+^ cells transduced with shCtrl or shCD84 (shCD84-1; shCD84-2). Images were acquired in tiles by the City of Hope microscopy core facility using ZEN 3.1 (blue edition, Carl Zeiss Microscopy GmbH). Original maginifcation, ×10. (**E**) The graph shows MLL-AF9 AML colony formation cell numbers after 7 days of culture. (**F**) Representative scatter plots showing the percentage of donor cells (mouse CD45.2) transduced with shCtrl or shCD84 and engrafted in the BM. (**G** and **H**) Graphs showing the percentages of mouse CD45.2 in the BM (**G**) and SP (**H**) of recipient mice (mouse CD45.1) at around 5 weeks after BM transplantation (*n* = 5 per group). (**I**) Representative SP image of recipient mice xenografted with shCtrl-MLL-AF9 or shCD84-MLL-AF9. (**J**) Representative images of Wright-Giemsa staining of BM from recipient mice transplanted with shCtrl-MLL-AF9 or shCD84-MLL-AF9 AML cells (red arrows indicate AML blast). (**K** and **L**) Representative scatter plot (**K**) and associated graph (**L**) showing the leukemic engraftment in the PB of recipient mice (CD45.1) xenografted with MLL-AF9 AML with or without CD84 silencing (CD45.2) upon secondary BM transplantation on day 38. (**M**) Kaplan-Meier analysis of survival of secondary BM-transplanted mice with MLL-AF9 cells (shCtrl or shCD84) (*n* = 5 per group). Data are represented as ± SEM and are representative of 3 independent experiments (**B**, **C**, and **E**), 5 individual mice (**G** and **H**); and 5 individual mice per group (**L**). Statistical significance was assessed by 2-tailed unpaired t test (**B**, **G**, **H**, and **L**); 2-way ANOVA (mixed model; **C**); 1-way ANOVA (**E**); and log-rank test (**M**).

**Figure 5:**
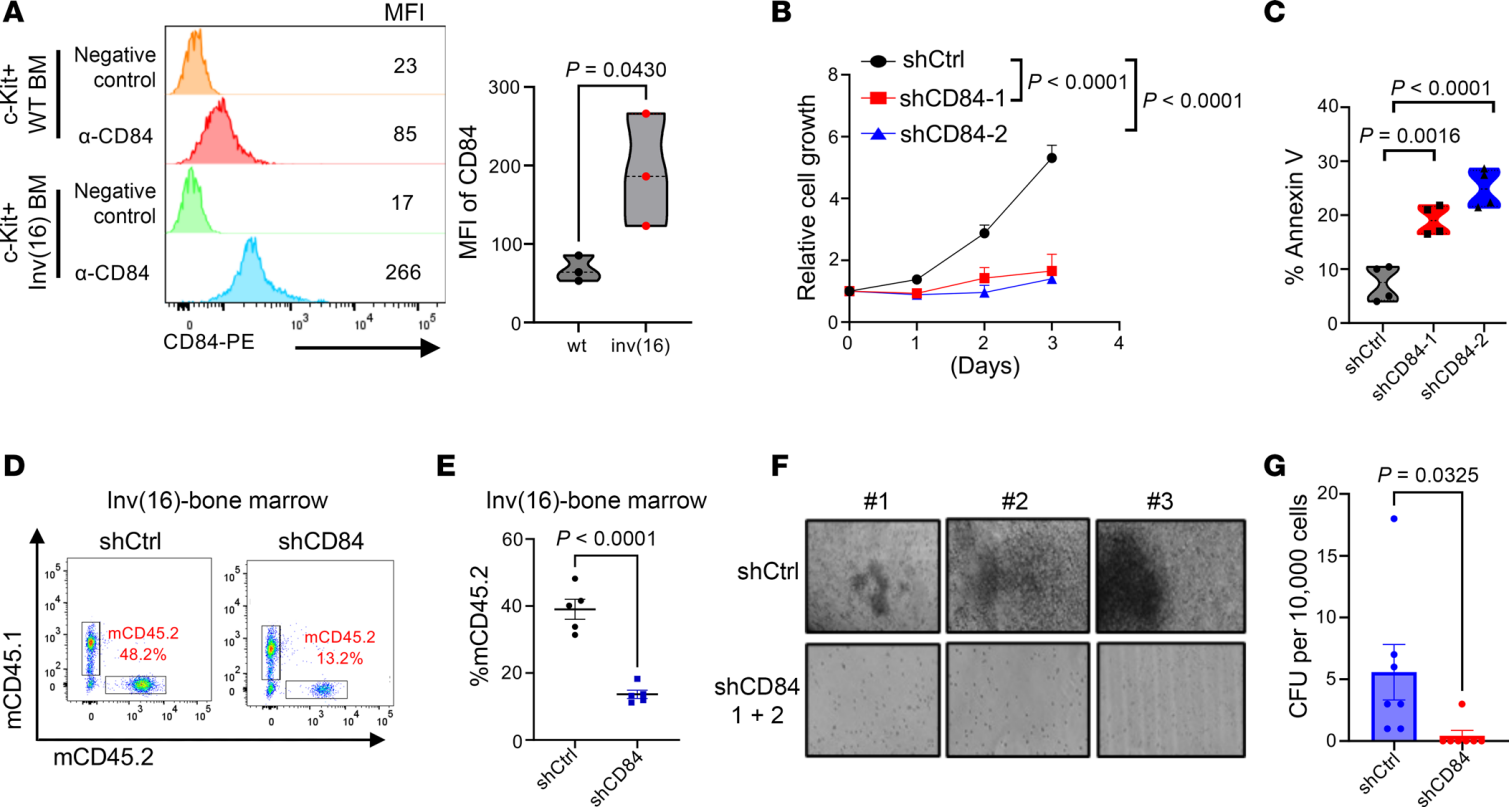
CD84 is essential for AML maintenance in inv(16) mouse model. (**A**) Histogram and violin chart showing CD84 expression in inv(16) c-kit^+^ cells relative to WT c-kit^+^ cells. Data are represented as mean ± SEM and are representative of 3 independent experiments and mice. Statistical significance was assessed by 2-tailed unpaired *t* test. (**B**) Connecting line graph representing cell proliferative analysis of inv(16)-AML cells transduced with shCtrl or shCD84 (shCD84-1; shCD84-2) lentiviral vector. Data are represented as mean ± SEM and are representative of 3 independent experiments. Statistical significance was assessed with 2-way ANOVA (mixed model). (**C**) Violin plot showing apoptosis levels indicated by annexin-APC/DAPI in inv(16)-AML cells transduced with shCtrl or shCD84 lentiviral vector. Data are represented as mean ± SEM and are representative of 4 independent experiments. Statistical significance was assessed by 1-way ANOVA. (**D**) Representative flow cytometry profile of donor cells (mouse CD45.2) engrafted in BM from shCtrl-inv(16) or shCD84-inv(16) transplanted mice. (**E**) Scatter plot showing the leukemic engraftment in the BM of recipient mice (CD45.1) xenografted with inv(16) AML with or without CD84 silencing (CD45.2) (*n* = 5 per group). Data are represented as mean ± SEM and are representative of 5 individual mice. Statistical significance was assessed by 2-tailed unpaired *t* test. (**F**) Representative colony images of inv(16)-AML cells transduced with shCtrl or shCD84-1+2. Original magnificiation, 10×. (**G**) The bar graph shows colony formation numbers of inv(16) mice transduced with shCtrl or shCD84-1+2 after 7 days of culture. Data are represented as mean ± SEM and are representative of 7 independent replicates. Statistical significance was assessed by 2-tailed unpaired *t* test.

To understand the functional role of CD84 in leukemogenesis, we generated murine MLL-AF9-HSPC pre-LSCs by transducing an HSC-enriched hematopoietic progenitor cell population (c-kit^+^) with a lentivirus encoding the *MLL-AF9* fusion oncogene (Figure 4A). We observed a statistically significant upregulation of CD84 expression (Figure 4B and Supplemental Figure 4A) and enhanced colony formation (Supplemental Figure 4B) in MLL-AF9–transduced c-kit^+^ cells compared with WT c-kit cells. We knocked down mouse CD84 using 2 independent shRNAs (mouse shCD84-1 and mouse shCD84-2) in MLL-AF9 cells and confirmed efficient knockdown at mRNA and protein levels (Supplemental Figure 4, C–E). As shown in Figure 4C, CD84 knockdown inhibited cell growth. As expected, CD84 depletion dampened the clonogenic potential of MLL-AF9 AML cells (Figure 4, D and E) and induced apoptosis (Supplemental Figure 4F). To evaluate the role of CD84 in leukemogenesis in vivo, we conducted mouse BM transplantation assays in irradiated C57BL/6 (CD45.1) syngeneic recipient mice (Figure 4A). We found that CD84 knockdown reduced leukemic engraftment in BM (Figure 4, F and G), SP (Figure 4H), and PB (Supplemental Figure 4G), along with reduced splenomegaly (Figure 4I and Supplemental Figure 4H), compared with levels in recipients without CD84 silencing. Notably, CD84 knockdown also reduced the immature blast cell population (Figure 4J). In secondary BM transplantation, leukemic engraftment was further attenuated in the CD84-knockdown group, resulting in a statistically significant increase in the median survival (66 days) compared with that of the control group animals (median survival 48 days; *P* = 0.0016) (Figure 4, K–M). Because our data have shown that AML cells transduced only with 1 CD84 silencing sequence can overcome shCD84 in vivo, to enhance the efficiency of CD84 knockdown, and to conduct longer term in vivo studies, we transfected MLL-AF9 cells with both shCD84-1 and shCD84-2 targeting CD84. CD84 expression was abrogated when AML cells were treated with the double-CD84 knockdown (Supplemental Figure 4I). Correspondingly, the colony formation assay revealed a complete absence of colony formation in the CD84-knockdown group (Supplemental Figure 4, J and K). In addition, we employed a second mouse AML model harboring inv(16) (p13q22), which creates the fusion gene *CBFB-MYH11* (CM). We transduced CD84 shRNA and control shRNA into leukemic BM cells collected from primary AML mice bearing CM/inv(16) AML (31). Consistent with the observations in MLL-AF9 AML studies, CD84 was upregulated in inv(16) leukemic (c-kit^+^) cells (Figure 5A). CD84 knockdown substantially arrested inv(16) AML cell growth and increased apoptosis (Supplemental Figure 4L, and Figure 5, B and C). CD84 deficiency also disrupted the leukemogenic potential of inv(16) AML cells, decreasing by more than 80% the leukemic engraftment in the BM (*P* < 0.0001), SP (*P* = 0.004), and PB (*P* = 0.014) of recipients (Figure 5, D and E, and Supplemental Figure 4M), leading to a substantial reduction in SP weight (Supplemental Figure 4, N and O), compared with mice carrying leukemia cells with intact CD84 expression. Consistently, when we transfected inv(16) AML cells with both shCD84-1 and shCD84-2 to target CD84, no colonies were formed in the CD84-knockdown group (Figure 5, F and G, and Supplemental Figure 4P). Collectively, these data demonstrate that CD84 plays a critical role for AML maintenance in vivo and its role as survival factor in AML cells is conserved across models.

### CD84 knockdown deactivated energy metabolism and induced mitochondrial stress in AML.

**Figure 6:**
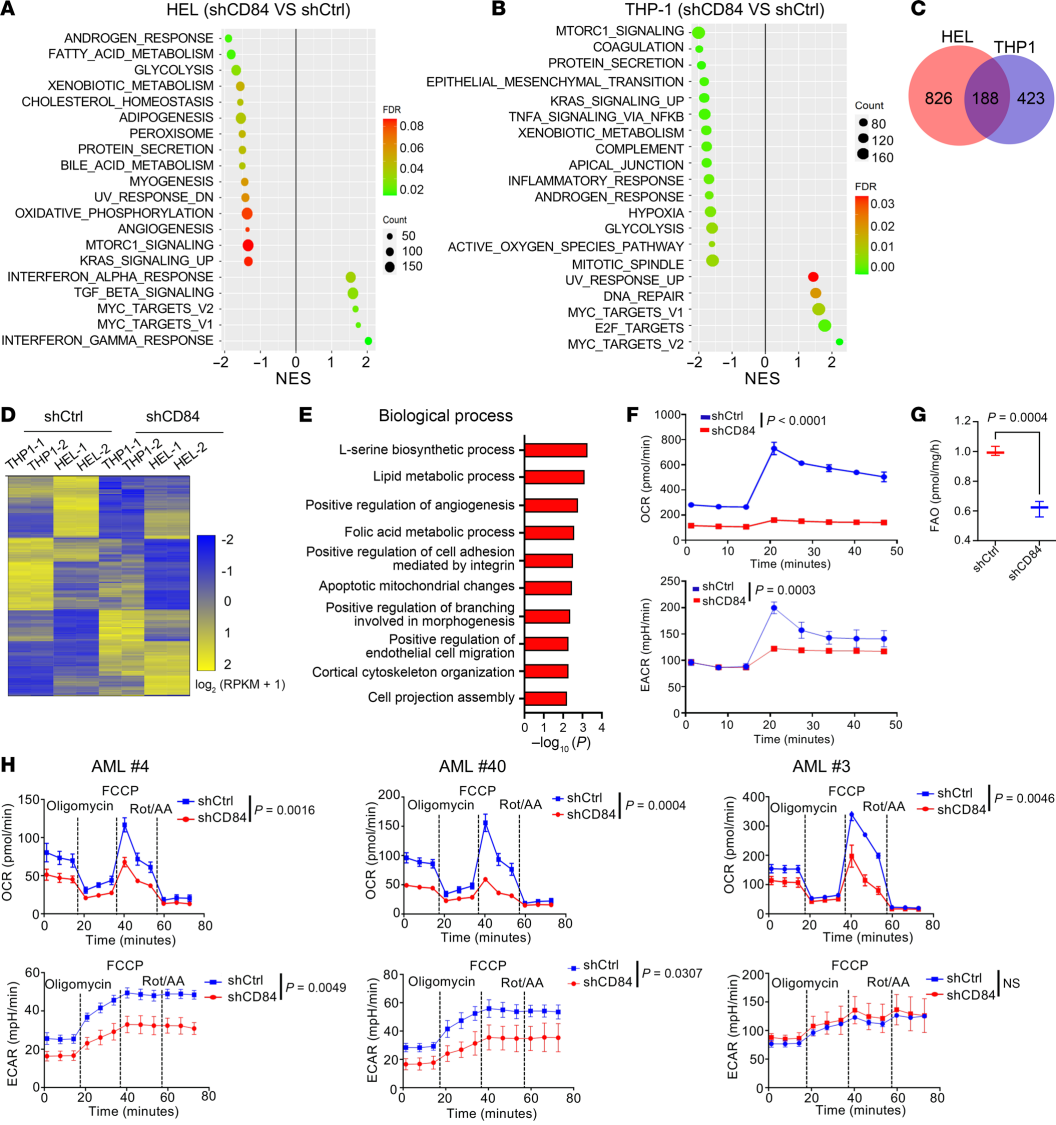
CD84 knockdown deactivated energy metabolism and induced mitochondrial stress in AML. (**A** and **B**) Scattergrams of CD84-related gene sets based on enrichment analyses of DEGs in HEL cells (shCD84 vs shCtrl) (**A**) and THP1 cells (shCD84 vs shCtrl) (**B**). The color indicates the false discovery rate *q* values; NES, normalized enrichment score. (**C**) Venn diagram showing the overlapped DEGs between HEL (shCD84 versus shCtrl) and THP1 (shCD84 versus shCtrl) groups. (**D**) Heatmap showing gene expression of the overlapped differential genes between THP1 cells and HEL cells expressing shCD84 or shCtrl, based on a fold change >2 or <0.5 and *P* < 0.05. (**E**) Bar chart showing GO enrichment analysis of common DEGs (*n* = 188) in 2 AML cell lines. (**F**) Connecting lines showing the effects of CD84 deletion on levels of OCR and ECAR in THP1 cells. Cells were transfected with lentivirus expressing shCD84 or shCtrl, and puromycin selected for 2 days. The cells were harvested to measure levels of OCR and ECAR using the Seahorse XF Cell Energy Phenotype Test Kit. Data are represented as mean ± SEM and are representative of 3 biological replicates. Statistical significance was assessed with 2-way ANOVA (mixed model). (**G**) Box chart showing the effects of CD84 knockdown on FAO levels in THP1 cells. The cells were harvested as described in **F**, and FAO assay results are presented as fold change, compared with control. Data are represented as mean ± SEM and are representative of 3 independent experiments. Statistical significance was assessed by 2-tailed unpaired *t* test. (**H**) Connecting lines showing the effects of CD84 deletion on levels of OCR and ECAR in primary AML cells obtained from *n* = 3 different donors. Cells were transduced with lentivirus expressing shCD84 or shCtrl for 48 hours. The cells were harvested to measure levels of OCR and ECAR using the Seahorse XF Cell Energy Phenotype Test Kit. Data are represented as mean ± SEM and are representative of 3 independent experiments. Statistical significance was assessed with 2-way ANOVA (mixed model).

To further elucidate the molecular underpinnings of CD84 in leukemia cells, we induced alterations in the expression of endogenous CD84 and performed RNA-Seq. Specifically, we transfected with shCtrl or shCD84 lentivirus (Figure 6, A and B) 2 AML cell lines (HEL and THP1) that maintain high CD84 expression. Gene set enrichment analysis (GSEA) showed that CD84 knockdown in both lines caused downregulation of gene sets involved in energy metabolic pathways (Supplemental Figure 5, A and B), including fatty acid metabolism, glycolysis, and oxidative phosphorylation, especially in HEL cells. As further emphasis of the common signature associated with CD84 downmodulation, all differentially expressed genes (DEGs) identified in the shCD84 versus shCtrl groups in the 2 cell lines and 188 common genes were found (Figure 6, C and D). Gene Ontology (GO) enrichment analysis indicated that small molecule metabolic pathways including amino acid metabolism and lipid metabolic processes were downregulated upon CD84 knockdown (Figure 6E and Supplemental Figure 5C).

**Figure 7:**
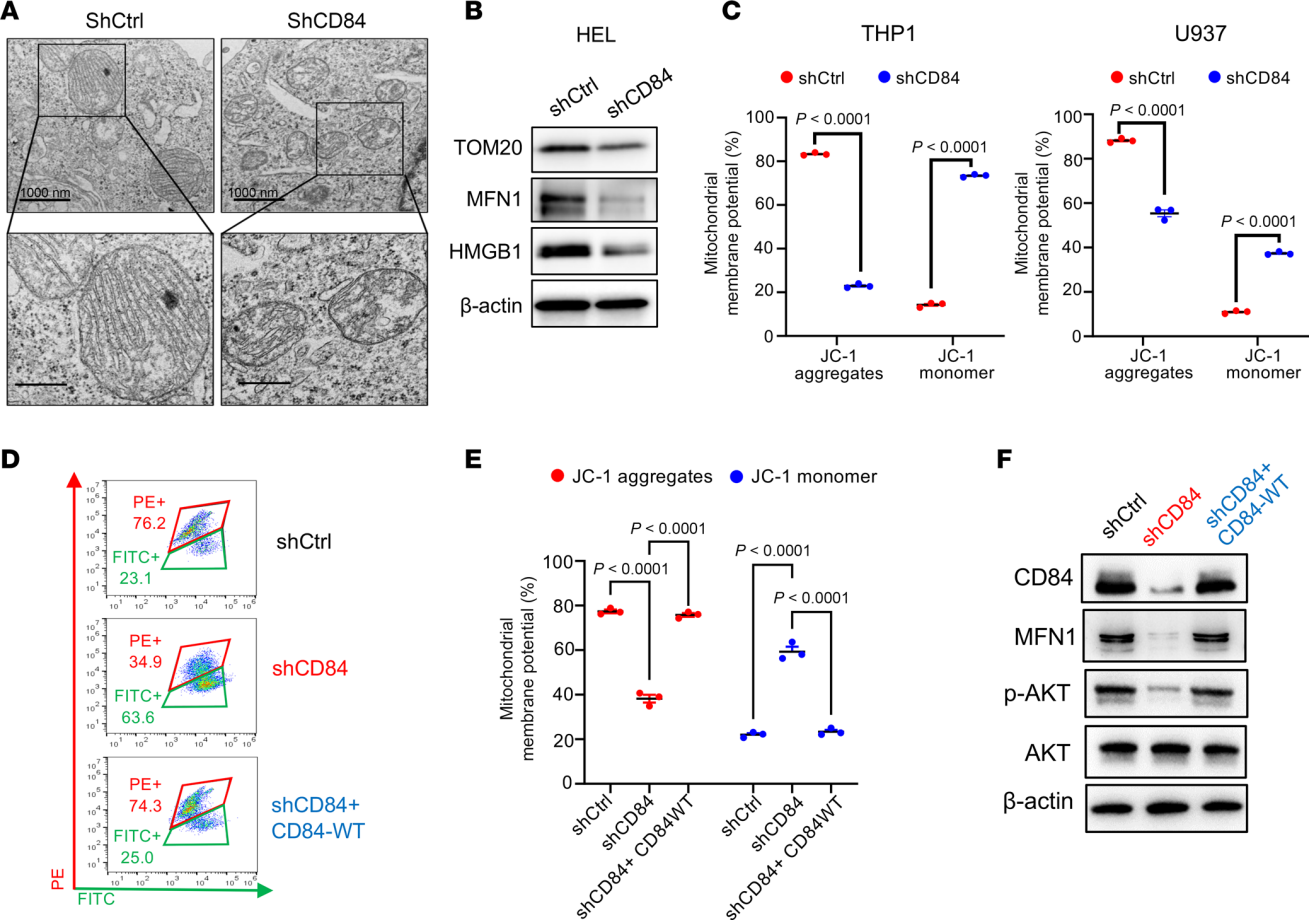
Knockdown of CD84 triggers mitochondrial stress in AML cells. (**A**) Representative transmission electron microscopy images of mitochondrial cristae in HEL cells transfected with shCtrl or shCD84 lentivirus. Scale bars: 1 μm (top); 0.5 μm (bottom). The analysis was conducted in at least 2 independent experimental sets. (**B**) Western blot of indicated proteins was performed in HEL cells transduced with lentivirus expressing either shCtrl or shCD84, indicating mitochondrial dysfunction in CD84 knockdown cells. Data are representative of 2 independent biological replicates. (**C**) Interleaved scatter plot showing the MMPs that were measured using JC-1 dye for flow cytometry. Data are represented as mean ± SEM and are representative of 3 independent experiments. Statistical significance was assessed by 2-tailed unpaired *t* test. (**D**) Representative flow cytometry profiles of JC-1–stained THP1, which was transduced with lentiviruses expressing indicated vectors (shCD84 targeting 3′-UTR). Red (PE) and green (FITC) represent the monomers to aggregated ratio. (**E**) Interleaved scatter plot summarizing the alteration of MMPs shown in **D**. Data are represented as mean ± SEM and are representative of 3 independent experiments. Statistical significance was assessed by 1-way ANOVA. (**F**) Western blot of indicated proteins was performed in THP1 cells transduced with lentivirus expressing either shCtrl, shCD84 (3′-UTR), or shCD84 (3′-UTR) plus CD84-WT. Data are representative of at least 2 independent biological replicates.

Altogether, these results demonstrate that CD84 may orchestrate AML cell survival through modulating energy metabolic reprogramming. To define the role of CD84 in regulating mitochondrial function, we examined alteration of mitochondrial fitness including oxygen consumption rate (OCR), extracellular acidification rate (ECAR), fatty acid oxidation (FAO), mitochondrial morphology, mitochondrial membrane potential (MMP), and mitochondrial biogenesis upon CD84 deletion. CD84 depletion caused mitochondrial dysfunction, as indicated by decreasing OCR and ECAR (Figure 6F) and FAO (Figure 6G) in an AML cell line. Importantly, we also validated the attenuated OCR and ECAR in 3 AML primary cells following CD84 downregulation (Figure 6H). Next, we investigated the effects of CD84 deletion on mitochondrial dynamics in AML cells. Accordingly, we found that CD84 deletion caused disruption of mitochondrial matrix morphology and loss of mitochondrial cristae (Figure 7A). Moreover, CD84 knockdown substantially attenuated TOM20 (mitochondrial marker), MFN1 (mitochondrial fusion marker), and HMGB1 expression levels, which indicated mitochondrial dysfunction upon CD84 deletion in AML (Figure 7B). Additionally, MMP is an indicator of mitochondrial function, and loss of MMP often suggests mitochondrial dysfunction (32). Flow cytometry analysis of JC-1 staining demonstrated a substantially decreased intensity of aggregates and increased intensity of monomers, indicating a substantial loss in MMP and resultant mitochondrial dysfunction in CD84-deleted cells (Figure 7C and Supplemental Figure 5D). Importantly, the reintroduction of CD84 expression partially rescued mitochondrial dysfunction as indicated by MMP (Figure 7, D and E). CD84 overexpression also rescued MFN1 protein downregulation and, as previously published (20), phosphorylation of AKT (p-AKT) (Figure 7F). Collectively, these investigations present compelling evidence that CD84 plays a pivotal role in regulating the survival of AML through orchestrating energy metabolism and inducing mitochondrial stress.

### CD84 knockdown impairs GSH metabolism and NRF2 antioxidant defense, leading to mitochondrial dysfunction in AML.

**Figure 8:**
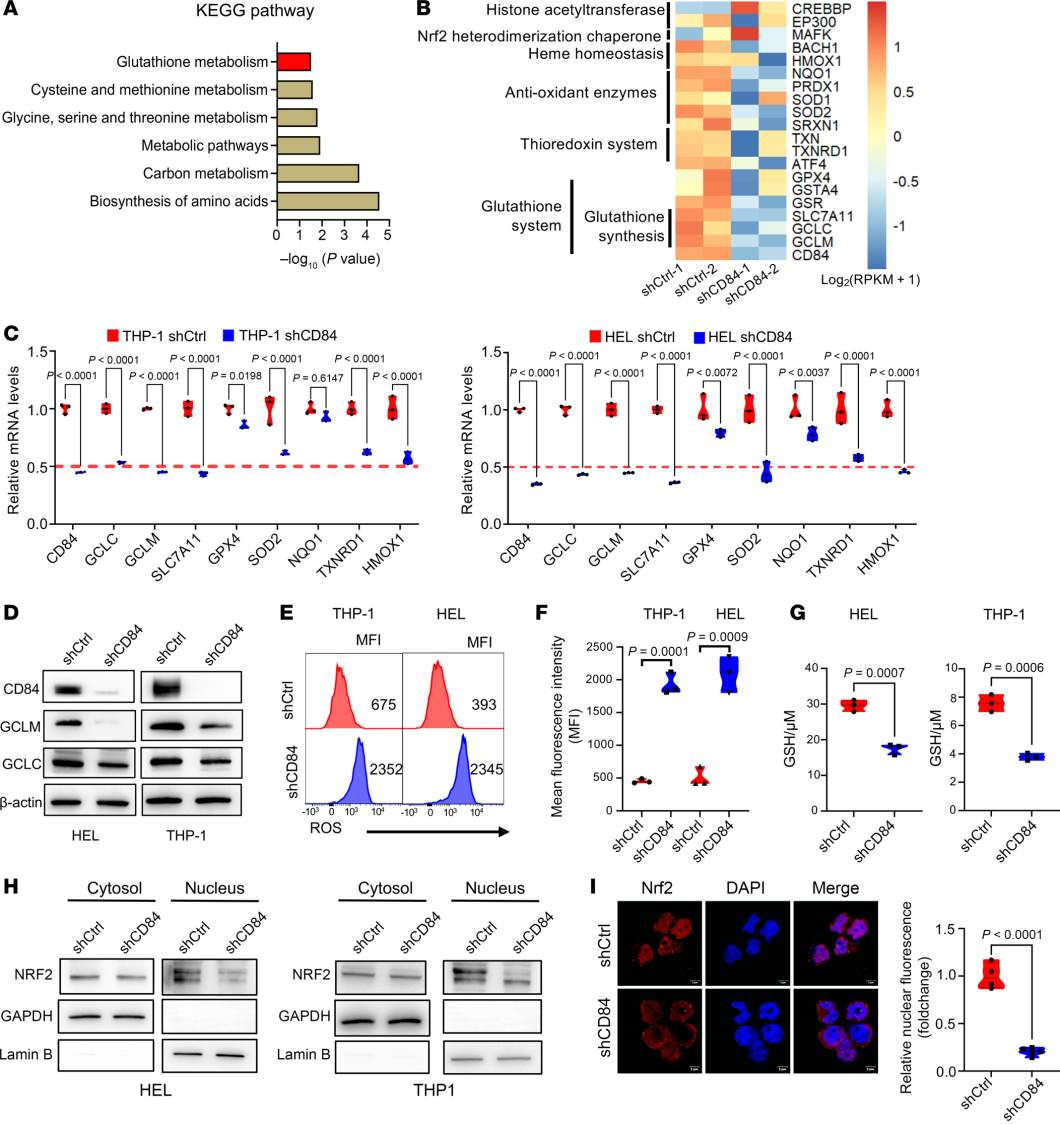
CD84 knockdown impairs GSH metabolism and NRF2 antioxidant defense, leading to mitochondrial dysfunction in AML. (**A**) Bar chart showing the KEGG pathway enrichment analysis of differentially expressed core genes in both THP1 cells and HEL cells. (**B**) Heatmap visualization of NRF2-regulated antioxidant and detoxification enzyme expression according to our RNA-Seq dataset. (**C**) The violin plot showing the mRNA expression of key antioxidant/detoxification genes in THP1 cells and HEL cells transduced with shCtrl or shCD84. Data are represented as mean ± SEM and are representative of 3 independent experiments. Statistical significance was assessed by 2-tailed unpaired *t* test. (**D**) Immunoblot detection of indicated proteins involved in GSH biosynthesis in THP1 cells and HEL cells transduced with shCtrl or shCD84 for 72 hours. Data are representative of at least 2 independent biological replicates. (**E** and **F**) Representative histogram (**E**) and violin chart (**F**) showing the effects of CD84 knockdown on intracellular ROS generation in THP1 cells and HEL cells transduced with shCtrl or shCD84 for 72 hours. Data are represented as mean ± SEM and are representative of 3 independent experiments. Statistical significance was assessed by 2-tailed unpaired *t* test. (**G**) The violin plot shows the intracellular GSH levels in THP1 cells and HEL cells that were transduced with shCtrl or shCD84 for 72 hours. Data are represented as mean ± SEM and are representative of 3 independent experiments. Statistical significance was assessed by 2-tailed unpaired *t* test. (**H**) Immunoblot analysis of the expression of NRF2 in the cytoplasm and nucleus of HEL and THP1 cells stably expressing CD84 shRNA (targeting 3’UTR). Data are representative of at least 2 independent experiments. (**I**) Representative confocal microscopy images and violin chart showing the nucleoplasm distribution of NRF2 in THP1 cells transduced with either shCtrl or shCD84 lentivirus. The intensity of nuclear fluorescence was quantified in the violin plot. Data are represented as mean ± SEM and are representative of 4 independent images. Statistical significance was assessed by 2-tailed unpaired *t* test.

To further investigate the underlying mechanism associated with mitochondrial dysfunction induced by CD84 deletion, we further performed Kyoto Encyclopedia of Genes and Genomes (KEGG) pathway analysis and observed that the GSH metabolism pathway was highly enriched upon CD84 knockdown (Figure 8A). We found and further validated that almost all the genes involved in the GSH metabolism were consistently downregulated upon CD84 knockdown, including key genes involved in GSH synthesis, such as glutamate-cysteine ligase catalytic subunit (GCLC) and glutamate-cysteine ligase modifier subunit (GCLM), among others (Figure 8, B and C). Consistently, the protein expression of GCLM and GCLC were robustly decreased in CD84 knockdown cells (Figure 8D). In cancer cells, a high level of GSH is indispensable to scavenge excessive ROS and detoxify xenobiotics, which make it a potential target for cancer therapy (33, 34). Our results exhibited that CD84 knockdown resulted in enhanced ROS generation (Figure 8, E and F) and reduced levels of GSH (Figure 8G) in AML cells. These results are aligned with the well-known concept that GSH downregulation is associated with impairment of the electron transport chain (ETC) in mitochondria (35). Because GSH metabolism genes including GCLC and GCLM are downstream targets of NRF2 (36) and CD84 prosurvival activity has been linked to SHIP-1–AKT phosphorylation (21), which in turn has been associated with NRF2 transcriptional activation (37–40), we investigated whether changes in CD84 expression may affect NRF2 activity. We observed that CD84 knockdown in AML cells decreased total NRF2 protein levels (Supplemental Figure 6A) and its nuclear localization (Figure 8, H and I).

**Figure 9:**
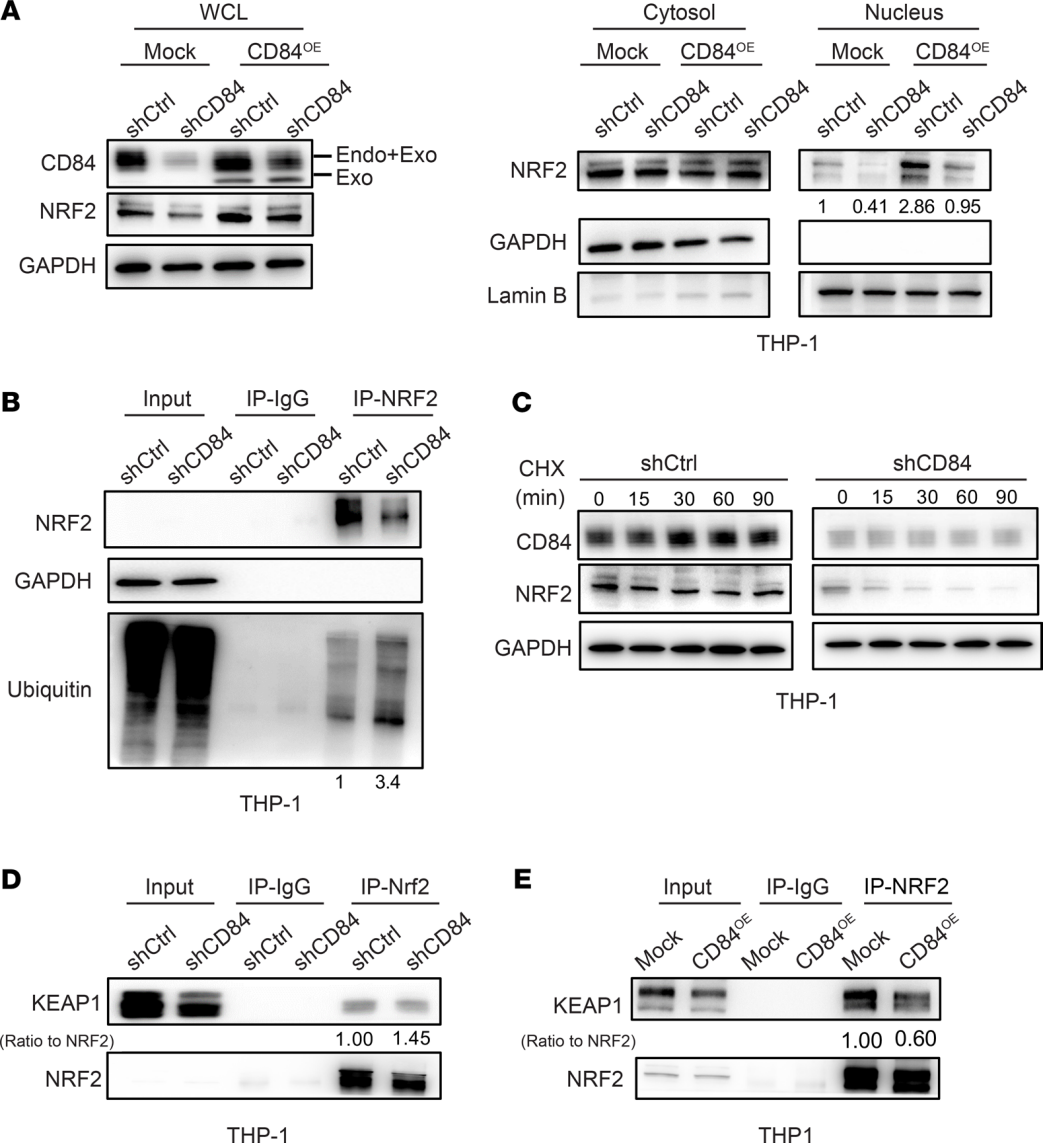
CD84 knockdown disrupts NRF2 binding to Keap1 in AML cells. (**A**) The immunoblot shows the expression of indicated proteins in THP1 cells transduced with shCtrl or shCD84. THP1 cells stably expressing 3xFlag-CD84 were further infected with viruses expressing CD84 shRNA. The amount of NRF2 in the whole cell lysate, cytoplasm, and nucleus was determined by immunoblot. (**B**) The immunoblot shows coimmunoprecipitation analysis of the ubiquitination of NRF2 upon CD84 knockdown in THP1 cells. (**C**) The immunoblot shows the time course of protein expression after CHX treatment at indicated times. Western blot analysis confirmed the presence of NRF2 at times after CHX treatment in control samples. (**D** and **E**) The immunoblots show quantitative analysis of the binding to KEAP1 in the presence or absence of stably overexpressing Flag-CD84 cells by immunoprecipitation. The interaction between NRF2 and KEAP1 under different CD84 levels was analyzed. Indicated THP1 cells stably expressing CD84 shRNA (**D**) or 3xFlag-CD84 (**E**) were harvested for immunoprecipitation and subjected to immunoblotting with anti-NRF2 and anti-KEAP1 antibodies. Data in **A**–**E** are representative of at least 2 biological replicates.

The decreased expression and nuclear distribution of NRF2 induced by knockdown of CD84 were rescued by stably overexpressing CD84 (Figure 9A), suggesting that CD84 is critical for maintaining the nuclear translocation of NRF2. NRF2 is a transcription factor that coordinates the basal and stress-inducible activation of a vast array of cytoprotective genes through AREs. To fully assess the functional status of the NRF2-ARE pathway, we measured its transcriptional activity using ARE-driven luciferase constructs. We observed that stable overexpression of CD84 activated ARE-regulated luciferase in HEK 293T cells compared with cells transduced with EV, and the elevated activity was statistically significantly reduced subsequently by CD84 knockdown (Supplemental Figure 6, B and C). Because NRF2 protein stability and nuclear translocation are tightly regulated by mechanisms of ubiquitination (41), we investigated whether CD84 knockdown could promote NRF2 ubiquitination and subsequent degradation. Immunoprecipitation assays showed a concomitant decrease in NRF2 but an increase in its ubiquitination upon shCD84 (Figure 9B and Supplemental Figure 6D). Western blot analysis showed that the treatment of the protein synthesis inhibitor cyclohexamide (CHX) further decreased protein levels of NRF2 in the shCD84 group, based on elevated proteolytic degradation (Figure 9C and Supplemental Figure 6E). Because nucleoplasm distribution and protein stability of NRF2 is an important regulatory event that is tightly controlled by its association with a cytosolic inhibitor protein, KEAP1 (42), a sensor protein that targets NRF2 for ubiquitination by a Cullin-3–dependent mechanism and leads to proteosome-dependent degradation (43), we investigated KEAP1 binding to NRF2 upon CD84 modulation. Immunoprecipitation data showed that upon CD84 knockdown there was an increase in NRF2-KEAP1 binding associated with NRF2 protein downregulation (Figure 9D and Supplemental Figure 6F), an effect that was reverted upon CD84 overexpression (Figure 9E and Supplemental Figure 6G). These findings provide a mechanism in that CD84 is involved in maintaining NRF2 transcription activity and the mitochondrial antioxidant system in AML.

## Discussion

CD84 is a hematopoietic marker that is variably expressed on distinct subsets of B and T cells, but it is also observed to be consistently expressed at high levels on monocytes, macrophages, dendritic cells, platelets, and MDSCs (23, 44). We show that CD84 is a hematopoietic lineage marker and its expression is absent in normal tissues. Here, we show that normal HSCs and MPPs are also negative for CD84 and that during the hematopoietic differentiation process, CD84 begins to be expressed at high levels in CMPs. Although AML is characterized by the accumulation of CMPs, the role of CD84 in this disease setting has not been investigated thus far. Previously published data have shown that CD84 positively regulates LPS-induced cytokine secretion through MAPK phosphorylation and NF-κB activation in macrophages (44), 2 survival pathways that are critical to support AML progression and drug resistance (45–50). Consistent with previously published data showing the pivotal role of CD84 in supporting CLL cell survival and growth (20, 21), our data suggest that CD84-dependent survival effects appear to be even more prominent in AML. Gene disruption via shRNA in AML lines, AML primary patient cells, and murine models showed that CD84 depletion robustly hampered AML cell survival, blast clonogenicity, and leukemic engraftment. In support of the idea that CD84 expression confers strong survival advantages to AML cells, we observed that mice xenografted with CD84-overexpressing cells had substantially lower survival compared with control animals, an effect that was completely reverted by CD84 knockdown in the same experimental setting. Although a delay of symptomatic disease was observed in AML xenograft models transplanted with either AML cell lines or PDXs carrying shCD84 knockdown, at the time of relapse, AML cells completely bypassed CD84 silencing, further supporting the clonal advantage associated with the expression of high CD84 levels in leukemia cells.

We found direct correlation between CD84 mRNA expression and survival of AML patients in 2 out of 4 mRNA-Seq data sets. Interestingly, conversely to AML cell lines, we did not find direct correlation between CD84 surface expression and mRNA in primary AML samples obtained from different sources. These data may suggest that AML cell heterogenicity both in terms of genetic and cellular composition and blast cell purity may be responsible. This concept is also supported from our immune IHC analysis, in which we found in all the AML samples analyzed almost 100% of the blasts are highly positive for CD84, but this positivity was somehow diluted when the samples were analyzed by both flow cytometry and CyTOF. We acknowledge that our IHC analysis is based on a limited number of samples and needs further investigation.

Consistent with these observations, shRNA targeting CD84 in 2 preleukemic AML mouse cells, MLL-AF9 and inv(16) AML, inhibited cell viability and delayed leukemic onset in recipient mice. Notably, in both immune-competent AML mouse models, CD84 upregulation was observed only in leukemic c-kit^+^ but not in the healthy counterpart. With the intent to generate stable AML clones that maintained CD84 downregulation, our data show that, when CD84 was completely downmodulated using a double shCD84 targeting, mouse leukemia cells lost their clonogenic capabilities, resulting in a complete absence of colonies, further supporting that CD84 upregulation is essential for the leukemogenesis process and its role is conserved through the species.

Recently, therapeutically targeting the metabolic vulnerability of leukemia cells through mitochondrial alterations has attracted much interest in the AML community (52, 53). This interest is based on the dependence of AML cells on oxidative phosphorylation (53, 54) and fatty acid metabolism (54), their ability to tolerate higher ROS levels (9, 56, 57), and their low tolerance to the downregulation of antioxidant enzymes (9). In agreement with these data, GSEA enrichment analysis revealed that CD84 downregulation in AML cells affects metabolic processes involving mitochondria function. In fact, it is reported that AML progression requires increased mitochondrial biogenesis and oxidative phosphorylation (54) and that the quiescent LSCs are more dependent on oxidative phosphorylation, as they cannot efficiently utilize glycolysis for energy homeostasis (58, 59). In CLL, CD84 is reported as a positive regulator of antiapoptotic genes, such as BCL2 and MCL1 (21), which is also associated with mechanisms of tolerance to oxidative stress (60). GSEA enrichment analysis revealed that CD84 downregulation in AML cells substantially affects metabolic processes involving mitochondrial function, such as fatty acid metabolism and oxidative phosphorylation. CD84 knockdown downregulates AKT phosphorylation, alters the structure of mitochondria, disrupts mitochondrial respiration, and decreases oxidative phosphorylation. These observations suggest that impairing CD84 activation pathways could be therapeutically beneficial in the treatment of patients with AML. In a mechanism study, we observed that CD84 appears to play a pivotal role in maintaining GSH metabolism and NRF2 antioxidant defense in leukemia cells causing ROS accumulation. Knockdown of CD84 decreases NRF2 nuclear localization and transcriptional activity of antioxidant genes, increases oxidative stress, and promotes NRF2 degradation via the KEAP1 interaction. Although further studies are needed to identify further key components and specifically characterize the cascade of events by which CD84 can regulate NRF2 degradation, to the best of our knowledge, we show for the first time that CD84 plays an essential role in regulating AML metabolism and oxidative phosphorylation, highlighting a dependency of AML to CD84 expression. Notably, we (17) and others (17, 23) have recently identified CD84 to be highly expressed in MDSCs. Interestingly MDSCs can produce high levels of ROS to fulfill their immune-suppressive activity, but their viability remains unaffected mainly through NRF2-driven antioxidant capacity (61–63). Although the biological function of CD84 on the surface of MDSCs has not been yet elucidated, we can speculate that, in this heterogeneous myeloid cell population as well, CD84 may be crucial in empowering an antioxidant defense to preserve cellular viability, an observation that needs further research.

In conclusion, we show that CD84 is required for AML cell survival and leukemogenesis. Mechanistically, we reveal that CD84 regulates AML survival through modulating NRF2 transcriptional activity involved in the mitochondrial antioxidant system. Finally, we identify CD84 as a critical regulator of mitochondrial oxidative stress, highlighting a therapeutic vulnerability of AML cells.

## Methods

### Sex as a biological variable.

Our study examined male and female animals, and similar findings are reported for both sexes.

### Cell culture.

AML primary patient cells were cultured in Stemspan Serum-Free Medium (Stem Cell Technologies), supplemented with low concentrations of growth factors (GFs) similar to those present in long-term BM stromal cell culture (200 pg/mL granulocyte-macrophage colony-stimulating factor [GM-CSF], 50 pg/mL leukemia inhibitory factor [LIF], 1 ng/mL granulocyte colony-stimulating factor [G-CSF], 200 pg/mL stem cell factor [SCF], 200 pg/mL macrophage inflammatory protein-1α [MIP-1α], and 1 ng/mL IL-6). The THP1, SKM1, HEL (HEL 92.1.7), NOMO1, U937 (provided by a laboratory at City of Hope), and MV-4-11 (purchased from ATCC, CRL-9591) cell lines and the multiple myeloma cell line MM1S were maintained in RPMI 1640 with 10% FBS, penicillin, streptomycin, and glutamine (all Gibco-BRL, Thermo Fisher Scientific). The HEK-293T line was maintained in DMEM with 10% FBS, penicillin, streptomycin, and glutamine (all Gibco-BRL, Thermo Fisher Scientific). MLL-AF9 AML cells were cultured in IMDM with 10% FBS, penicillin, streptomycin, and glutamine (all Gibco-BRL, Thermo Fisher Scientific) supplemented with 2 ng/mL IL-3. *Inv* (16) AML cells were cultured in IMDM with 20% FBS, penicillin, streptomycin, and glutamine (all Gibco-BRL, Thermo Fisher Scientific) supplemented with 20 ng/ml SCF, 20 ng/ml thrombopoietin, 10 ng/mL IL-3, and 6 ng/ml IL-6. Cells were grown at 37°C in an atmosphere containing 5% CO_2_.

### Lentivirus transduction of cell lines.

Lentivirus pseudotyped particles were produced by Lipofectamine 2000–mediated (Life Technologies) transfection of 293T cells with the packaging construct psPAX2, a plasmid carrying G-glycoprotein of vesicular stomatitis virus (VSV-G), and the lentivirus vectors including MSCV-Luciferase-EF1α-copGFP-T2A-Puro (System Biosciences, SBI), pMIG-FLAG-MLL-AF9 (Addgene), pCDH-EF1α-MCS-T2A-GFP (Addgene), pCDH-EF1α-MCS-T2A-GFP-CD84 (GenScript Biotech), PLKO.1-puro-shCD84-1(TRCN0000057474; CGCTACAACCTGCAAATCTAT; human; MillporeSigma), PLKO.1-puro-shCD84-2 (TRCN0000371708; TTATGGCACACTGGGATAAAC; human; MillporeSigma), PLKO.1-puro-shCD84-1 (TRCN0000066279; GCAGACATCAATGAAGAGAAT; mouse; MillporeSigma), and PLKO.1-puro-shCD84-1 (TRCN0000066280; GCAGATGATGTCTCAAAGAAA; mouse; MillporeSigma). Viral supernatants were harvested at 48 and 72 hours after transfection and filtered through a 0.45 mm low protein binding membrane (Millipore). Cells were exposed to virus-containing supernatant (MOI = 5–10) via spinoculation and then sorted by flow cytometry based on GFP or selected by puromycin selection (1 μg/ml).

### MLL-AF9 retrovirus packaging.

HEK293T cells were plated overnight in a T75 flask at a density of 8 millions cells per flask. The next day, cells were transfected with 10 μg of pMIG-FLAG-MLL-AF9 (Addgene, catalog 71443) and 7 μg of pCL-ECO plasmids using Lipofectamine 3000 transfection reagent for 8 hours in Opti-MEM medium (Thermo Fisher, catalog 31985062). At 8 hours after transfection, Opti-MEM medium was replaced with complete DMEM medium. Viral containing supernatant was collected at 48 and 72 hours and concentrated with Retro-Concentin (SBI, catalog RV100A-1) for 72 hours at 4°C and virus pellet was resuspended in 1× DPBS (dulbecco’s phosphate buffered saline) and frozen.

### Transduction of c-Kit^+^ cells with MLL-AF9 retrovirus.

C57BL/6 mice were humanely euthanized, and femurs, tibias, and spine were harvested and crushed to collect the mononuclear cells (MNCs). Following the manufacturer’s protocol (Miltenyi Biotec, catalog 130-091-224), c-Kit–positive cells were isolated from MNCs and transduced with MLL-AF9 retrovirus as described previously with modifications (64). Briefly, a nontreated 24-well plate was coated with 20 μg/ml RetroNectin (Takara, catalog T100A) overnight at 4°C. Following overnight incubation, RetroNectin was washed, and the plate was blocked with 2% BSA/1× DPBS for 30 minutes at room temperature (RT) and MLL-AF9 retrovirus was spinoculated for 2 hours at 1,000*g* at 4°C. The spinoculation with viral supernatant was repeated at least 3 times. Following spinoculation, viral supernatant was aspirated and c-Kit^+^ cells were added to the plate and spinoculation was repeated for 10 minutes at RT. At 24 hours, MLL-AF9-c-Kit^+^ transduced cells were recollected and added to a new nontreated 24-well plate that was coated with RetroNectin and spinoculated twice with MLL-AF9 retrovirus. c-Kit^+^ cells were resuspended in 20% FBS/1% penicillin-streptomycin IMDM medium with the following cytokines: 20 ng/ml murine IL-3 (GeminiBio, catalog 300-324P-100), 20 ng/ml murine IL-6 (Invitrogen, catalog RMIL6I), 60 ng/ml murine SCF (Invitrogen, catalog RP-8632), and 10 μg/ml of polybrene. At 48 hours, MLL-AF9 GFP^+^ c-Kit^+^ cells were transduced with shCtrl and shCD84 lentivirus for 48 hours at MOI = 5. At 48 hours after shCtrl and shCD84 transduction, cells were collected for apoptosis analysis and colony-forming assay.

### Flow cytometry analysis.

Cells were washed with 1× PBS and stained for 30 minutes in ice-cold FACS buffer (PBS+2%FBS) using antibodies (anti-human CD84-PE, BioLegend, catalog 326008; anti-human CD45-APC, BioLegend, catalog 368512; anti-human CD33-FITC, eBioscience, catalog 366619; anti-mouse CD45.1, BioLegend, catalog 110707; anti-mouse CD45.2, BD, catalog 565390; anti-mouse CD84-PE, BioLegend, catalog 122806). After 30 minutes, cells were washed and analyzed on an LSRII (Becton Dickinson) or BD LSR Fortessa X-20 (Becton Dickinson). Analysis was conducted using FlowJo Software (version 10.7.1). The stained samples were analyzed on a BD LSR Fortessa X-20 (Becton Dickinson). Analysis was conducted using FlowJo Software (version 10.7.1).

### Analysis of cell viability, apoptosis, and colony formation assay.

Cell growth was measured utilizing the CellTiter-Glo Luminescent Cell Viability Assay Kit (Promega). Cell proliferation was determined using dye eFluor 670 staining (eBioscience) followed by flow cytometry analysis. Apoptosis was assessed based on annexin V/DAPI staining (eBioscience). For colony-forming assay, cells were resuspended in 2% IMDM at concentration of 0.1 million cells/ml, 100 μl of this suspension was added to 1 well of a 24-well plate, and each treatment was plated in at least duplicates and repeated at least 3 times. Human cells were overlaid with 750 μl of MethoCult H4034 (Stem Cell Technologies), and murine cells were overlaid with 750 μl of MethoCult GF M3434 (Stem Cell Technologies, catalog # 03444). Colonies were analyzed on day 14 using a Widefield Zeiss Observer 7 inverted microscope in tiles at the City of Hope Light Microscopy Imaging Core.

### Seahorse assay.

A total of 40,000 cells in 200 μL cell culture medium was seeded in each well of an XF-96-well cell culture microplate (Seahorse Bioscience) and cultured overnight at 37°C in 5% CO_2_. As a negative control, 3 wells were kept devoid of cells and given only Seahorse media, which comprises basal XF media, 5.5 mM glucose, 1 mM sodium pyruvate, and 4 mM glutamine. (Additionally, the pH was adjusted to 7.4.) Twelve hours prior to running a plate, the Seahorse sensor cartridge was incubated with Seahorse Calibrant solution according to the manufacturer’s protocol, in a 37°C, CO_2_-free incubator. On the day of an assay, shCtrl and shCD84 cells were washed and incubated with Seahorse media. The sensor cartridge was fitted onto the cell culture plate, which was then placed into a 37°C, CO_2_-free incubator for 1 hour. During the assay, which was run on the Seahorse XF96 Analyzer, the following inhibitors were injected sequentially, as is standard for the Cell Energy Test: oligomycin (1 mM) and carbonyl cyanide p-trifluoromethoxyphenylhydrazone (0.5 mM).

### ROS and GSH measurement.

THP1 and HEL cells transfected with shCtrl or shCD84 were washed with 1× PBS and then incubated with 5 μM CellROX Oxidative Stress Reagents (Invitrogen, catalog C10422) for 30 minutes at 37°C in the dark. After incubation, cells were washed twice with 1× PBS and analyzed on a CytoFLEX LX flow cytometer (Beckman Coulter). Data were analyzed using FlowJo Software (version 10.7.1). For GSH measurement, the levels of GSH were assessed using a commercially available kit (Beyotime, catalog S0053) according to the manufacturer’s instructions. Briefly, cell samples were subjected to 2 rapid freeze-thaw cycles using liquid nitrogen and a 37°C water bath. Corresponding detection reagents were added to an appropriate volume of cell lysates. After incubation for 25 minutes, GSH content was measured using a microplate reader at an absorbance of 412 nm. GSH levels were quantified by comparing the absorbance values to a standard curve.

### Immunofluorescence staining.

The cultured cells were fixed in 4% paraformaldehyde (PFA) for 25 minutes in the dark at RT. After fixation, cells were permeabilized and blocked with 3% donkey serum in PBS containing 0.2% Triton X-100 for 60 minutes at RT. After centrifugation at 1,000*g* for 5 minutes, cells were incubated with primary antibodies (NFE2L2 polyclonal antibody, Proteintech, catalog 16396-1-AP) overnight at 4°C. The next day, cells were incubated with the appropriate secondary fluorescently labeled antibodies (CoraLite594-conjugated goat anti-rabbit IgG[H+L], Proteintech, catalog SA00013-4) for 1 hour in the dark at RT. After incubation, the samples were mounted using ProLong Gold Antifade Mountant with DAPI (Invitrogen, catalog P36941). Imaging was performed using a laser scanning confocal microscope (OLYMPUS, FV3000).

### Morphological analysis.

In brief, cells were extracted from BM and diluted in PBS to 2 × 10^5^/ml. After they were spread onto slides, cells were fixed in absolute methanol and stained in Wright-Giemsa stain solution (Sigma). Smears were then rinsed in pH 6.6 phosphate buffer solution. Images were acquired using Zeiss Observer 7.

### RNA-Seq analysis.

THP1 and HEL cells with shCtrl or shCD84 were selected by puromycin selection. Cells were collected and resuspended in TRIzol reagent (Invitrogen) following the manufacturer’s instructions. RNA quality (RNA integrity number [RIN]) was assessed using an Agilent Bioanalyzer, and all samples were evaluated as RIN > 8. RNA-Seq libraries were prepared with Kapa RNA HyperPrep kit with polyA kit (Kapa Biosystems, catalog KR1352) according to the manufacturer’s protocol. A sequencing run was performed in the single-read mode using Illumina HiSeq 2500. Sequenced reads were aligned to the mouse hg38 reference genome with TopHat2 (version 2.0.14). Gene-expression level was quantified using HTSeq (version 0.6.1), and differential expression analysis was performed using DESeq2 (version 1.14.1).

### Mass cytometry (CyTOF) staining and acquisition.

BM or PB MNCs from AML patients were thawed in customized thawing medium (20% FBS, 0.06 mg/ml DNAse I, and 20,000 U heparin in IMDM) for 2 hours at RT to obtain single cells. Following 2 hours of incubation, AML MNCs were washed and suspended in IMDM supplemented with 20% FBS. A total of 2–4 × 10^6^ BM-MNCs were stained with a custom panel of 39 metal-conjugated antibodies for surface markers along with Cell-ID Cisplatin for nonviable cell detection (Supplemental Table 1). Staining protocols provided by Standard BioTools Inc. were followed for Cell-ID Cisplatin (PRD018 version 5) (catalog 201064) and Maxpar Cytoplasmic/Secreted Antigen Staining with Fresh Fix (400279 Rev 05). Purified antibodies were purchased from BioLegend and conjugated in-house using Maxpar Antibody Labeling (PRD002 Rev 12) from Standard BioTools. Stained samples were acquired on a Helios mass cytometer (Standard BioTools).

### CyTOF data cleanup and analysis.

Data from the Helios mass cytometer was bead normalized per the manufacturer’s recommendations using standalone CyTOF software, version 7.0.8493.0. For analysis, the FCS files obtained from the custom panel were manually analyzed using 2 software platforms. FlowJo Software (Windows edition, Version 10.6, Becton Dickinson Company; 2019) was utilized for overall cleaning before exporting to the Cytobank platform. The Cytobank platform (Cytobank, Inc.), accessible at https://www.cytobank.org, was employed for further analysis of gating, t-distributed stochastic neighbor embedding (tSNE) plots, and FlowSOM analysis for AML subset populations.

### IHC.

CD84 IHC was performed on a Ventana Discovery Ultra IHC automated stainer (Ventana Medical Systems, Roche Diagnostics). Briefly, the tissue slides were deparaffinized, rehydrated, and incubated with endogenous peroxydase activity inhibitor and antigen retrieval solution. Then, the anti-CD84 primary antibody (Abcam, catalog ab131256) was incubated followed by DISCOVERY anti-rabbit HQ and DISCOVERY anti–HQ-HRP incubation. The stains were visualized with the DISCOVERY ChromoMap DAB Kit, counterstained with hematoxylin (Ventana) and coverslipped. IHC whole slide images were acquired with the NanoZoomer S360 Digital Slide Scanner (Hamamatsu) and viewed by NDP.view image viewer software.

### Nuclear and cytoplasmic protein extraction.

The nuclear and cytoplasmic proteins were obtained using the Nuclear and Cytoplasmic Protein Extraction Kit (Beyotime, P0027) following the manufacturer’s instructions. For cytoplasmic protein extraction, cells were collected and resuspended in 100–200 μL of Cytoplasmic Extraction Buffer, supplemented with protease and phosphatase inhibitors. The lysate was incubated on ice for 10 minutes, vortexed, and centrifuged at 12,000*g* for 10 minutes at 4°C. The supernatant (cytoplasmic fraction) was collected and stored. For nuclear protein extraction, the pellet was resuspended in 50–100 μL of Nuclear Extraction Buffer and incubated on ice for 30 minutes with vortexing. After centrifugation, protein concentrations were determined using a BCA Protein Assay Kit (Thermo Fisher Scientific, catalog 23225).

### Coimmunoprecipitation assay.

THP1 or HEL cells transduced with shCtrl or shCD84 were collected and lysed in NP-40 lysis buffer (0.5M EDTA, 1% NP-40) supplemented with protease inhibitors. The lysates were incubated on ice for 30 minutes and sonicated (10 bursts of 5 seconds on, 5 seconds off) and centrifuged at 12,000*g* for 15 minutes at 4°C. Protein concentrations were determined using a BCA Protein Assay Kit (Solarbio, catalog PC0020). A small aliquot of lysate was saved as input. The remaining supernatant was incubated with the primary antibody (NRF2 polyclonal antibody, Proteintech, catalog 16396-1-AP; KEAP1 monoclonal antibody, Proteintech, catalog 60027-1-Ig; DYKDDDDK tag monoclonal antibody [binds to FLAG tag epitope], Proteintech, catalog 66008-4-Ig) or normal IgG (rabbit: CST, catalog 2729S; mouse: SCBT, catalog sc-2025) overnight at 4°C. Following incubation, the lysate was further incubated with precleaning protein A/G beads (MCE, catalog HY-K0202) for 4 hours at 4°C. After 3 washes with washing buffer (0.5M EDTA, 0.1% NP-40), the beads were resuspended in 60 μl of 1× loading buffer, boiled, and then subjected to SDS–PAGE for further analysis.

### Western blotting analysis.

Briefly, equal amounts of extracts were loaded onto the SDS polyacrylamide gels, electrophoresed, and blotted onto the PVDF membranes (Millipore, catalog IPVH00010). The membrane was blocked with 5% skimmed milk, followed by incubation with primary antibodies at 4°C overnight. Then the membranes were incubated with the HRP-conjugated secondary antibodies and detected using an ECL kit (Beyotime, catalog P0018S). The primary antibodies included lamin B polyclonal antibody (Proteintech, catalog 12987-1-AP), GAPDH monoclonal antibody (SCTB, catalog sc-47724), CD84 monoclonal antibody (Invitrogen, catalog MA5-42775), Gclc polyclonal antibody (Proteintech, catalog 12601-1-AP), Gclm polycloncal antibody (Proteintech, catalog 14241-1-AP), ubiquitin monoclonal antibody (Abclonal, catalog A19686), and actin monoclonal antibody (Proteintech, catalog 66009-1-Ig).

### Establishment of AML PDX for survival analysis following CD84 knockdown.

AML PDX cells with luciferase reporter were provided by a laboratory at City of Hope (PDX-148) (64). Eight million cells were equally divided and transduced with human shCtrl and shCD84 lentiviral particles at MOI = 20 with TransDux MAX Reagent (System Biosciences, catalog LV860A-1) following the manufacturer’s protocol with no modifications for nonadherent cells. Following 2 hours of spinoculation, cells were immediately injected into NSG mice and tumor burden monitored by bioluminescence imaging weekly. Around 200,000 of each treatment group cells were cultured ex vivo to monitor CD84 knockdown.

### Statistics.

All statistical analyses were performed as indicated in each figure using GraphPad Prism (version 9) software. Unpaired Student’s 2-tailed *t* test was used to compare between 2 groups, whereas 1-way ANOVA with multiple comparisons was used to compare multiple groups. The log-rank (Mantel-Cox) test was used to assess statistically significant differences in mouse survival between treatment groups. A *P* value of less than 0.05 was considered statistically significant, and data are presented as mean ± SEM.

### Study approval.

All animal protocols were approved by the Animal Care and Use Committee of the City of Hope National Medical Center, in accordance with the NIH Guidelines for the Care and Use of Laboratory Animals. NSG and C57BL/6 mice were obtained through the animal breeding facility at City of Hope, and CD45.1 mice (B6-Ly5.1) were purchased from Charles River Laboratories. Frozen PB MNCs from AML patients were obtained from the City of Hope Hematopoietic Tissue Biorepository (IRB 18067). All patient characteristics are summarized in Supplemental Table 1. Sample acquisition was approved by the City of Hope Institutional Review Board in accordance with the Declaration of Helsinki. Written, informed consent was received from all participants prior to inclusion in the study. Healthy donor PBMCs were obtained from a leukocyte filter collected through healthy platelet donors at the City of Hope blood donor center.

### Data availability.

All primary data will be made available upon reasonable request. The bulk RNA-Seq data that support this study has been deposited in Gene Expression Omnibus (GEO GSE288016). Values for all data points in graphs are reported in the Supporting Data Values file.

## Author contributions

YZ designed experiments, interpreted results, wrote the manuscript, and performed experiments, including GEO database analysis, gene editing, mouse experiments, RNA-Seq, and flow cytometry; based on her extended contribution to the manuscript writing and on her experimental work she is listed as first co–first author. M Murtadha performed experiments including AML cell line and PDX xenografts, MLL-AF9 and inv(16) c-kit transduction, flow cytometry, colony formation, CD84 mRNA and protein correlation analysis and wrote part of the methods and figure legend sections. She also scientifically edited the manuscript; for this reason she is listed as second co–first author. M Liu performed mechanism studies and scientifically edited the manuscript, but did not contribute to the writing; for this reason she is listed as the third co–first author of the manuscript. EC conducted mouse experiments. ON performed the CD84 and supported with mouse transplantation and colony assays. LXTN performed the Seahorse assay and electron microscope experiment. HW collected AML primary patients’ samples. M Moloudizargari processed and conducted flow cytometry on healthy donor samples. LN performed survival assays using AML cells. TT performed CyTOF analysis. XW and M Murtadha collected AML primary patients’ samples and analyzed the data of CD84 expression in AML. AP assisted in statistical analysis. RS provided the mouse AML cells. XL performed flow cytometry of CD84 expression in AML primary cells. MDE prepared the mouse experiments. RP and JS performed the IHC staining of normal tissue and AML patient samples. JFS revised the manuscript. LZ, M Li, and YHF provided inv(16) mouse preleukemia cells. BZ, LL, YHK, and SR reviewed the manuscript. GM supported the experimental design and reviewed the manuscript. JCW supported the experimental design and contributed to the manuscript writing. FP supported, designed, directed and reviewed the study and wrote the manuscript. FP and YZ prepared the manuscript with input from other authors.

## Supplementary Material

Supplemental data

Unedited blot and gel images

Supporting data values

## Version 1 04/08/2025

In-Press Preview

## Version 2 06/02/2025

Electronic publication
