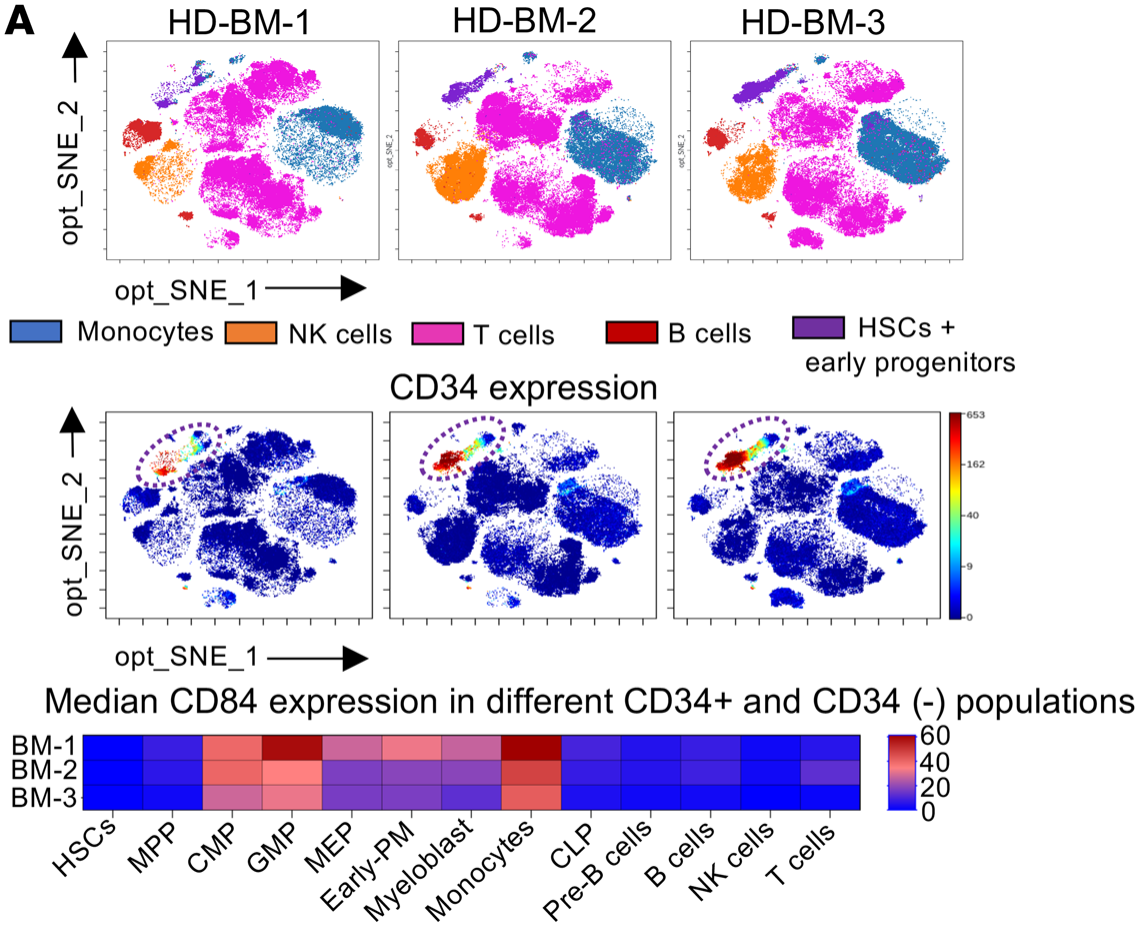# Corrigendum to Identification of CD84 as a potent survival factor in acute myeloid leukemia

**DOI:** 10.1172/JCI196910

**Published:** 2025-08-01

**Authors:** Yinghui Zhu, Mariam Murtadha, Miaomiao Liu, Enrico Caserta, Ottavio Napolitano, Le Xuan Truong Nguyen, Huafeng Wang, Milad Moloudizargari, Lokesh Nigam, Theophilus Tandoh, Xuemei Wang, Alex Pozhitkov, Rui Su, Xiangjie Lin, Marc Denisse Estepa, Raju Pillai, Joo Song, James F. Sanchez, Yu-Hsuan Fu, Lianjun Zhang, Man Li, Bin Zhang, Ling Li, Ya-Huei Kuo, Steven Rosen, Guido Marcucci, John C. Williams, Flavia Pichiorri

Original citation: *J Clin Invest*. 2025;135(11):e176818. https://doi.org/10.1172/JCI176818

Citation for this corrigendum: *J Clin Invest*. 2025;135(15):e196910. https://doi.org/10.1172/JCI196910

In Figure 1A of the original article, there was an error in the order of the CyTOF images in the top row. The correct figure, based on the original source data, is provided below. The HTML and PDF versions of the paper have been updated.

The authors regret the error.

## Figures and Tables

**Figure 1A:**