# Supplementary material for: Identification of CD84 as a potent survival factor in acute myeloid leukemia
Source: J Clin Invest. 2025 Apr 8;135(11):e176818. doi: 10.1172/JCI176818 (PMC12126229; doi:10.1172/JCI176818)
Supplement: Supplemental data [file jci-135-176818-s329.pdf]

Supplementary Information

Supplementary Table 1- Mass cytometry panels

Mass cytometry panel for AML patients

Antibody	Clone	Tag	Cat. Number
**ANTI-FITC (CD38 ME)**	FIT-22	160Gd	3160011C
**CD86/B7.2**	IT2.2	156Gd	3156008B
**CD45**	HI30	89Y	3089003C
**CD80/B7-1**	2D10.4	161Dy	3161023C
**CD127/IL.7Ra**	A019D5	143Nd	3143012B
**CD3**	UCHT1	141Pr	3141019C
**CD19**	HIB19	142Nd	3142001C
**CD69**	FN50	144Nd	3144018C
**CD25**	2A3	149Sm	3149010C
**CD64**	10.1	146Nd	3146006C
**CD33**	WM53	163Dy	3163023C
**CD15/SSEA-1**	W6D3	172Yb	3172021C
**HLA-DR**	L243	173Yb	3173005C
**CD45RA**	HI100	170Er	3170010C
**CD279/PD-1**	EH12.2H7	155Gd	3155009C
**CD14**	M5E2	151Eu	3151009C
**CD56**	NCAM 16.2	176Yb	3176008C
**CD223/LAG-3**	11C3C65	175Lu	3175033C
**CD95/FAS**	DX2	152Sm	3152017C
**CD269 (BCMA)**	19F2	174Yb	Conj (357502)
**CD34**	581	166Er	31660012C
**CD28**	CD28.2	169Tm	Conj (302937)
**CD123**	6H6	168Er	Conj (306002)
**CD13**	WM15	147Sm	3147014B
**gammadelta2 (TCR Vdelta2)**	B6	150Nd	Conj (331402)
**CD163**	GHI/61	165Ho	3165017C
**CD16**	3G8	148Nd	3148004C
**CD90**	5E10	171Yb	328129
**CD70**	113-16	153Eu	Conj (355101)
**CD11b**	ICRF44	164Dy	Conj. (301302)
**CD4**	SK3	111Cd	Conj (344625)
**CD8**	SK1	113Cd	Conj (344727)
**CD274/PDL1**	29E.2A3	159Tb	3159029C
**CD47**	CC2C6	209Bi	3209004C
**CD27**	L128	158Gd	3158010C
**CD197/CCR7**	G043H7	167Er	3167009C
**CD117**	104D2	145Nd	313202
**CD84**	CD84.1.21	154Sm	3154013B

**CD20**	2H7	162Dy	Conj (302343)

Mass cytometry panel for healthy donor bone marrow to identify CD84 expression in HSCs and progenitors

Antibody	Clone	Tag	Cat. Number
**ANTI-FITC (CD38 ME)**	FIT-22	160Gd	3160011C
**CD10**	HI10a	156Gd	3156001B
**CD45**	HI30	89Y	3089003C
**CD80/B7-1**	2D10.4	161Dy	3161023C
**CD123**	6H6	143Nd	3143014B
**CD3**	UCHT1	141Pr	3141019C
**CD19**	HIB19	142Nd	3142001C
**CD69**	FN50	144Nd	3144018C
**CD25**	2A3	149Sm	3149010C
**CD64**	10.1	146Nd	3146006C
**CD33**	WM53	163Dy	3163023C
**CD15/SSEA-1**	W6D3	172Yb	3172021C
**HLA-DR**	L243	173Yb	3173005C
**CD45RA**	HI100	170Er	3170010C
**CD279/PD-1**	EH12.2H7	155Gd	3155009C
**CD14**	M5E2	151Eu	3151009C
**CD56**	NCAM 16.2	176Yb	3176008C
**CD223/LAG-3**	11C3C65	175Lu	3175033C
**CD95/FAS**	DX2	152Sm	3152017C
**CD269 (BCMA)**	19F2	174Yb	Conj (357502)
**CD34**	58I	166Er	31660012C
**CD28**	CD28.2	169Tm	Conj (302937)
**CD13**	WM15	147Sm	3147014B
**gammadelta2 (TCR Vdelta2)**	B6	150Nd	Conj (331402)
**CD163**	GHI/61	165Ho	3165017C
**CD16**	3G8	148Nd	3148004C
**CD90**	5E10	171Yb	328129
**CD70**	113-16	153Eu	Conj (355101)
**CD11b**	ICRF44	164Dy	Conj. (301302)
**CD4**	SK3	111Cd	Conj (344625)
**CD8**	SK1	113Cd	Conj (344727)
**CD274/PDL1**	29E.2A3	159Tb	3159029C
**CD47**	CC2C6	209Bi	3209004C
**CD135**	BV10A4H2	158Gd	3158019B
**CD197/CCR7**	G043H7	167Er	3167009C
**CD117**	104D2	145Nd	313202
**CD84**	CD84.1.21	154Sm	3154013B

**CD20**	2H7	162Dy	Conj (302343)

“Conj” indicates in-house conjugated.

Supplementary Table 2-AML patient information

ID	Type	Status	Gene mutation	Cytogenetic	%Blast	Used in Figure
**AML #1**	PB	Refractory	FLT3-ITD, NRAS, PTPN11, WT1	CK	98	Figures 1,5,6
**AML #2**	PB	Refractory	KIT, NRAS, TP53	CK	64	Figures 1
**AML #3**	PB	Newly Diagnosed	IDH1, NPM1	NK	61	Figure 1,2,3,4, 5
**AML #4**	PB	Newly Diagnosed	-	CK	/	Figure 1,4
**AML #5**	PB	Refractory	ASXL1, CDK6, NRAS, WT1	CK	78	Figure 1
**AML #6**	PB	Relapsed	BCOR, CDK6, DNMT3A, IDH2, NRAS	CK	64	Figure 1
**AML #7**	PB	Newly Diagnosed	TP53	CK	25	Figure 1,5
**AML #8**	PB	Relapsed	ASXL1, CBL, FLT3, PHF6, TET2	CK	49	Figure 1,2
**AML #9**	PB	Newly Diagnosed	TP53	CK	13	Figure 1
**AML #10**	PB	Newly Diagnosed	-	CK	/	Figure 1
**AML #11**	PB	Relapsed	BCOR, EP300, NRAS, KMT2A-MLLT10, TBLXR1-TP63	CK	95	Figure 1
**AML #12**	PB	Refractory	CEBPA	CK	99	Figure 1
**AML #13**	PB	Relapsed	TET2, KRAS, DNMT3A	CK	34	Figures 1,4,5,6
**AML #14**	BM	Newly diagnosed	NRAS, KIT	NK	63	Figure 1
**AML #15**	BM	Newly diagnosed	TET2, CEBPA, KDM5A	NK	46	Figure 1
**AML #16**	BM	Newly diagnosed	/	/	/	Figure 1
**AML #17**	BM	Newly diagnosed	C-KIT	CK	73	Figure 1
**AML #18**	BM	Newly diagnosed	/	/	/	Figure 1
**AML #19**	BM	Newly diagnosed	CROCC, KIT, KDM6A		42	Figure 1
**AML #20**	BM	Newly diagnosed	/	NK	83	Figure 1
**AML #21**	BM	Newly diagnosed	DNMT3A	NK	48	Figure 1

**AML #22**	BM	Newly diagnosed	IDH1, FLT3-ITD, NPM1, TET2	NK	64	Figure 1
**AML #23**	BM	Newly diagnosed	/	/	87	Figure 1
**AML #24**	BM	Newly diagnosed	FLT3-ITD	NK	28	Figure 1
**AML #25**	BM	Newly diagnosed	FLT3-ITD, IDH1, BCOR, DNMT3A	NK	96	Figure 1
**AML #26**	BM	Newly diagnosed	/	NK	69	Figure 1
**AML #27**	BM	Newly diagnosed	/	NK	35	Figure 1
**AML #28**	BM	Newly diagnosed	/	NK	31	Figure 1
**AML #29**	BM	Newly diagnosed	/	CK	41	Figure 1
**AML #30**	BM	Newly diagnosed	NPM1	CK	24	Figure 1
**AML #31**	BM	Newly diagnosed	/	NK	91	Figure 1
**AML #32**	BM	Relapsed	/	CK	50	Figure 1
**AML #33**	BM	Relapsed	/	CK	70	Figure 1
**AML #34**	BM	Relapsed	/	CK	95	Figure 1
**AML #35**	BM	Relapsed	/	CK	95	Figure 1
**AML #36**	BM	Relapsed	/	CK	/	Figure S2
**AML #37**	BM	Relapsed	/	CK	/	Figure S2
**AML #38**	BM	Relapsed	/	CK	/	Figure S2
**AML #39**	BM	Newly diagnosed	DNMT3A, IDH	NK	/	Figure 2
**AML #40**	PB	Relapsed	ASXL2, FLT3-ITD, RUNX1, SP3B1	CK	86	Figure 4

NK: Normal Karyotype; CK: Complex Karyotype

Supplementary Table 3-The percentage of CD84 positive population in AML cohorts (n=31)

**CD84 positive %**	**Newly diagnosed**	**%**	**Relapsed/ Refractory**	**%**	**Total**	**%**
$CD45^{dim}$ (Blast)						
>70%	9	39.13%	7	87.5%	16	**51.6%**
40%-70%	7	30.43%	-	-	7	22.6%
<40%	7	30.43%	1	12.5%	8	25.8%
>50%	14	60.87%	-	-	21	67.7%
$CD34^{+}CD38^{+}$						
>70%	10	43.48%	7	87.5%	16	**53.33%**
50%-70%	3	13.04%	-	-	3	9.7%
<40%	10	43.48%	1	12.5%	11	35.5%
>50%	13	56.52%	-	-	20	64.5%

Supplemental Figure

Supplementary Figure 1

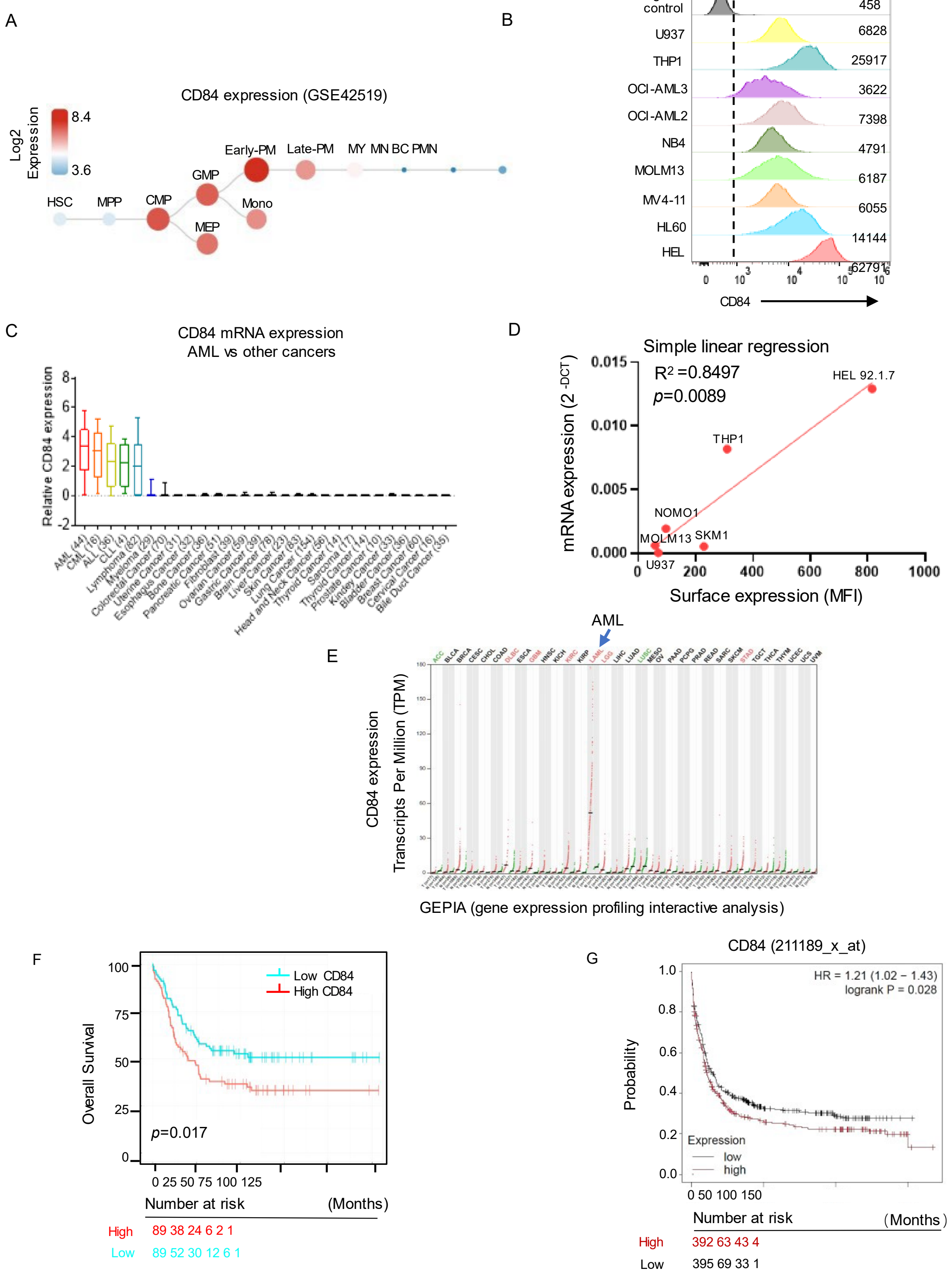

Supplementary Figure 1. CD84 is highly expressed in AML cell lines and is significantly correlated with poor survival in AML patients. (**A**) Interactive hierarchical tree that shows CD84 expression in human hemopoietic system. The color in the nodes represents the median expression of the queried gene, the size of the nodes represents the expression proportion. The analyzed was performed in BloodSpot database (GSE42519). (**B**) Histogram showing CD84 surface protein expression in AML cell lines (n=9) as analyzed by flow cytometry, highlighting that CD84 is highly expressed in AML cell lines. PE anti-human CD84 (clone CD84.1.21; Biolegend) was used (1 µl/test). (**C**) Bar chart showing relative CD84 expression levels among different human cells in the Cancer Cell Line Encyclopedia database. Graph are presented as mean ± SEM. (**D**) Dot plot showing simple linear regression analysis correlating CD84 mRNA and protein expression in different AML cell lines. CD84 mRNA was assessed with qRT-PCR and protein expression was determined with surface flow cytometry analysis using PE anti-human CD84 (clone CD84.1.21; Biolegend) was used (1 µl/test). (**E**) The CD84 expression profile across various kinds of tumor samples and paired normal tissues. Each dots represent the expression of tumor (red) and normal tissue (green). (**F**) Kaplan-Meier survival analysis of AML patients in the GSE10358 dataset. The patients were divided into two groups based on CD84 levels using the median as the cut-off. Statistical significance was assessed by log-rank test. (**G**) Kaplan-Meier survival analysis of AML patients in the GSE1159, GSE12417, GSE37642, GSE6891, and GSE8970 datasets. The patients were divided into two groups based on CD84 levels using the median as the cut-off. Statistical significance was assessed by log-rank test.

Supplementary Figure 2

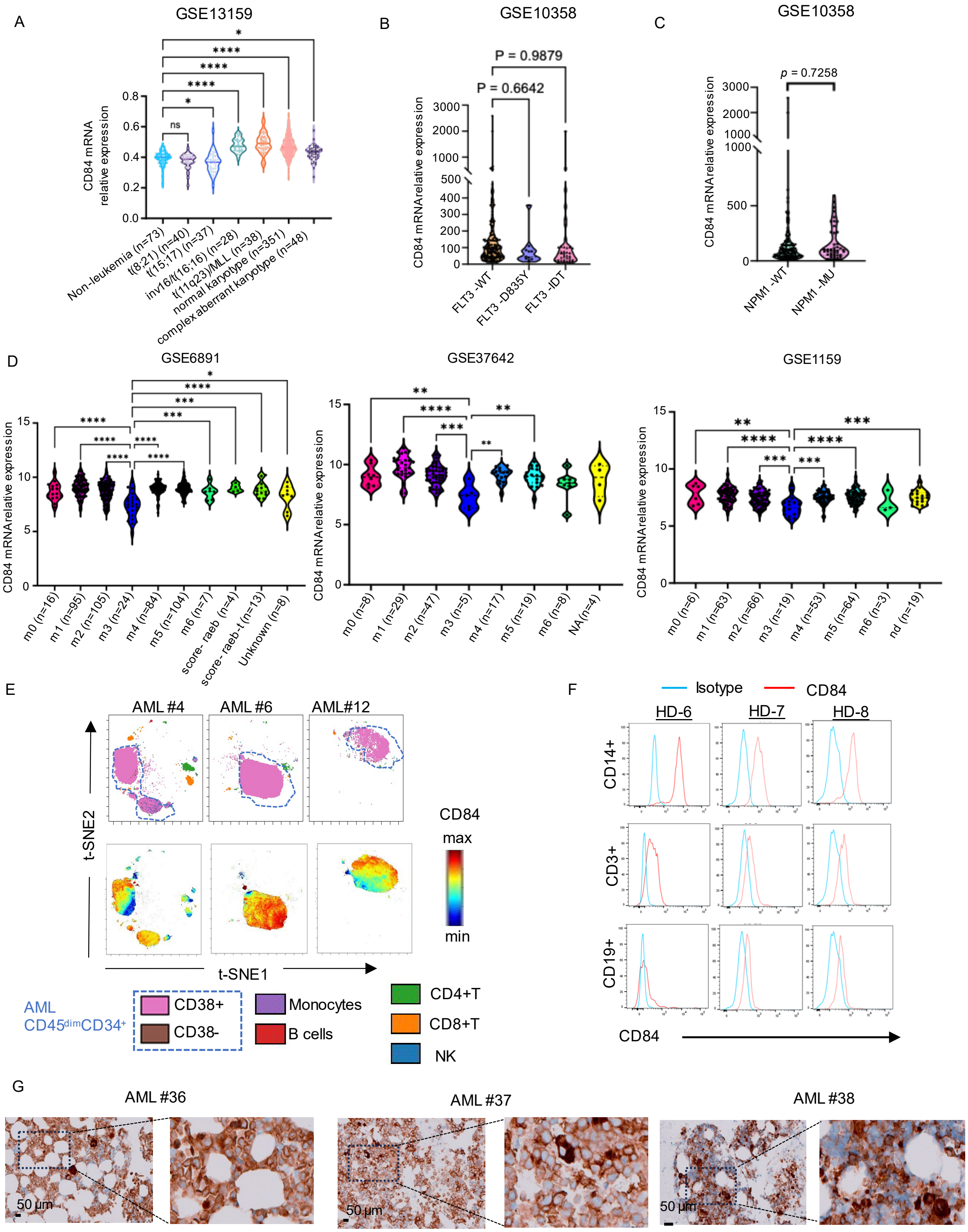

Supplementary Figure 2. CD84 is highly expressed in different AML subtypes. (A) The mRNA expression of CD84 in AML patients with various cytogenetic abnormalities (GSE13159). Violin plot are presented as mean ± SEM. Statistical significance was assessed by one-way ANOVA. **(B)** The mRNA expression of CD84 in AML patients with wildtype or common FLT3 mutations (GSE10358). Violin plot are presented as mean ± SEM. Statistical significance was assessed by one-way ANOVA. (**C**) The mRNA expression of CD84 in AML patients with wildtype or common NPM1 mutations (GSE10358). Violin plot are presented as mean ± SEM. Statistical significance was assessed by two tailed unpaired t test. (**D**) The mRNA expression of CD84 in AML patients across different FAB subtypes in GSE6891, GSE37642, and GSE1159 using R2 database. Violin plot are presented as mean ± SEM. Statistical significance was assessed by one-way ANOVA. (**E**) Bone marrow cells from three individual AML patients were subjected to CyTOF immunophenotyping comprising 39 surface markers tailored to detect different immune subsets. Analysis was performed with Cytobank© platform. (**F**) Histogram showing representative flow cytometry profiles of CD84 expression in different cell populations (CD14+/CD3+/CD19+) in PBMCs from healthy donors. The analysis was conducted in three independent healthy donors. (**G**) Representative images of immunohistochemical staining of CD84 in three AML bone marrow. Scale bar: 50 μm. The analysis was conducted in three independent AML donor biopsies. *n.s.*, not significant, $*p < 0.05$, $**p < 0.01$, $***p < 0.001$, and **** $p<0.0001$.

Supplementary Figure 3

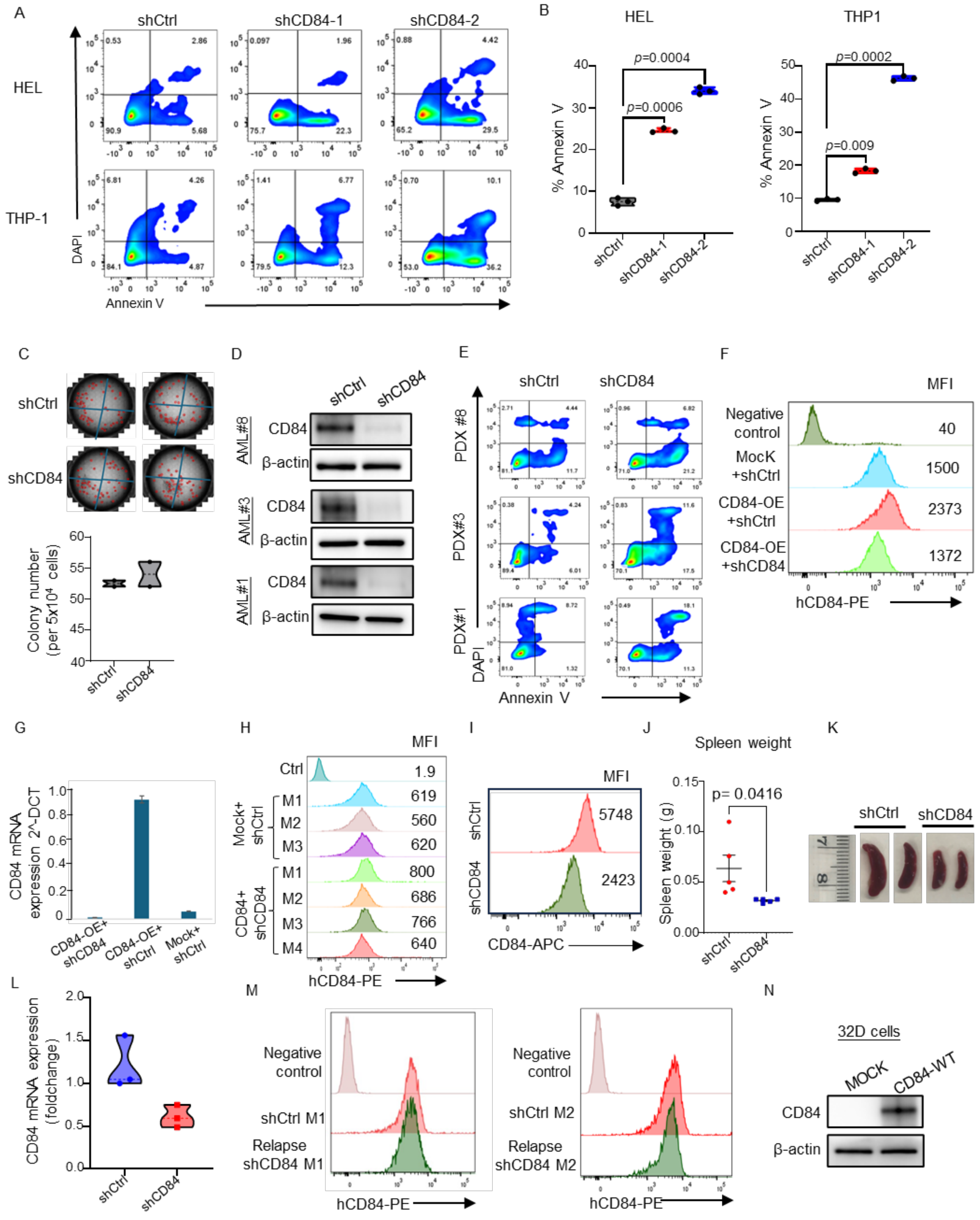

Supplementary Figure 3. Loss of CD84 reduces AML survival *in vitro* and *in vivo*. (**A-B**) Representative flow cytometry and violin chart showing apoptosis levels indicated by Annexin V-APC/DAPI in HEL cells and THP-1 cells transduced with shCtrl or shCD84 lentiviral vectors. Violin plot in Figure B are presented as mean ± SEM and are representative of 3 independent experiments. Statistical significance was assessed by one way ANOVA. (**C**) Representative image and violin plot showing the colony formation ability of one healthy donor derived CD34+ cells transduced with shCtrl or shCD84. Data are presented as mean ± SEM and are representative of 2 independentdonors. (**D**) Representative western blot of the indicated proteins in three independent AML primary cells transduced with shRNA against CD84 (shCD84) or shCtrl. (**E**) Representative flow cytometry data showing apoptosis induced by CD84 knockdown in three independent AML primary cells. (**F**) Histogram showing CD84 surface expression in mock/shCtrl, CD84-OE/shCtrl or CD84-OE/shCD84–transduced THP1-luciferase cells. (**G)** Bar chart shown CD84 mRNA relative expression in THP1 cells transduced with mock/shCtrl, CD84-OE/shCtrl or CD84-OE/shCD84. Data are presented as mean ± SEM and are representative of 3 independent experiments. (**H**) Histogram showing the escape from shCD84 knockdown in relapsed mice xenografted with CD84-OE/shCD84 transduced THP1-luciferase cells. The analysis is conducted in at least three individual mice per group. (**I)** Representative histogram showing CD84 expression in AML primary cells (AML #3) transduced with shCtrl or shCD84. **(J)** Violin plot shown the spleen weight of recipient mice xenografted with MLL-AF9 transduced with shCtrl or shCD84. Data are presented as mean ± SEM and are representative of 5 individual mice. Statistical significance was assessed by two tailed unpaired t test. **(K)** Representative images of spleens of recipient mice xenografted with MLL-AF9 transduced with shCtrl or shCD84. **(L)** Violin plot shows mRNA relative expression of CD84 in shCtrl or shCD84–transduced AML PDX-luciferase cells. Violin plot are presented as mean ± SEM and are representative of 3 independent experiments. **(M)** Histogram showing the escape from shCD84 knockdown in relapsed mice xenografted with shCD84 transduced THP1-luciferase cells. The analysis is conducted in two individual mice per group. (**N**) Western blot of the indicated proteins in 32D cells transduced with CD823-mock vector or CD823-CD84-WT. Data are representative of at least 2 independent experiments.

Supplementary Figure 4

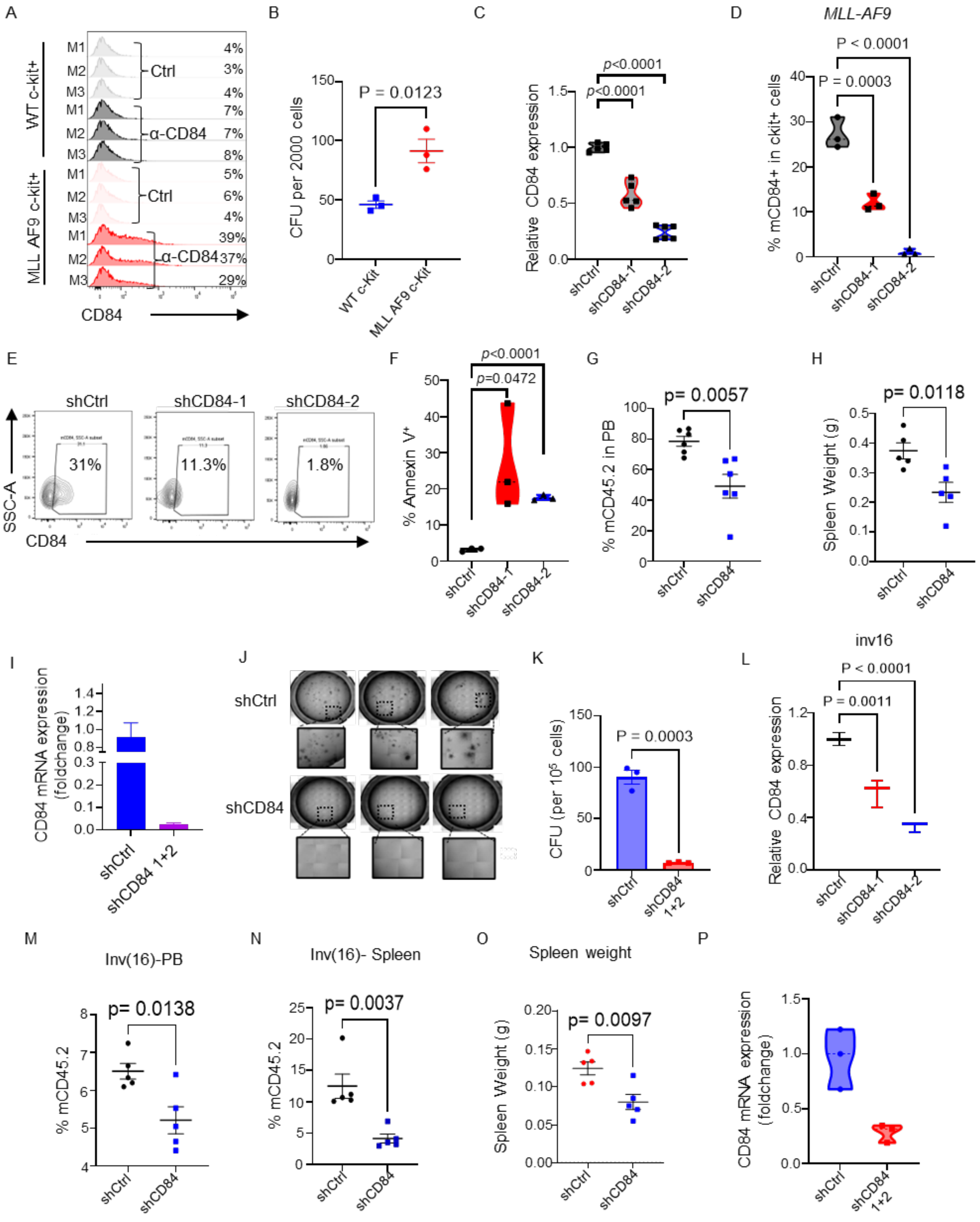

Supplementary Figure 4. CD84 is critical for sustaining AML in MLL-AF9 and inv(16) mouse model. (**A**) Histogram showing the positive ratio of CD84 in wildtype c-kit+ cells and MLL-AF9 c-kit cells. The analysis is conducted in three individual mice. (**B**) The scatter plot shows colony formation numbers after 7 days of culture in WT c-kit and MLL-AF9 group. Data are presented as mean ± SEM and are representative of 3 independentmice. Statistical significance was assessed by two tailed unpaired t test. (**C**) Violin chart showing relative mRNA expression of CD84 (normalized to GAPDH) in shCtrl, shCD84-1 or shCD84-2 transduced MLL-AF9 cells. Data are presented as mean ± SEM and are representative of 5 independent experiments. Statistical significance was assessed by one way ANOVA. (**D**) Violin chart showing the positive ratio of mouse CD84 in shCtrl, shCD84-1 or shCD84-2 transduced MLL-AF9 cells. Data are presented as mean ± SEM and are representative of 3 independentmice. Statistical significance was assessed by one way ANOVA. (**E**) Representative flow cytometry profile showing mouse CD84 expression in MLL-AF9 cell transfected with shCtrl, shCD84-1 or shCD84-2. (**F**) Violin chart showing the apoptosis ratio of MLL-AF9 AML cells transfected with shCtrl or shCD84. Data are presented as mean ± SEM and are representative of 3 mice. Statistical significance was assessed by one way ANOVA. (**G**) Scatter plots showing the percentage of donor cells (mouse CD45.2) engrafted in PB from shCtrl-MLL-AF9 or shCD84-MLL-AF9 transplanted mice (n=6 per group). Data are presented as mean ± SEM and are representative of 6 individual mice. Statistical significance was assessed by by two tailed unpaired t test. (**H**) Scatter plots showing spleen weight of recipient mice xenografted with shCtrl-MLL-AF9 or shCD84-MLL-AF9 (n=5 per group). Data are presented as mean ± SEM and are representative of 5 individual mice. Statistical significance was assessed by by two tailed unpaired t test. (**I**) Bar chart showing relative mRNA expression of CD84 in shCtrl, shCD84-1+2 transduced MLL-AF9 cells. Data are presented as mean ± SEM and are representative of 3 independent experiments. (**J**) Representative colony images of MLL-AF9 cells transduced with shCtrl or shCD84-1+2. Data are representative of 3 independent experiments. (**K**) The bar graph shows colony formation numbers of MLL-AF9 transduced with shCtrl or shCD84-1+2 after 7 days of culture. Data are presented as mean ± SEM and are representative of 3 independent experiments. Statistical significance was assessed by two tailed unpaired t test. (**L**) Box chart showing relative

mRNA expression of CD84 (normalized to GAPDH) in shCtrl, shCD84-1 or shCD84-2 transduced inv(16)-AML cells. Data are presented as mean ± SEM and are representative of 3 independent experiments. Statistical significance was assessed by one way ANOVA. (**M**) Scatter plot showing the percentage of donor cells (mouse CD45.2) engrafted in PB from shCtrl-inv(16)-AML or shCD84-inv(16)-AML transplanted mice (CD45.1 mice). Data are presented as mean ± SEM and are representative of 5 individual mice. Statistical significance was assessed by by two tailed unpaired t test. (**N**) Scatter plot showing the percentage of donor cells (mouse CD45.2) engrafted in spleen from shCtrl-inv(16)-AML or shCD84-inv(16)-AML transplanted mice (CD45.1 mice). Data are presented as mean ± SEM and are representative of 5 individual mice. Statistical significance was assessed by by two tailed unpaired t test. (**O**) Scatter plot showing spleen weight of recipient mice xenografted with shCtrl-inv(16)-AML or shCD84-inv(16)-AML (n=5/group). Data are presented as mean ± SEM and are representative of 5 individual mice. Statistical significance was assessed by by two tailed unpaired t test. (**P)** Violin chart showing relative mRNA expression of CD84 in shCtrl, shCD84-1+2 transduced inv(16) AML cells. Data are presented as mean ± SEM and are representative of 3independent experiments.

Supplementary Figure 5

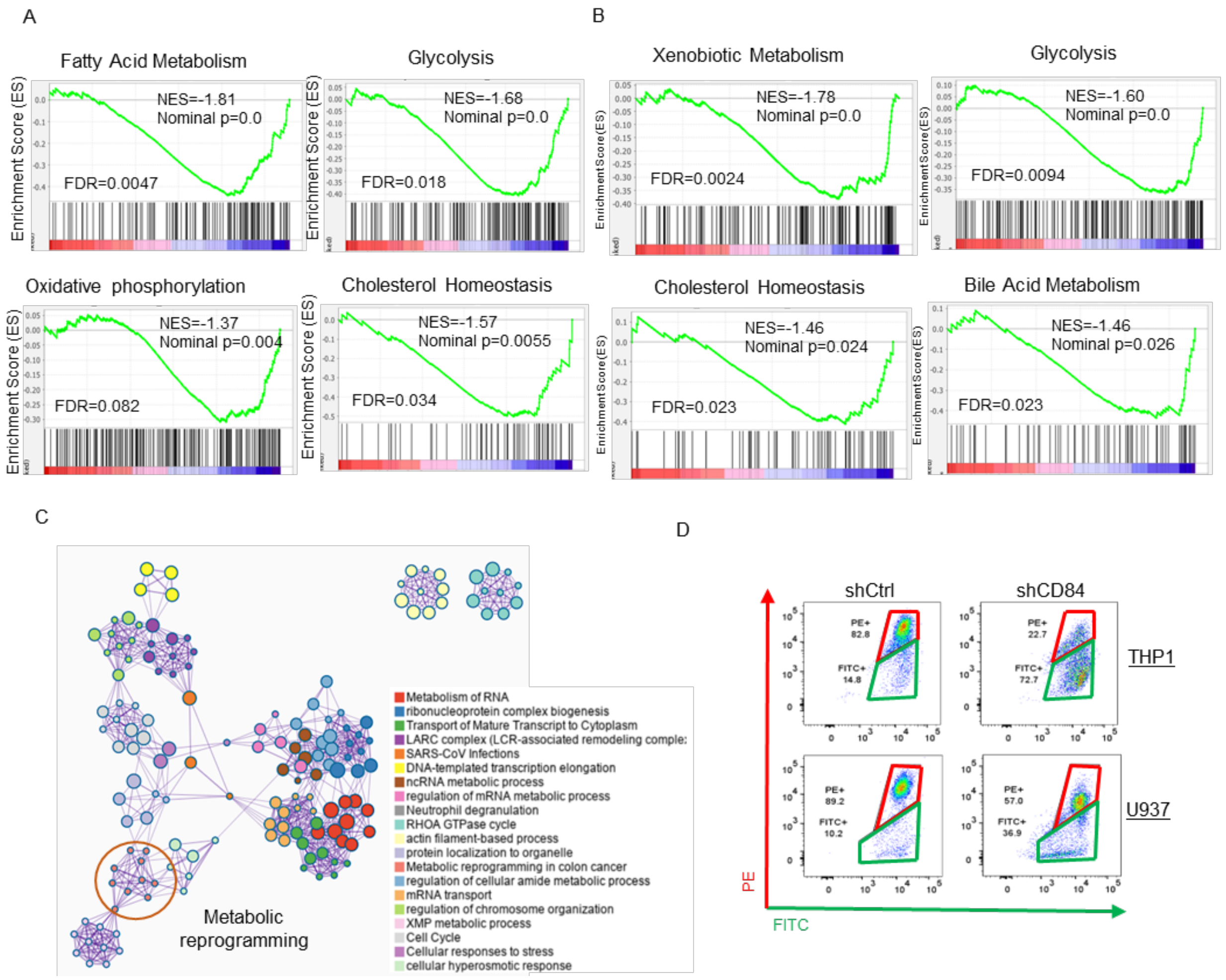

Supplementary Figure 5. RNA-seq analysis indicated that CD84 deletion might induced mitochondrial dysfunction. (**A-B**) Gene set enrichment analysis (GSEA) plots of several gene clusters that were down-regulated upon CD84 knockdown in HEL cells (**A**) and THP1 cells (**B**). (**C**) KEGG pathway analysis of 188 common DEGs upon CD84 knockdown in both HEL cells and THP1 cells. (**D**) Representative flow cytometry profiles of JC-1 stained THP1 cells and U937 cells, which were transduced with lentiviruses expressing shCtrl or shCD84. Red (PE): Green (FITC) represents the monomers to aggregated ratio. Data are are representative of 3 independent experiments.

Supplementary Figure 6

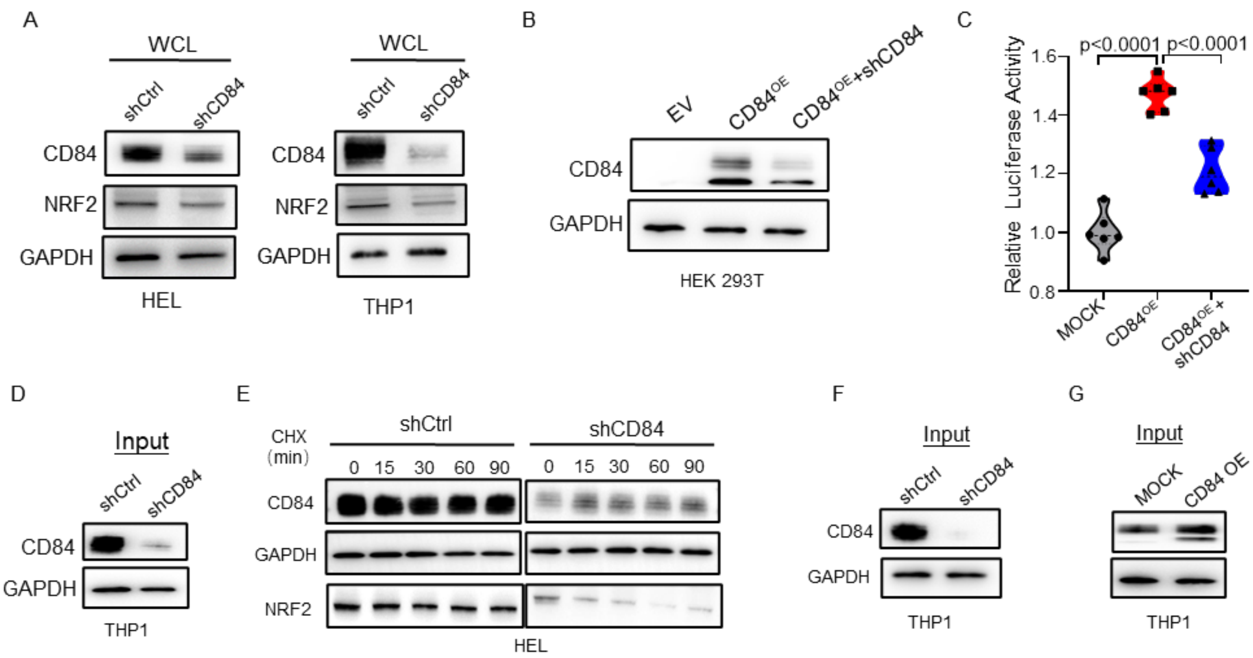

Supplementary Figure 6. CD84 regulates the transcriptional activity and protein stability of NRF2. (**A**) The western blot shows analysis of NRF2 expression in the whole lysate of HEL cells and THP1 cells infected with viruses expressing CD84 shRNA or shCtrl. Data are representative of at least 2 independent experiments. (**B-C**) HEK 293T cells stably expressing 3xFlag-CD84 were further infected with viruses expressing CD84 shRNA (targeting CDS) and then subjected to immunoblotting with anti-CD84 antibodies. ARE-regulated luciferase reporter gene activity, which was responsive to Nrf2, in HEK 293T cells was detected after shCD84 transfection. The relative activities of firefly luciferase were analyzed after normalized with the activities of luciferase from pRL-TK vector. The immunoblot shows CD84 and GAPDH expression in indicated cells, data are representative of at least 2 independent experiments (B). The bar chart shows the relative NRF2 transcriptional activity measured by luciferase signaling in the indicated cells, data are presented as mean ± SEM and are representative of three independent experiments. Statistical significance was assessed by one-way ANOVA (C). (**D**) Immunoblot analysis of CD84 in input samples of THP1 cell transfected with either shCtrl or shCD84 lentivirus corresponding to main Fig. 9B. Data are representative of at least 2 independent experiments. (**E**) The immunoblot shows the time course of indicated protein expression

after CHX treatment in HEL. Western blot analysis confirmed the presence of NRF2 at times after CHX treatment in control samples. GAPDH was used as a control. Data are representative of at least 2 independent experiments. (**F**) Immunoblot analysis of CD84 in input samples of THP1 cell transfected with either shCtrl or shCD84 lentivirus corresponding to main Fig. 9D. Data are representative of at least 2 independent experiments. (**G**) Immunoblot analysis of CD84 in input samples of THP1 cell transduced with either mock or CD84-OE lentivirus corresponding to main Fig. 9E. Data are representative of at least 2 independent experiments.
