# Supplementary material for: Identification of CD84 as a potent survival factor in acute myeloid leukemia
Source: J Clin Invest. 2025 Apr 8;135(11):e176818. doi: 10.1172/JCI176818 (PMC12126229; doi:10.1172/JCI176818)
Supplement: Unedited blot and gel images [file jci-135-176818-s330.pdf]

Figure 2A

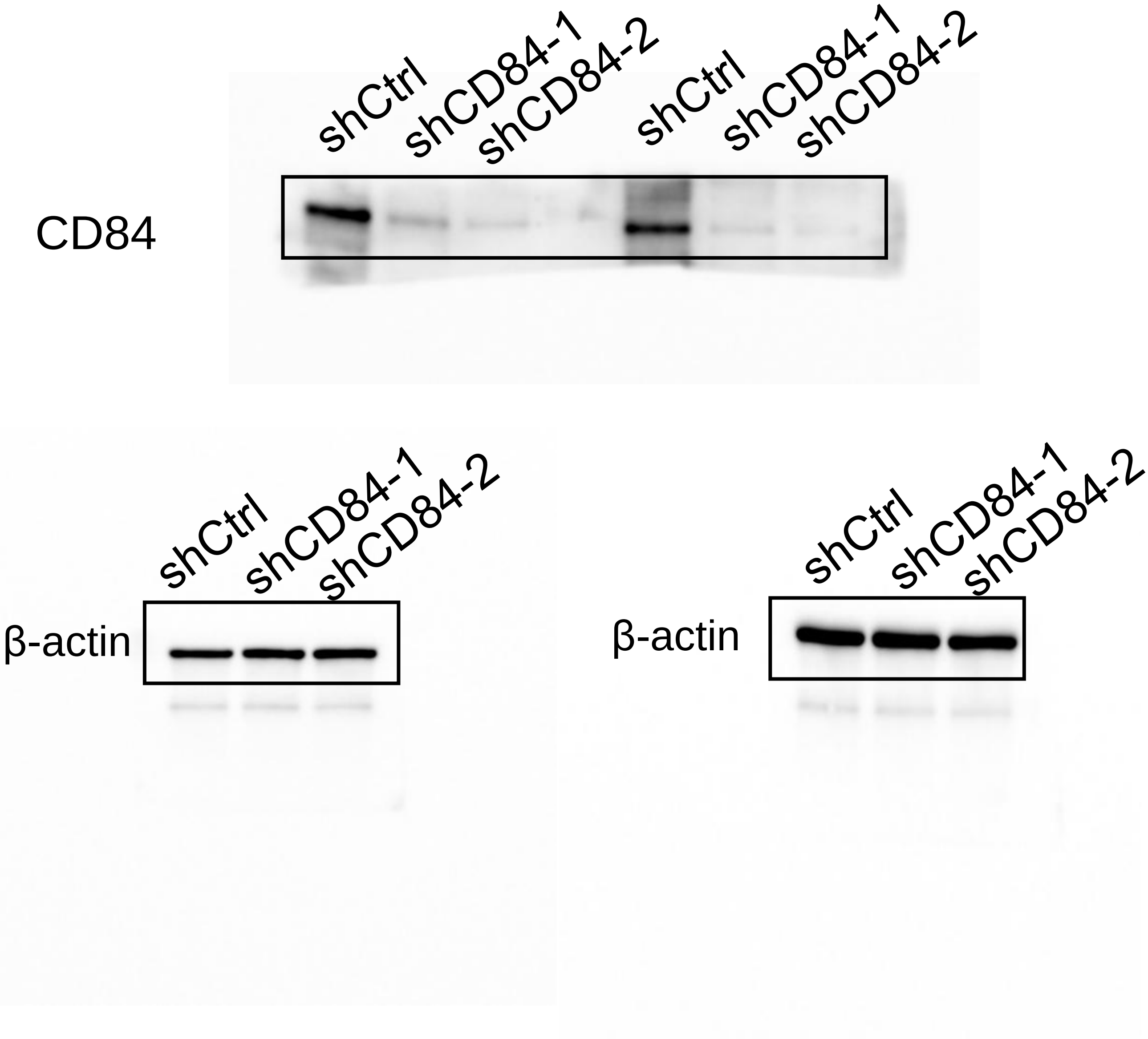
shCtrl
shCD84-1
shCD84-2
shCtrl
shCD84-1
shCD84-2
CD84
shCtrl
shCD84-1
shCD84-2
β-actin
shCtrl
shCD84-1
shCD84-2
β-actin

Figure 2A

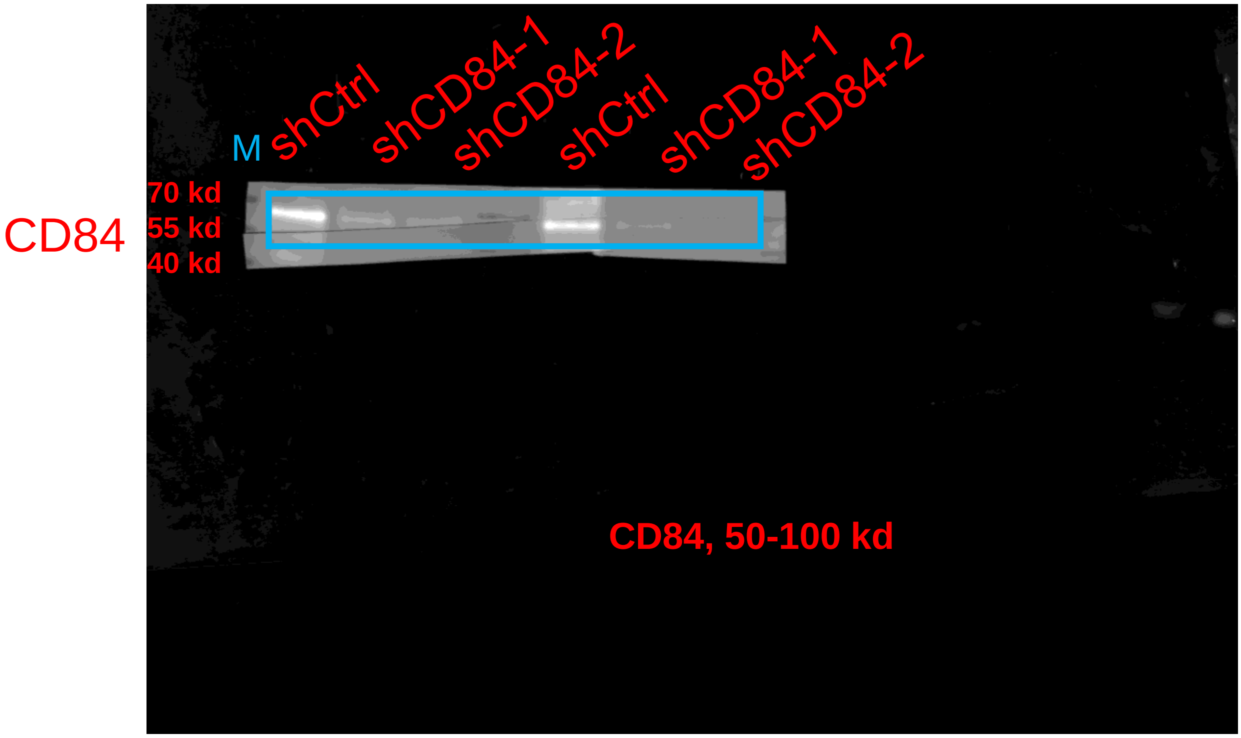

CD84
M
shCtrl
shCD84-1
shCD84-2
shCtrl
shCD84-1
shCD84-2
70 kd
55 kd
40 kd
CD84, 50-100 kd

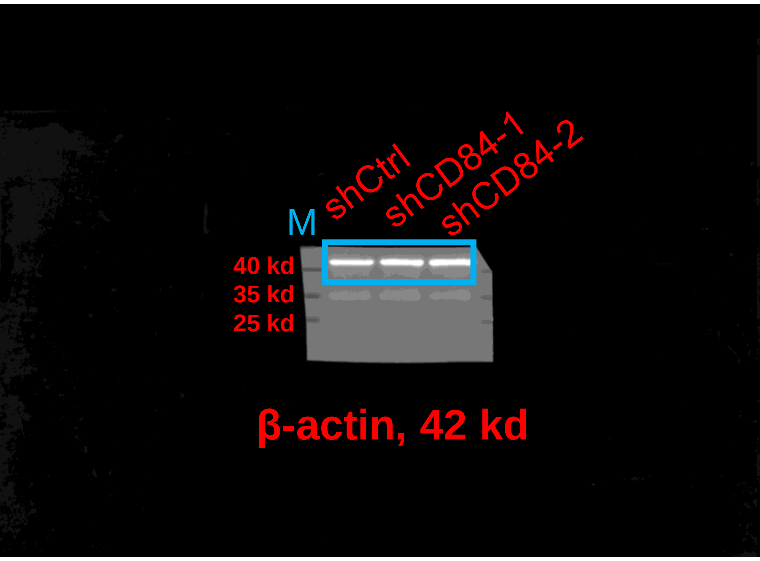

M
shCtrl
shCD84-1
shCD84-2
40 kd
35 kd
25 kd
β-actin, 42 kd

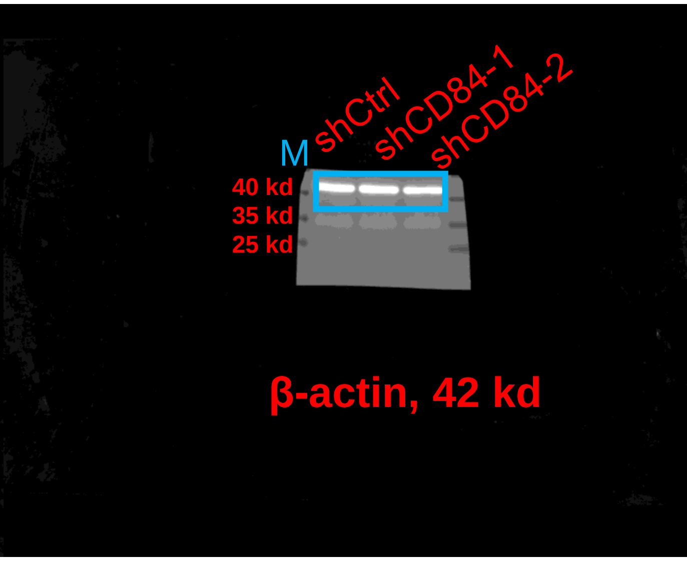

M
shCtrl
shCD84-1
shCD84-2
40 kd
35 kd
25 kd
β-actin, 42 kd

Figure S3D

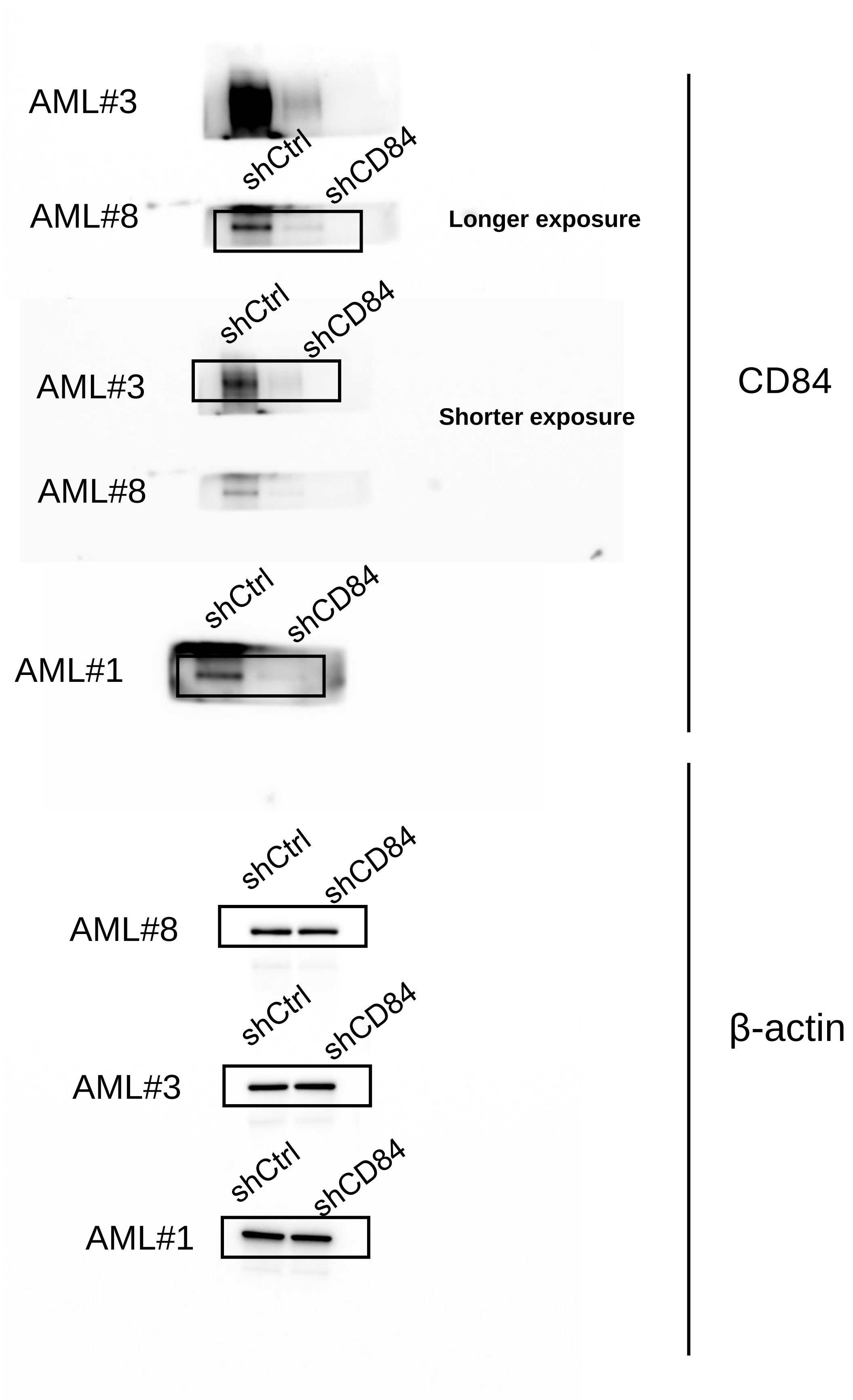
AML#3
shCtrl
shCD84
AML#8
Longer exposure
shCtrl
shCD84
AML#3
Shorter exposure
AML#8
shCtrl
shCD84
AML#1
CD84
shCtrl
shCD84
AML#8
shCtrl
shCD84
AML#3
shCtrl
shCD84
AML#1
β-actin

Figure S3D

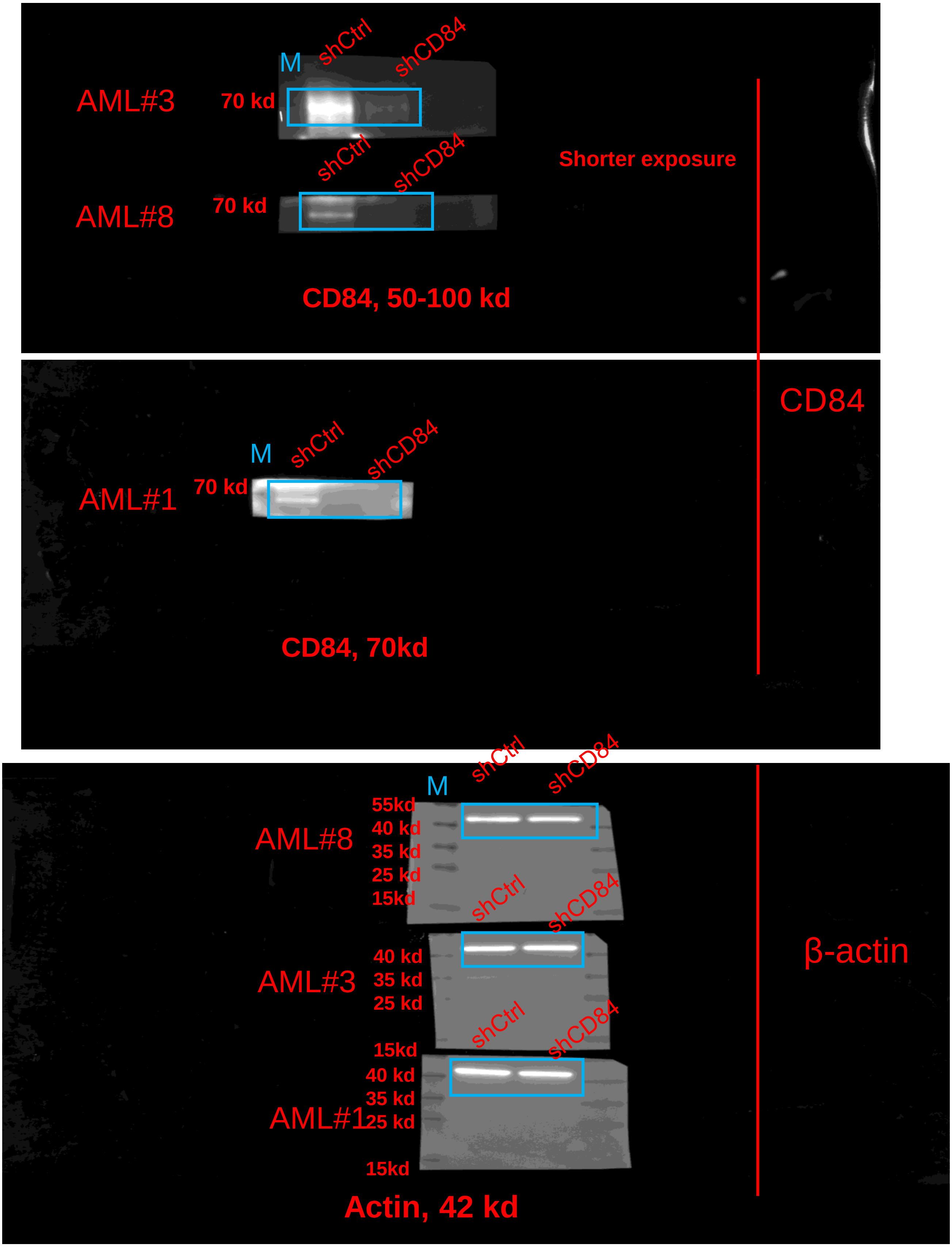

M
shCtrl
shCD84
AML#3
70 kd
shCtrl
shCD84
AML#8
70 kd
Shorter exposure
CD84, 50-100 kd
CD84
M
shCtrl
shCD84
AML#1
70 kd
CD84, 70kd
M
shCtrl
shCD84
AML#8
55kd
40 kd
35 kd
25 kd
15kd
shCtrl
shCD84
AML#3
40 kd
35 kd
25 kd
15kd
shCtrl
shCD84
AML#1
40 kd
35 kd
25 kd
15kd
β-actin
Actin, 42 kd

Figure S3N

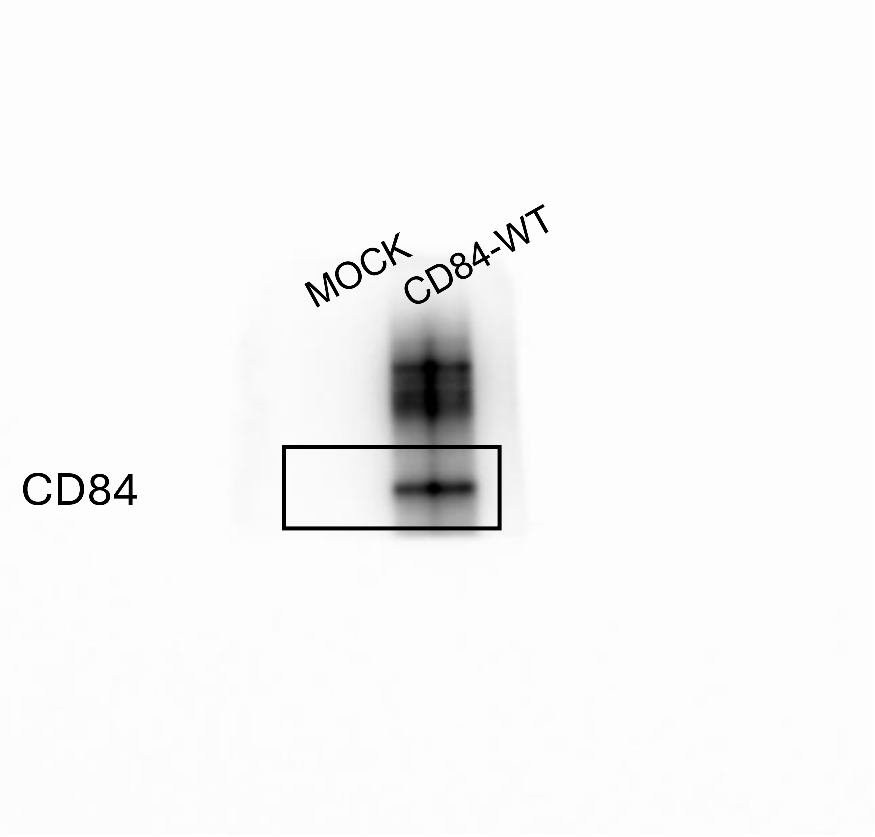

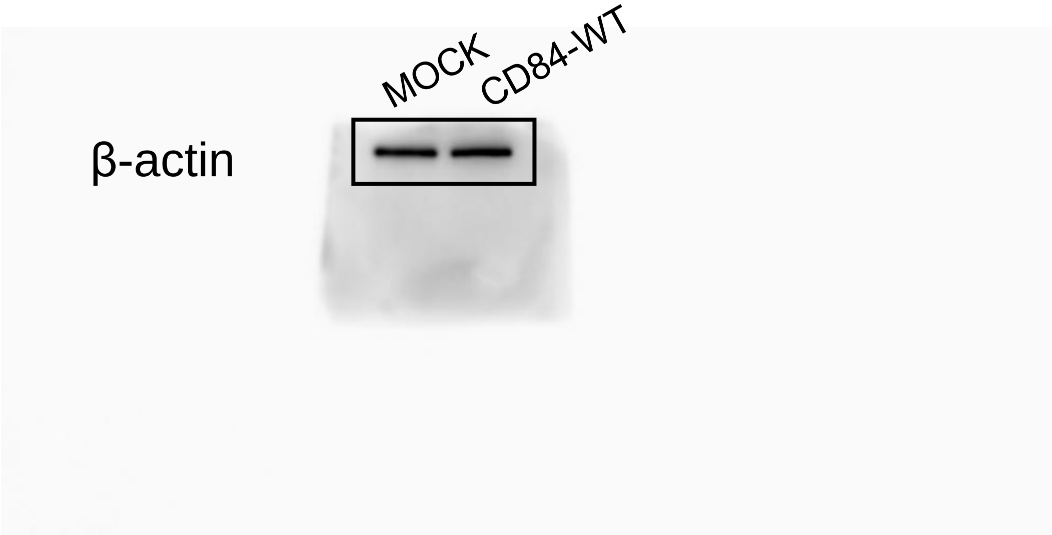

Figure S3N

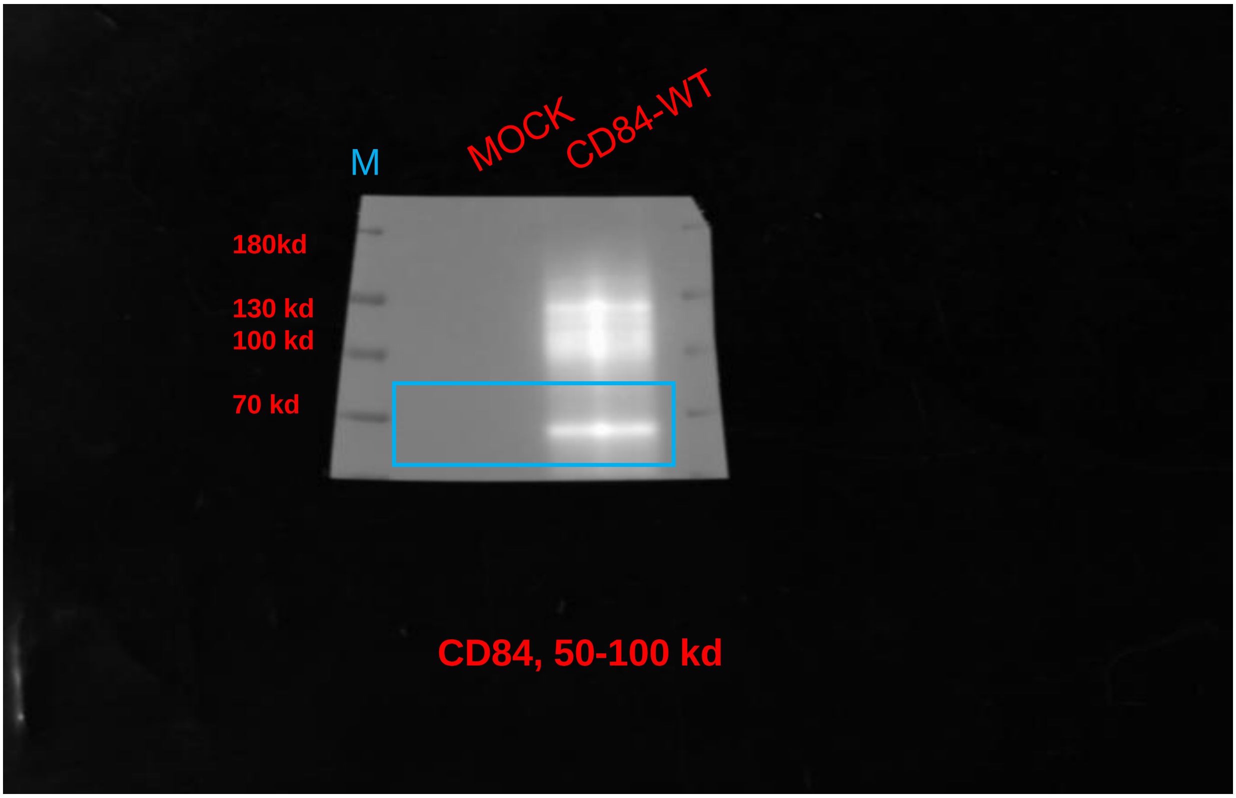
M
MOCK
CD84-WT
180kd
130 kd
100 kd
70 kd
CD84, 50-100 kd

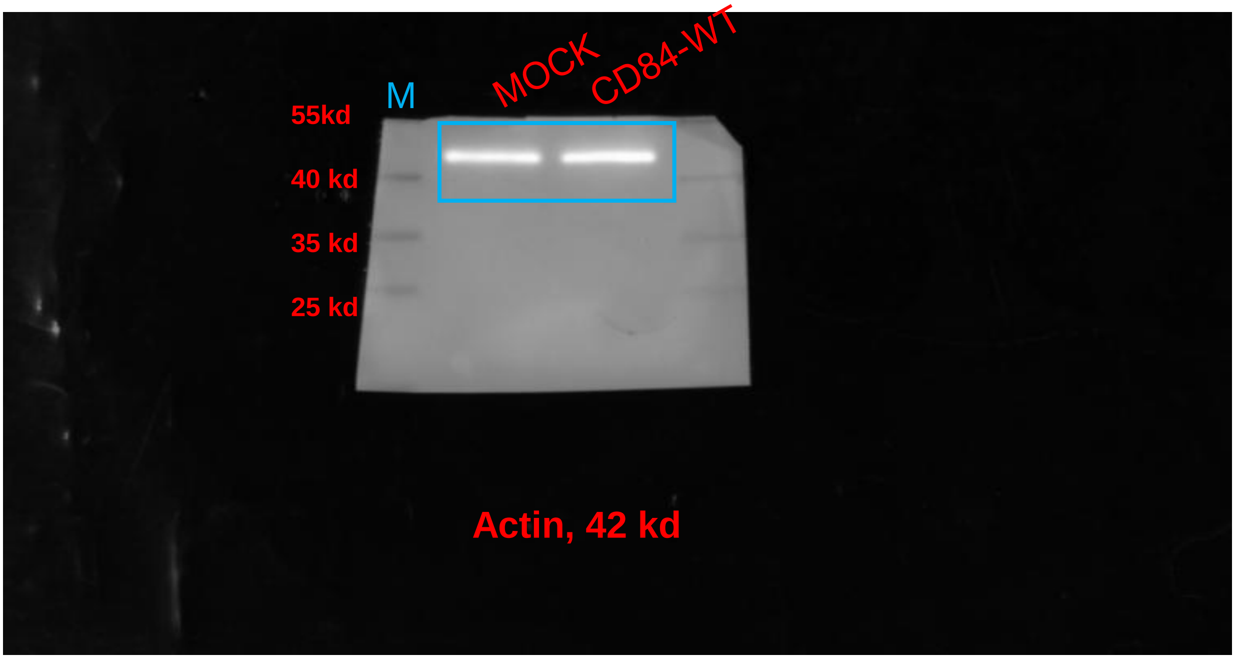
M
MOCK
CD84-WT
55kd
40 kd
35 kd
25 kd
Actin, 42 kd

Figure 7B

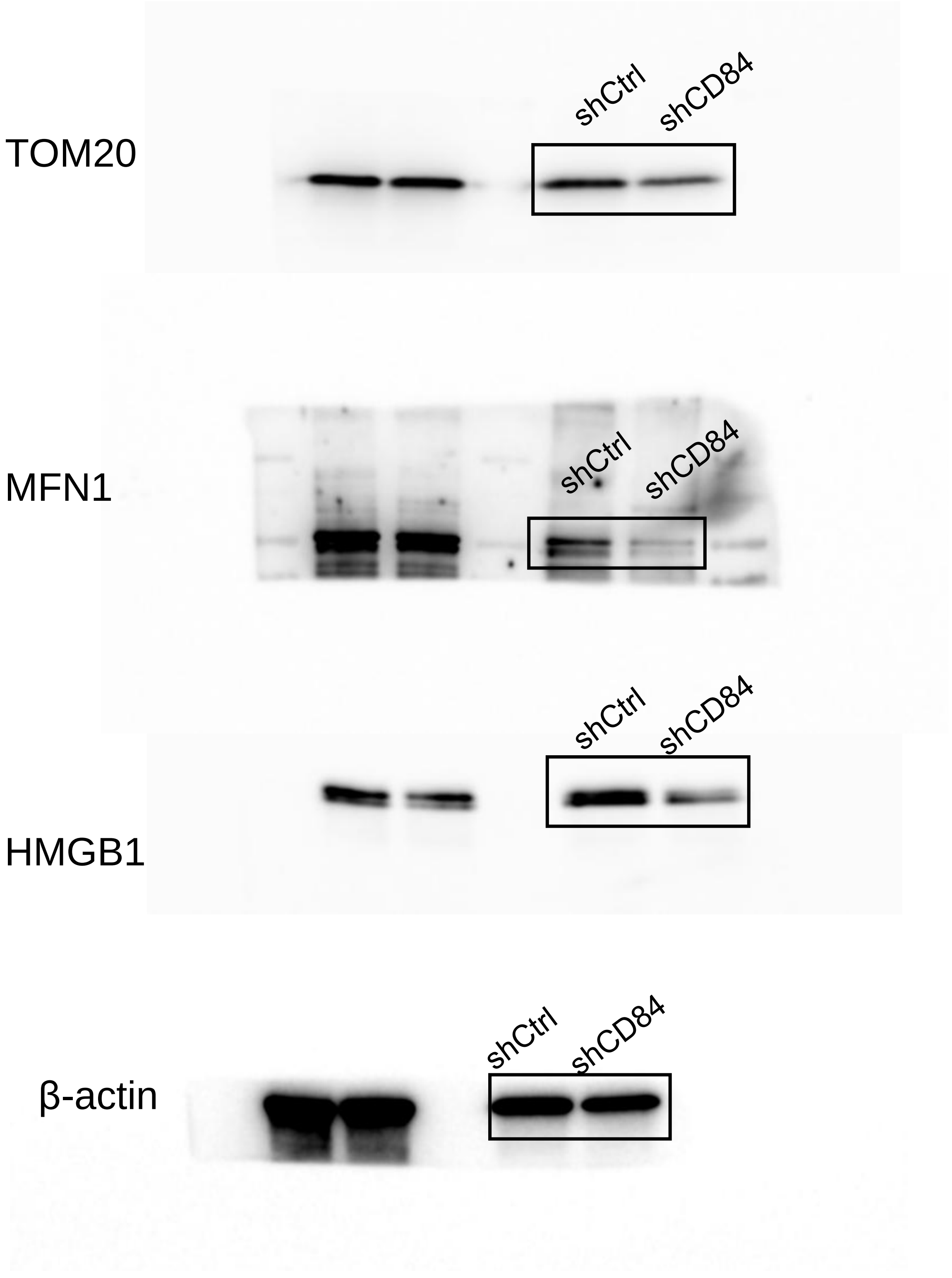
TOM20
shCtrl
shCD84
MFN1
shCtrl
shCD84
HMGB1
shCtrl
shCD84
β-actin
shCtrl
shCD84

Figure 7B

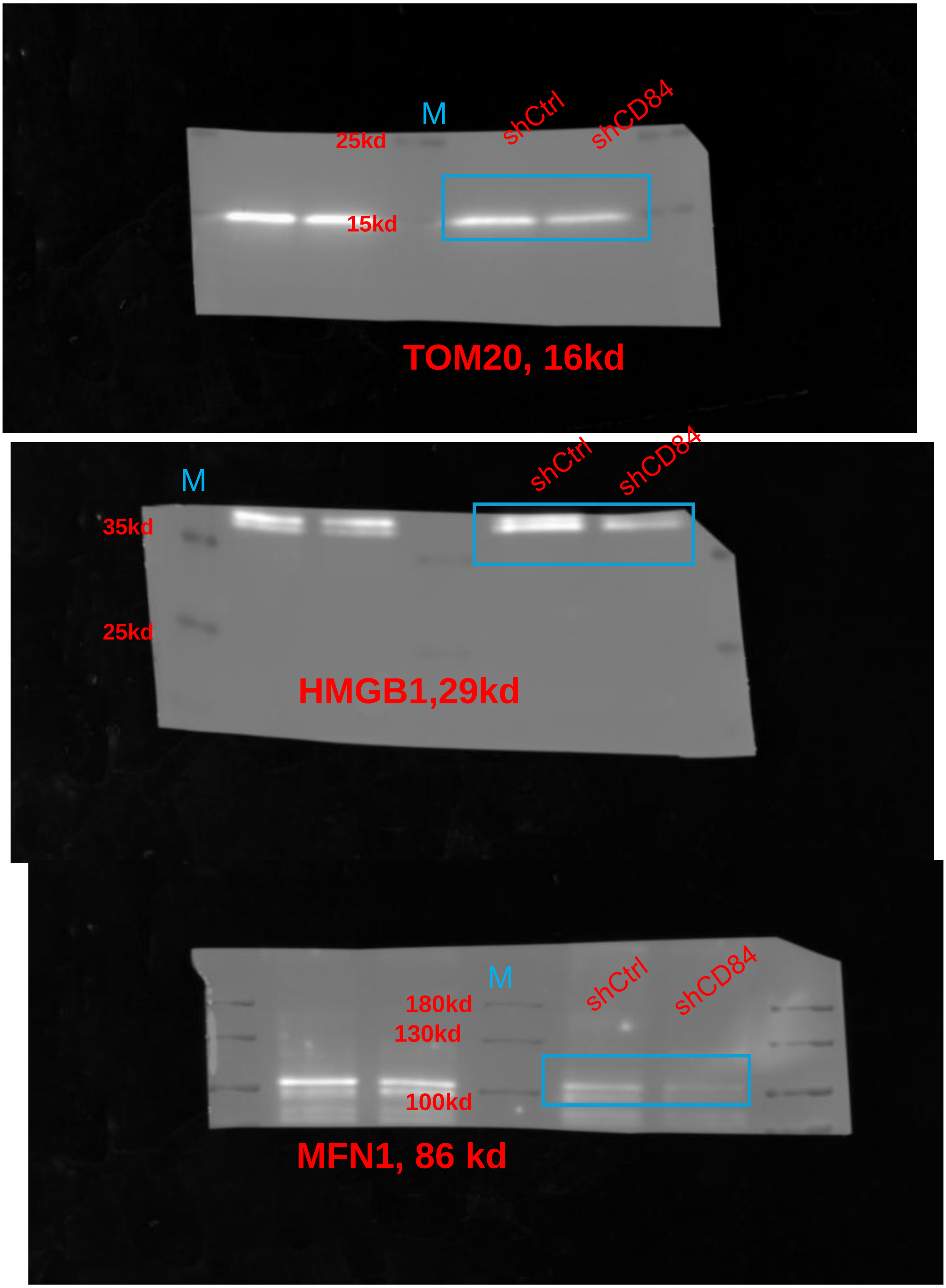
M
shCtrl
shCD84
25kd
15kd
TOM20, 16kd
M
shCtrl
shCD84
35kd
25kd
HMGB1,29kd
M
shCtrl
shCD84
180kd
130kd
100kd
MFN1, 86 kd

Figure 7F

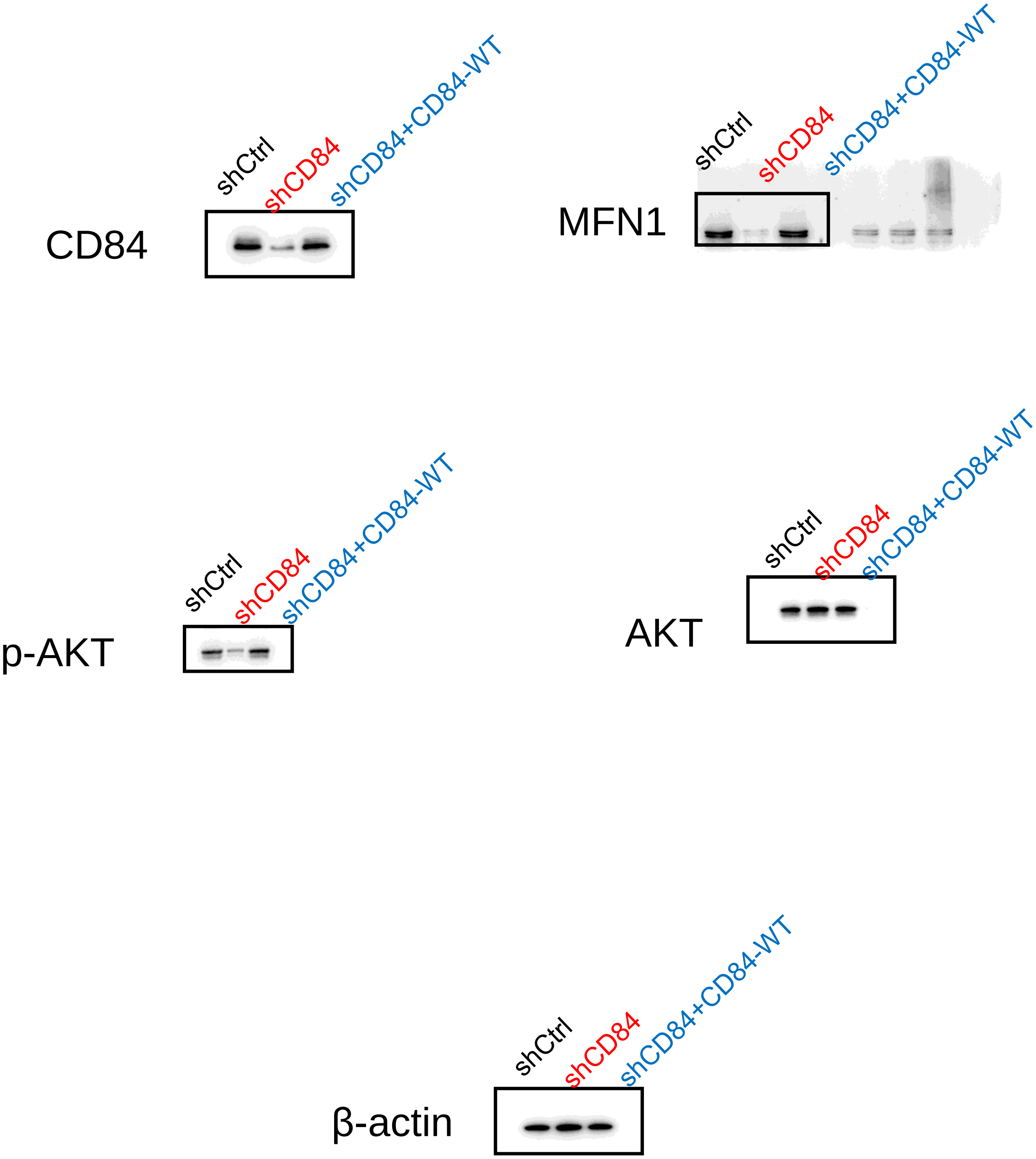

Figure 7F

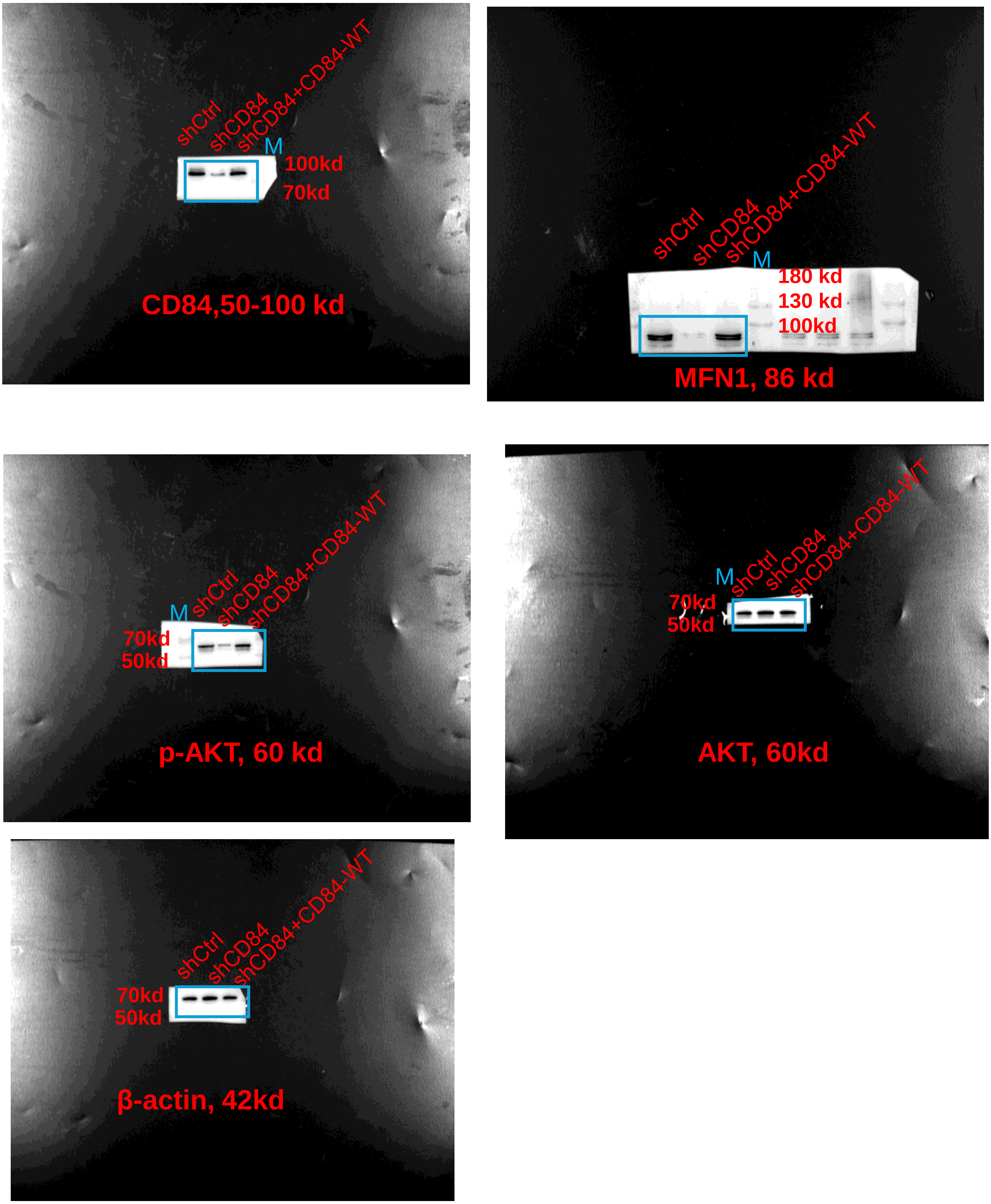
shCtrl
shCD84
shCD84+CD84-WT
M
100kd
70kd
CD84,50-100 kd
shCtrl
shCD84
shCD84+CD84-WT
M
180 kd
130 kd
100kd
MFN1, 86 kd
M
shCtrl
shCD84
shCD84+CD84-WT
70kd
50kd
p-AKT, 60 kd
M
shCtrl
shCD84
shCD84+CD84-WT
70kd
50kd
AKT, 60kd
shCtrl
shCD84
shCD84+CD84-WT
70kd
50kd
β-actin, 42kd

Figure 8D

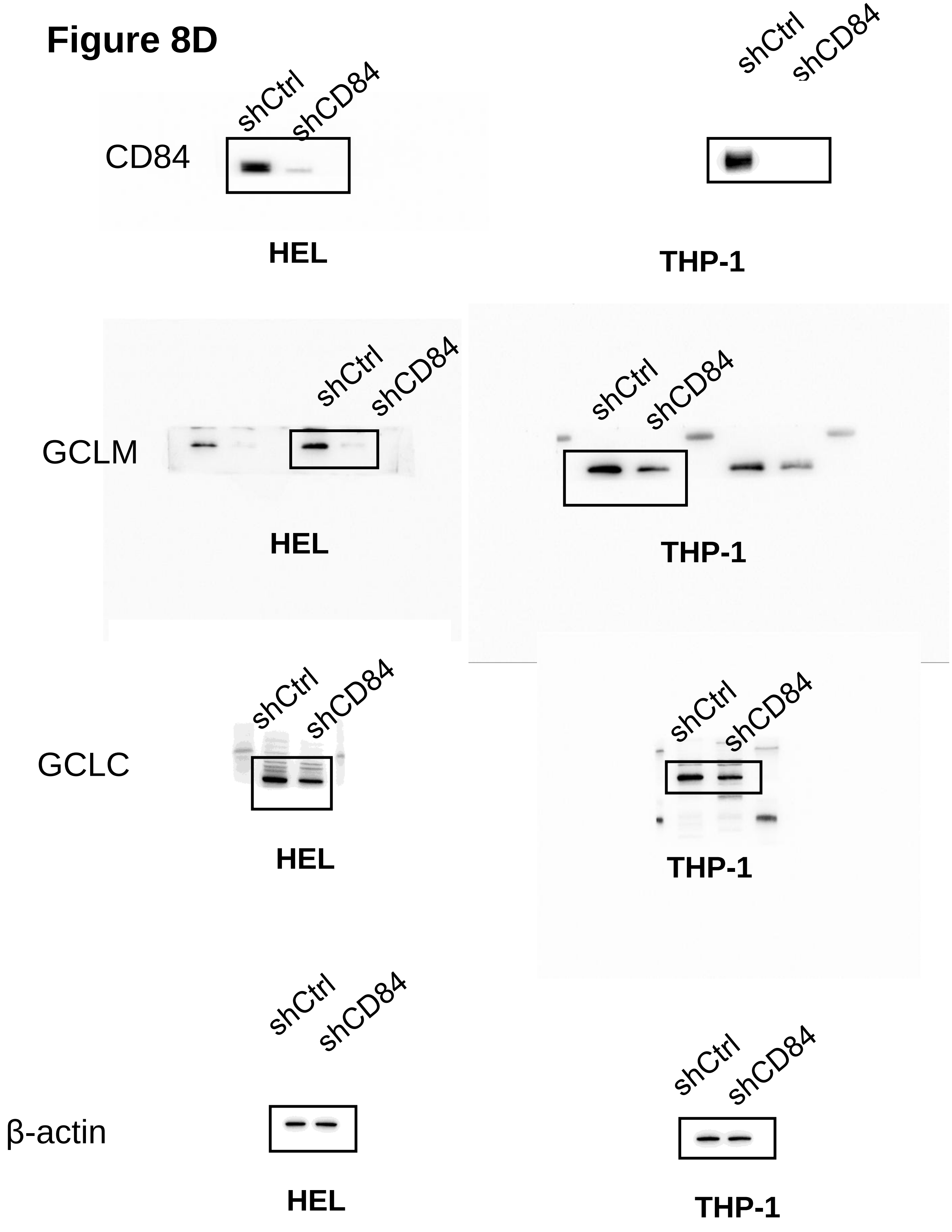

Figure 8D

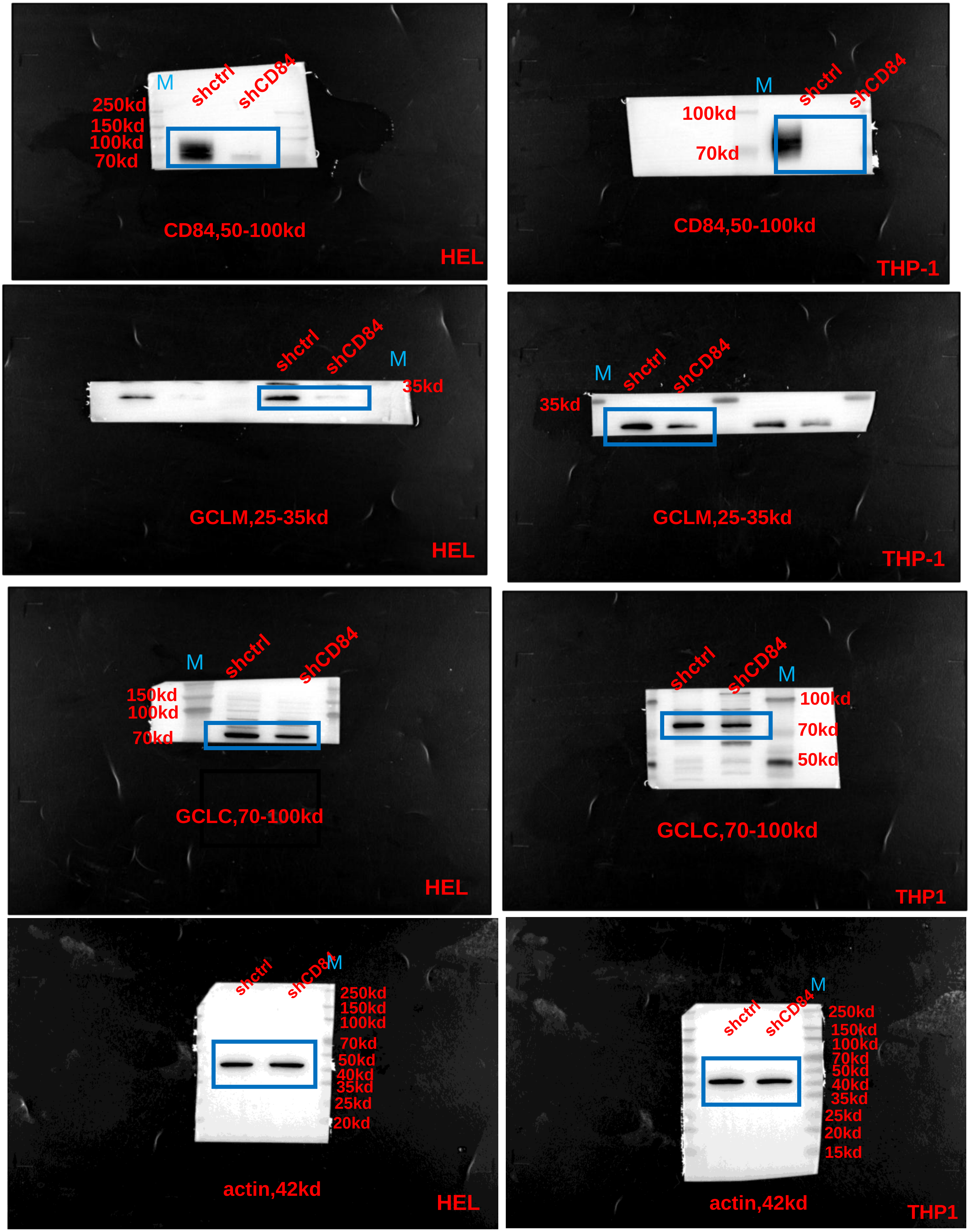

M
shctrl
shCD84
250kd
150kd
100kd
70kd
CD84,50-100kd
HEL
M
shctrl
shCD84
100kd
70kd
CD84,50-100kd
THP-1
shctrl
shCD84
M
35kd
GCLM,25-35kd
HEL
M
shctrl
shCD84
35kd
GCLM,25-35kd
THP-1
M
shctrl
shCD84
150kd
100kd
70kd
GCLC,70-100kd
HEL
shctrl
shCD84
M
100kd
70kd
50kd
GCLC,70-100kd
THP1
shctrl
shCD84
M
250kd
150kd
100kd
70kd
50kd
40kd
35kd
25kd
20kd
actin,42kd
HEL
M
shctrl
shCD84
250kd
150kd
100kd
70kd
50kd
40kd
35kd
25kd
20kd
15kd
actin,42kd
THP1

Figure 8H left panel

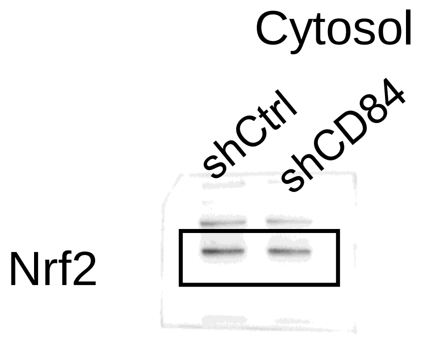

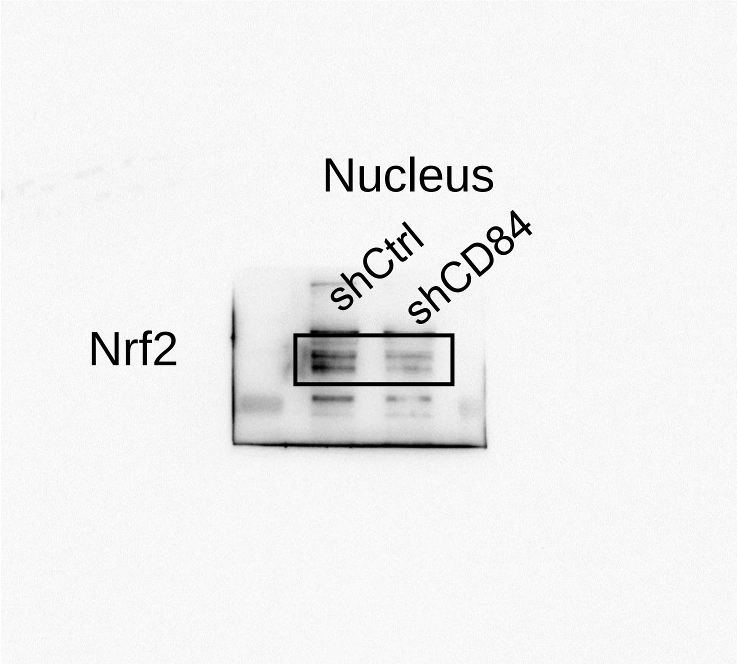

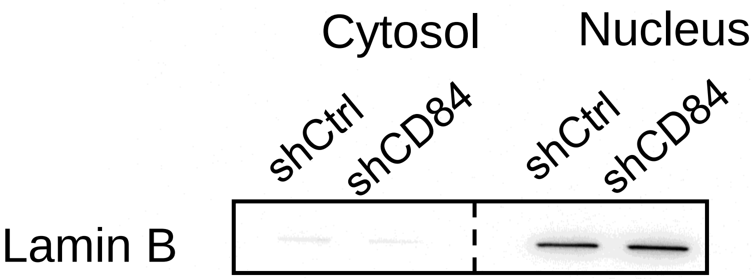

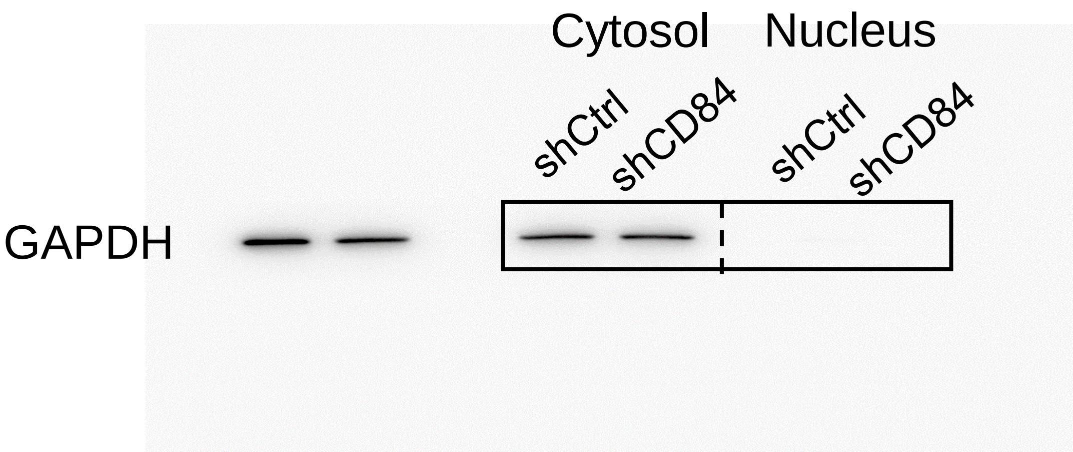

Figure 8H left panel

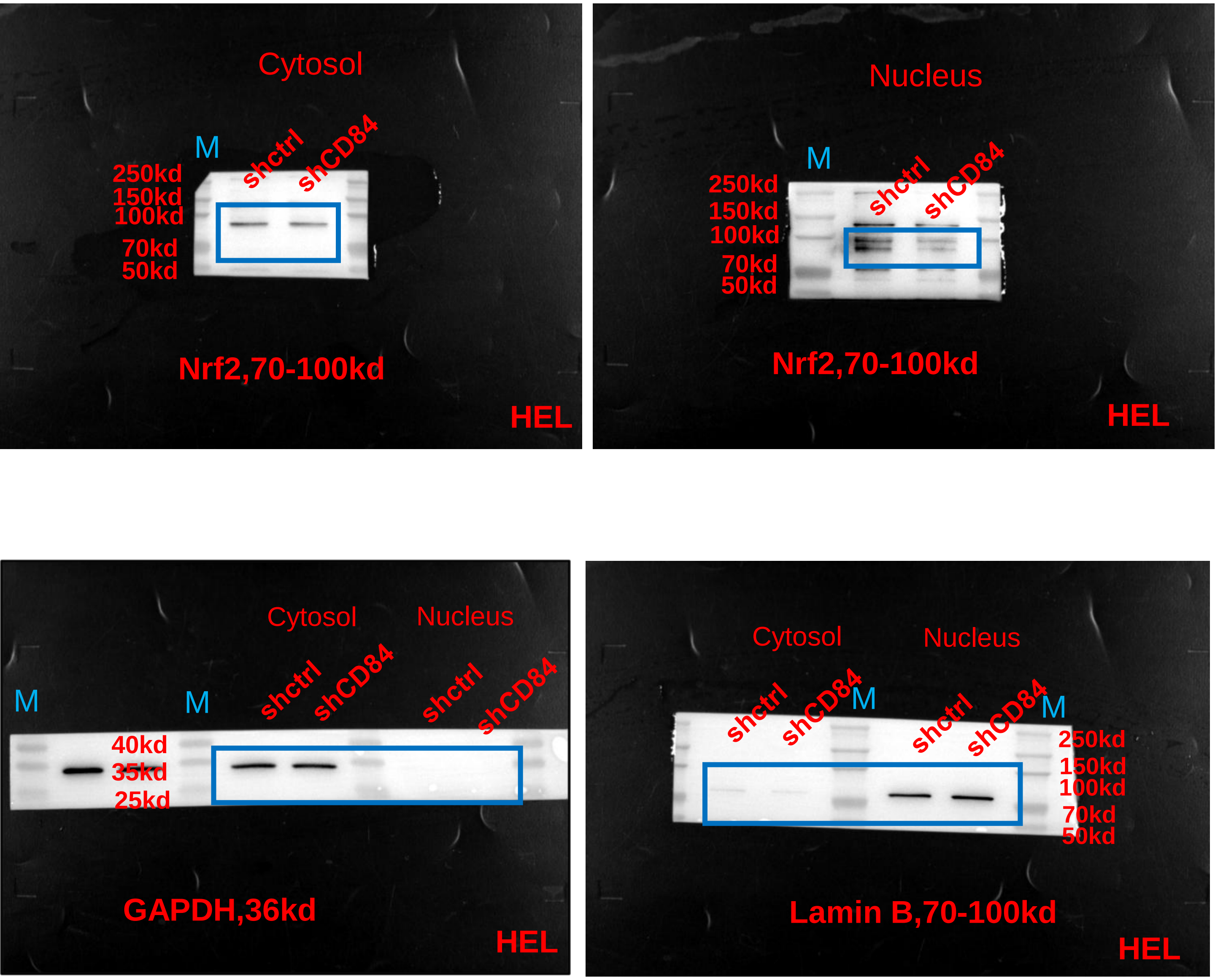

Figure 8H right panel

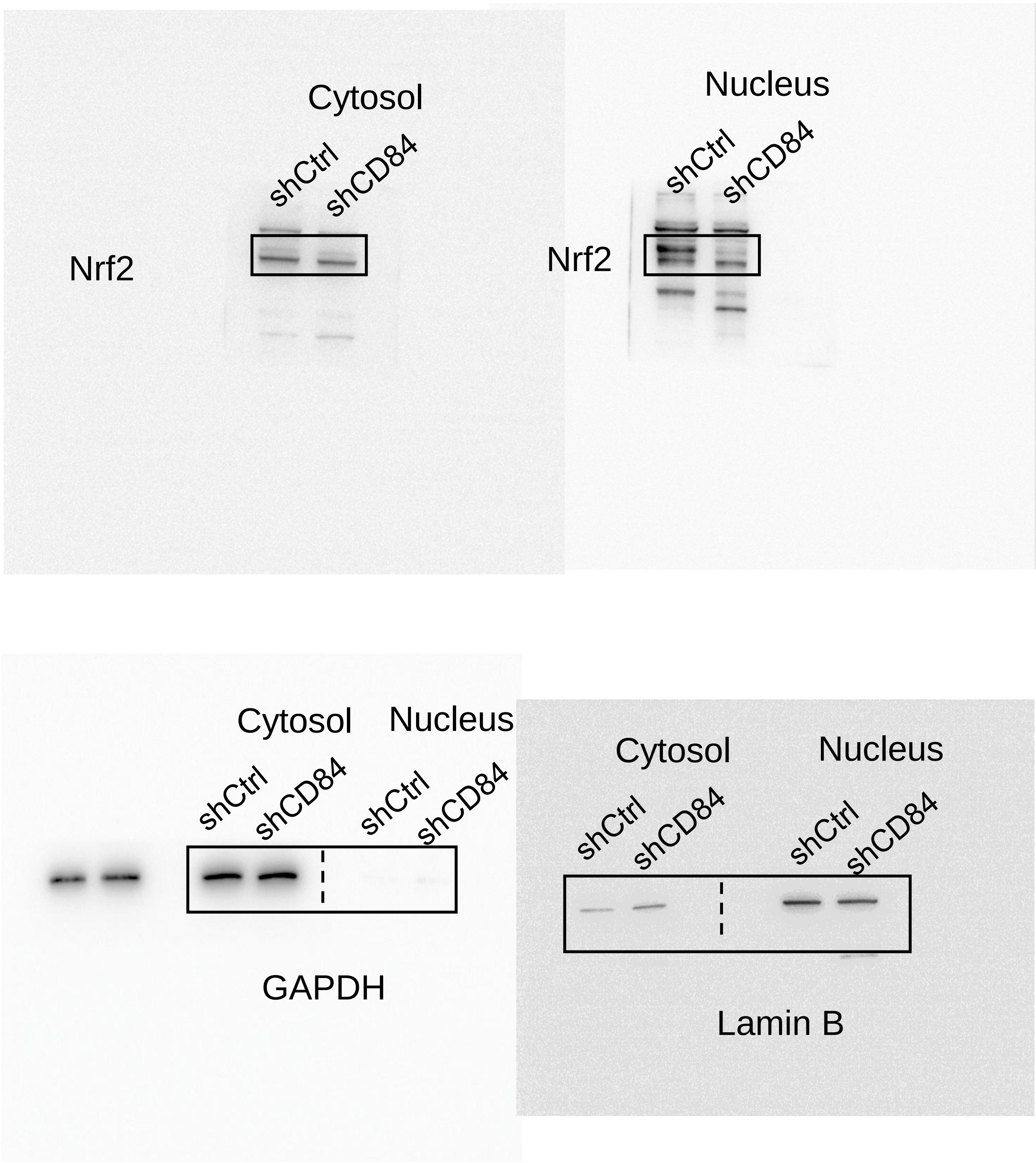

Figure 8H right panel

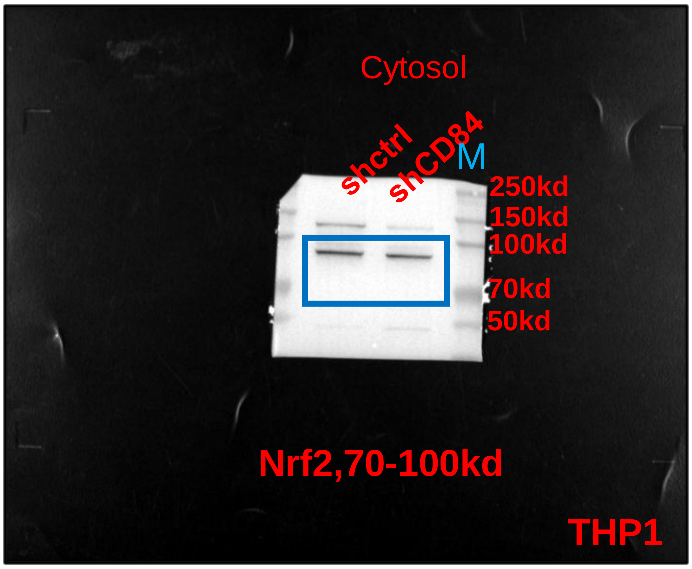

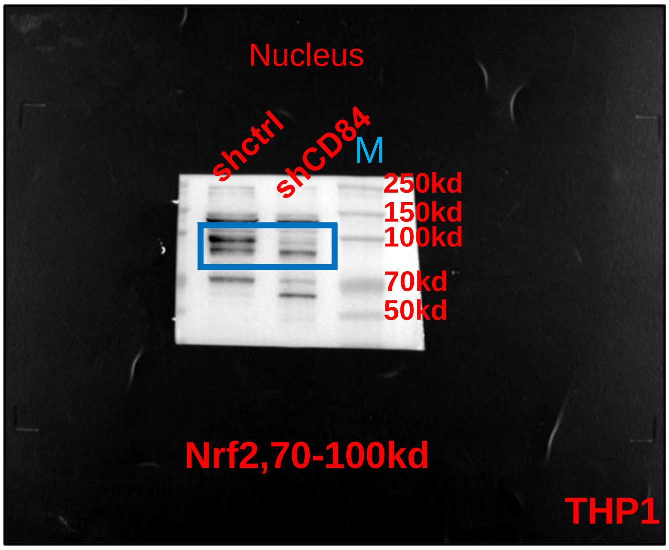

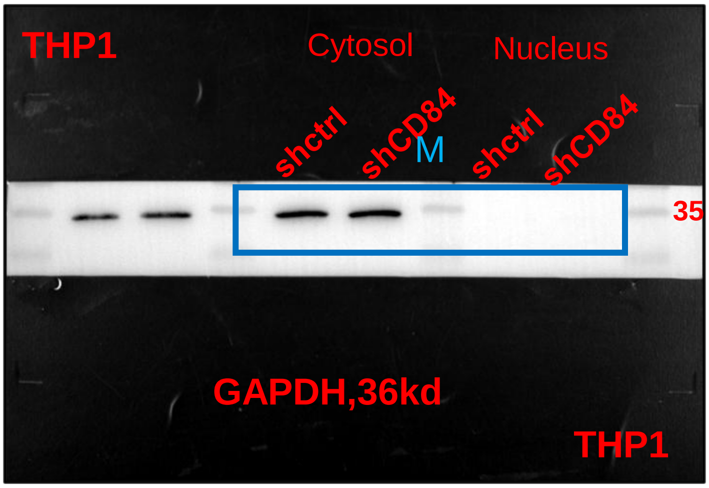

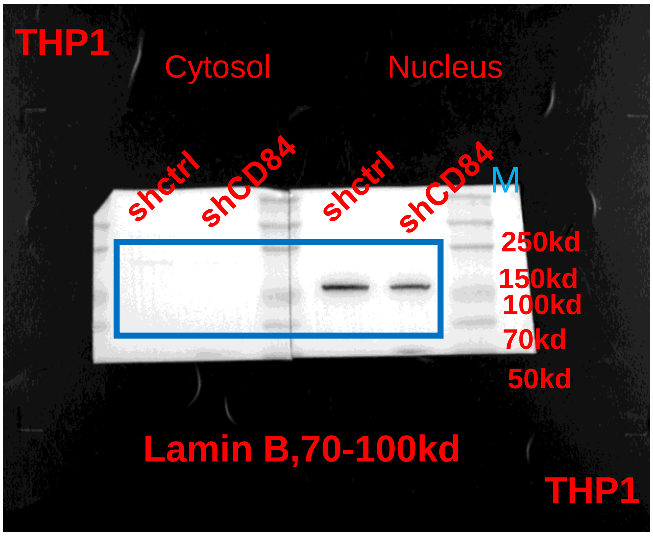

Figure 9A left panel

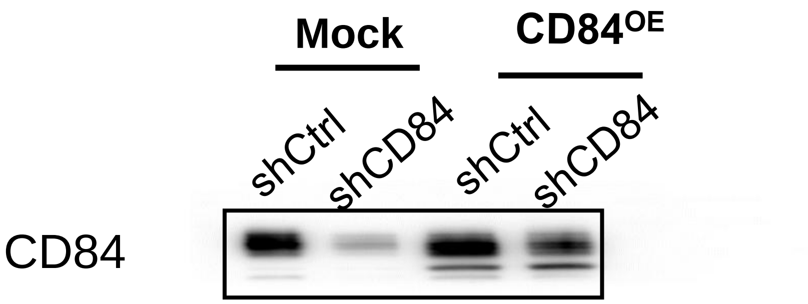

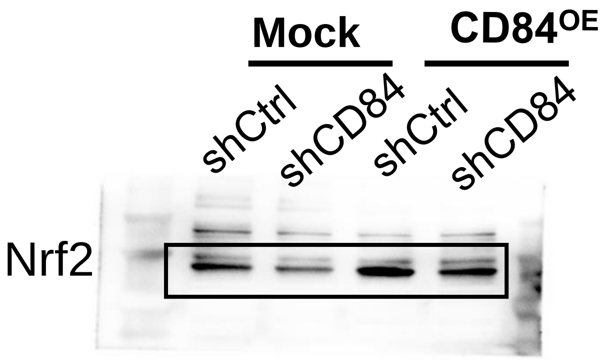

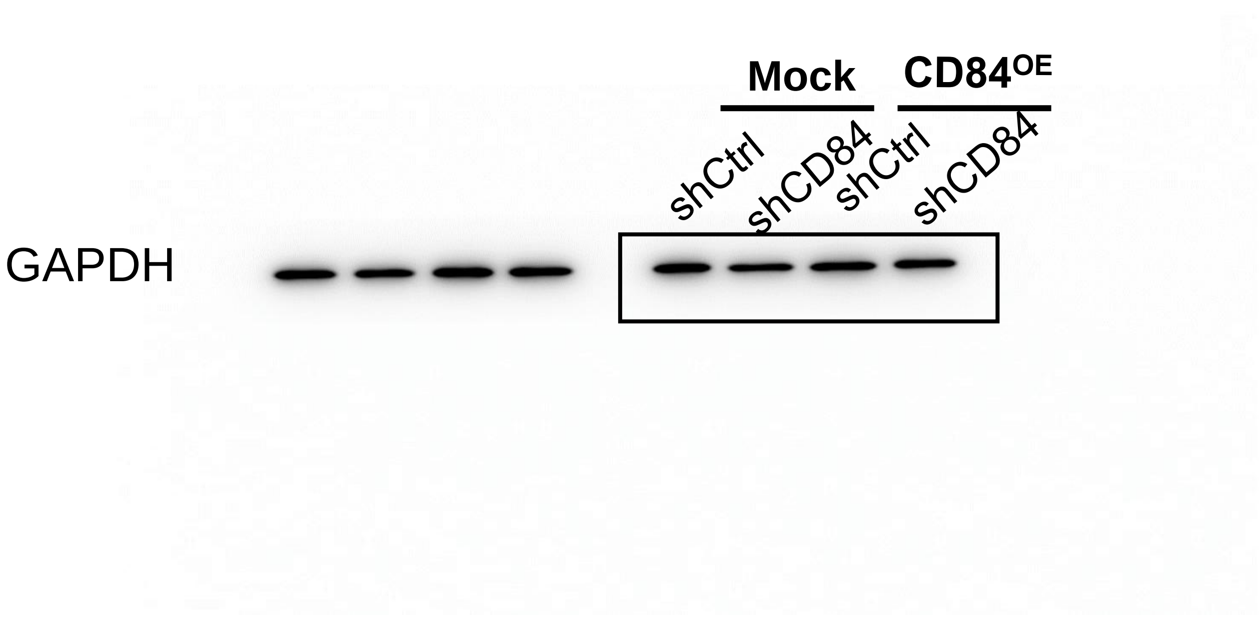

Figure 9A left panel

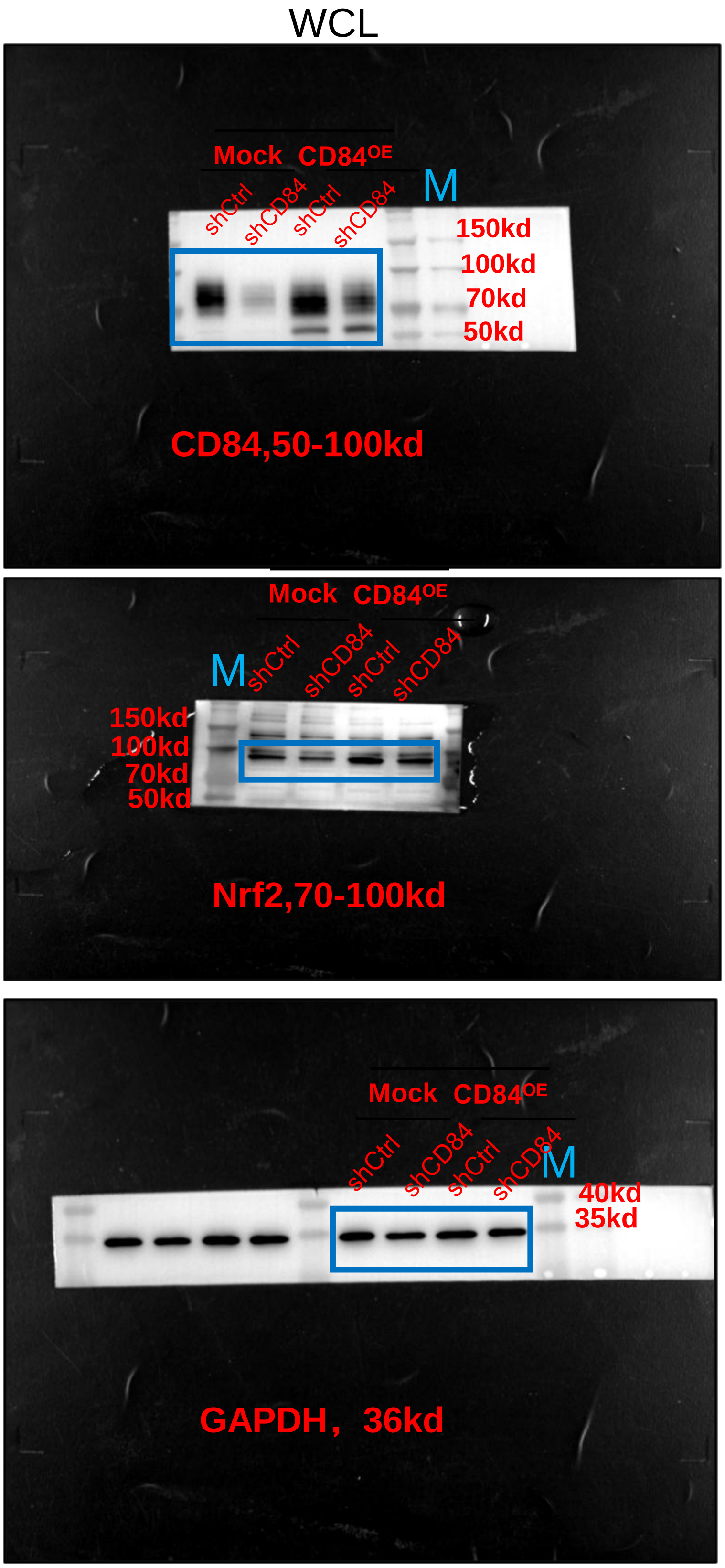

Figure 9A right panel

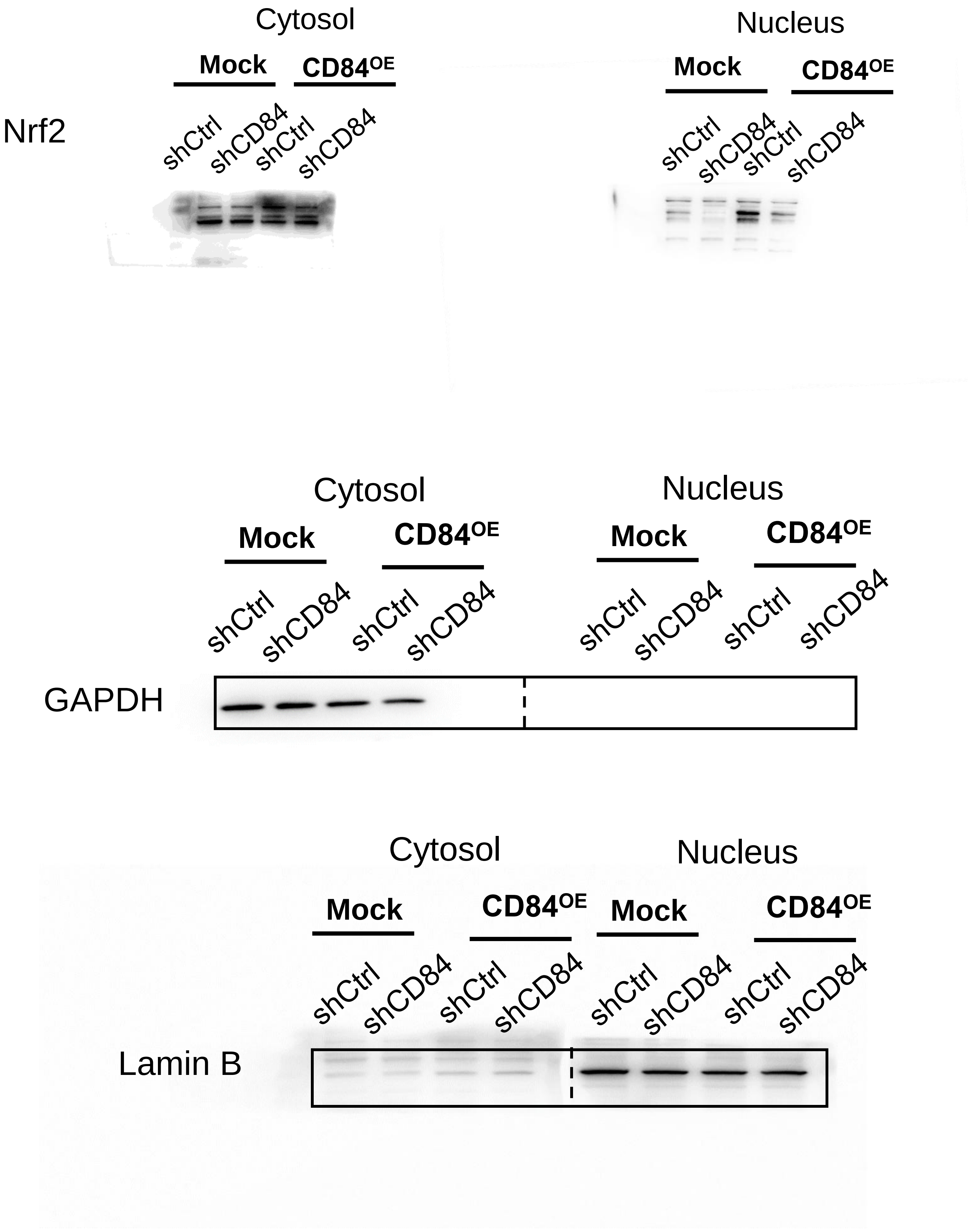

Figure 9A right panel

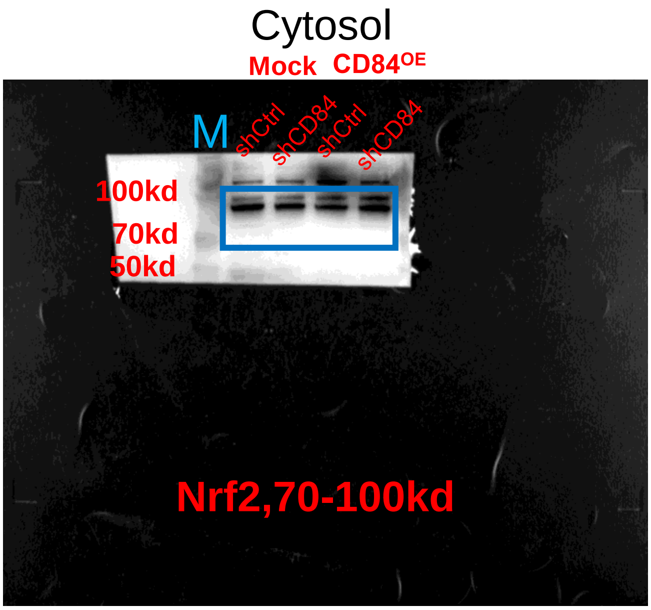

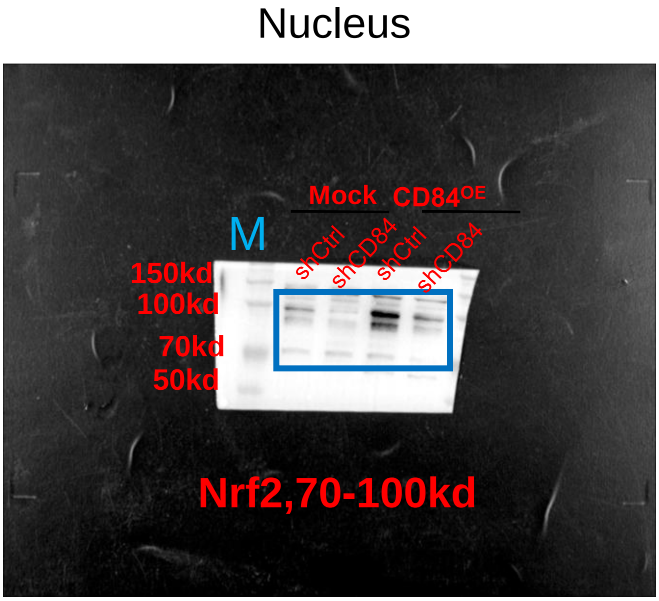

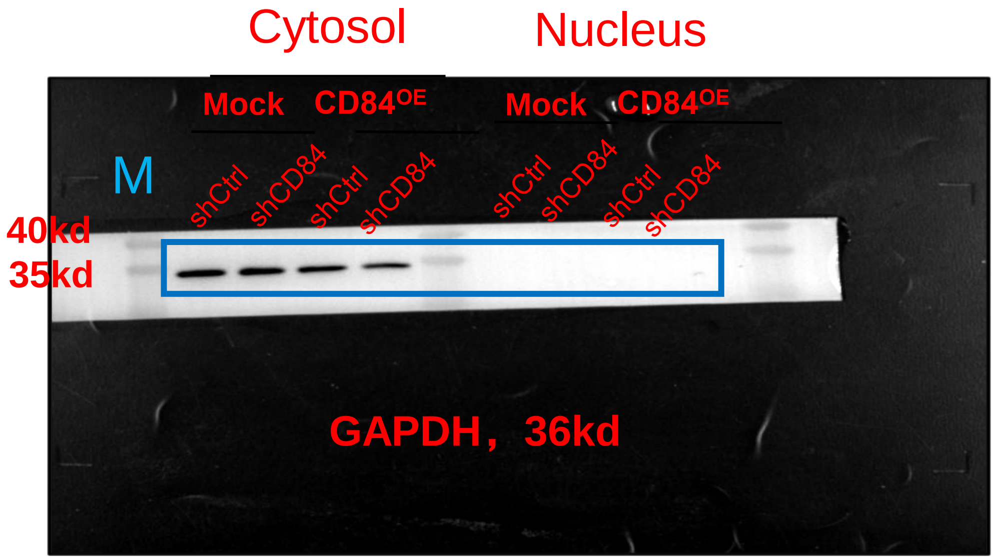

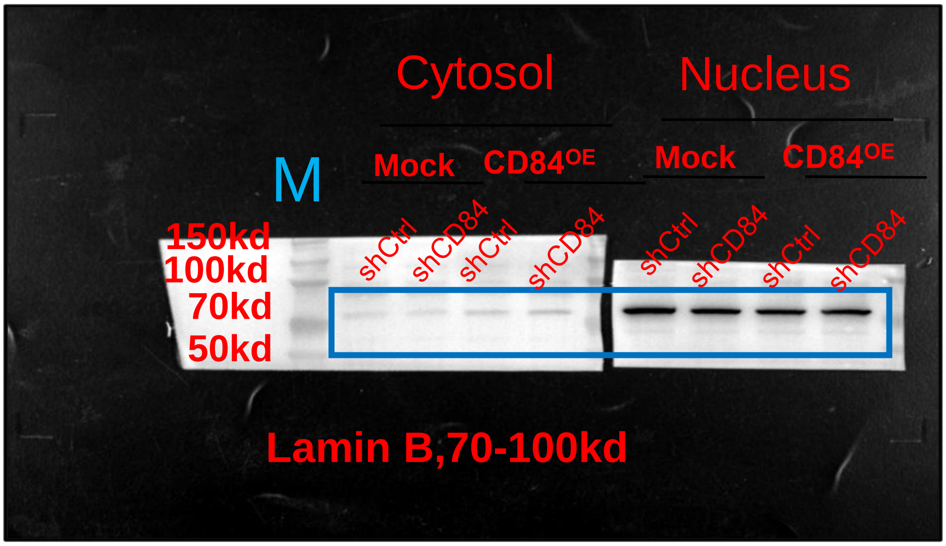

Figure 9B

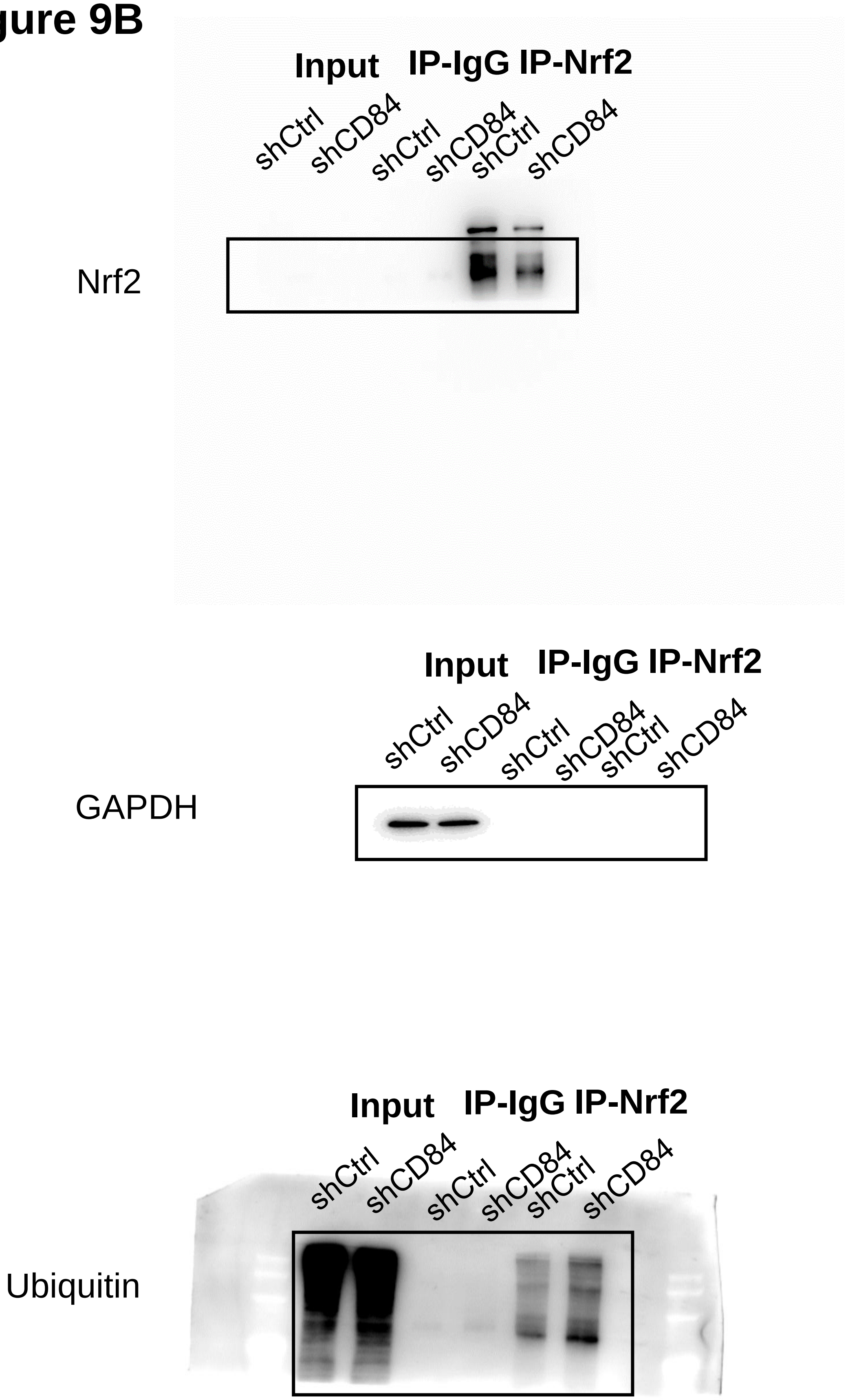

Input
IP-IgG
IP-Nrf2
shCtrl
shCD84
shCtrl
shCD84
shCtrl
shCD84
Nrf2
Input
IP-IgG
IP-Nrf2
shCtrl
shCD84
shCtrl
shCD84
shCtrl
shCD84
GAPDH
Input
IP-IgG
IP-Nrf2
shCtrl
shCD84
shCtrl
shCD84
shCtrl
shCD84
Ubiquitin

Figure 9B

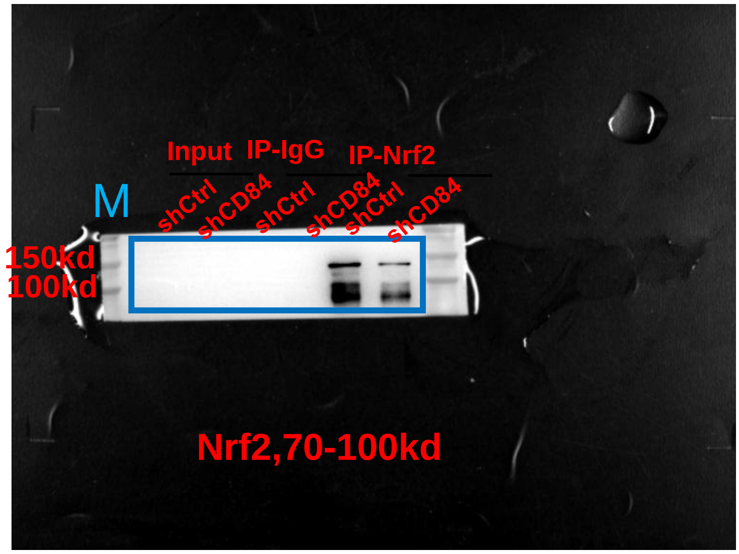

Input
IP-IgG
IP-Nrf2
M
shCtrl
shCD84
shCtrl
shCD84
shCtrl
shCD84
150kd
100kd
Nrf2,70-100kd

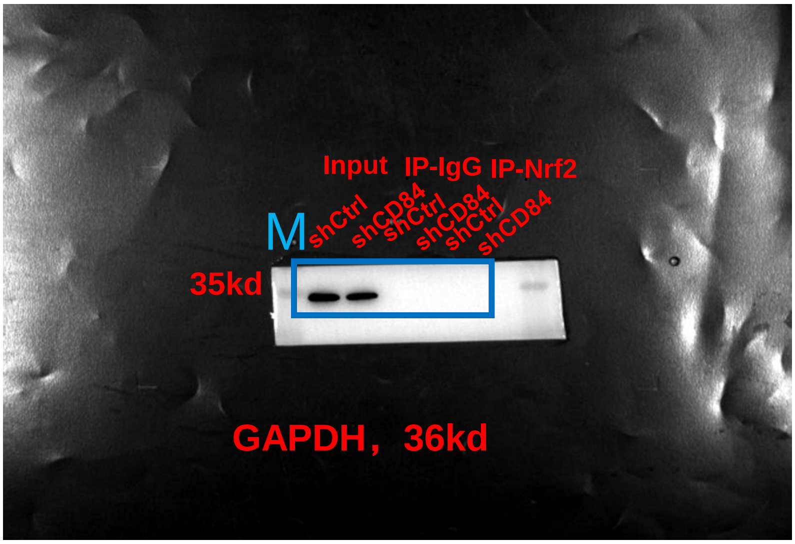

Input
IP-IgG
IP-Nrf2
M
shCtrl
shCD84
shCtrl
shCD84
shCtrl
shCD84
35kd
GAPDH, 36kd

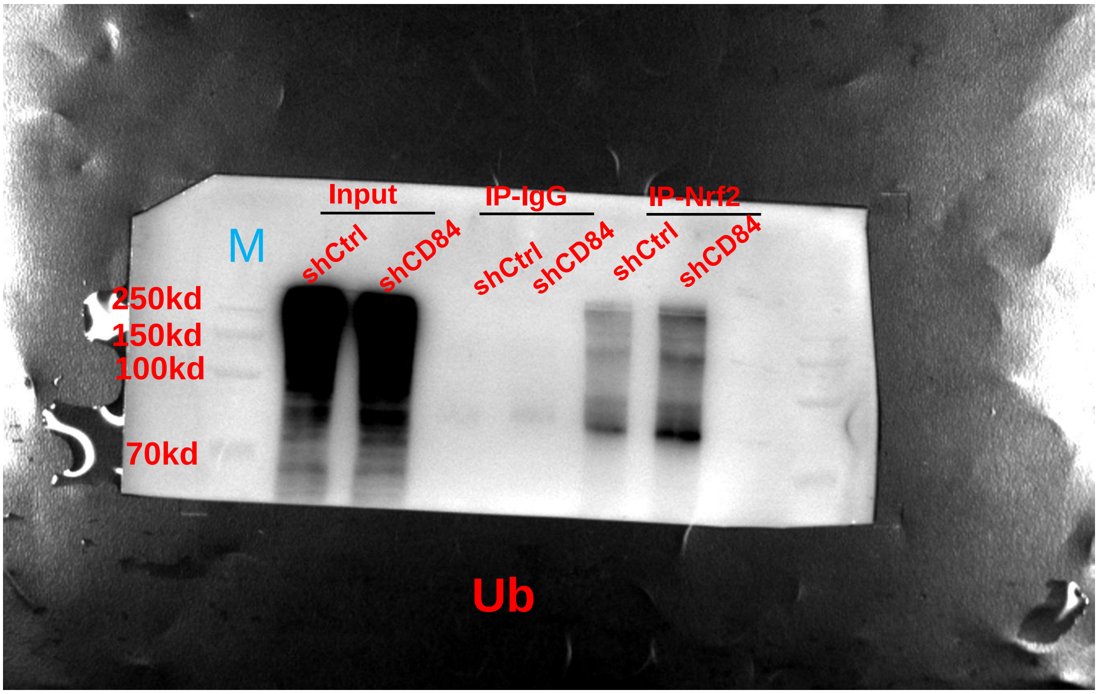

Input
IP-IgG
IP-Nrf2
M
shCtrl
shCD84
shCtrl
shCD84
shCtrl
shCD84
250kd
150kd
100kd
70kd
Ub

Figure 9C

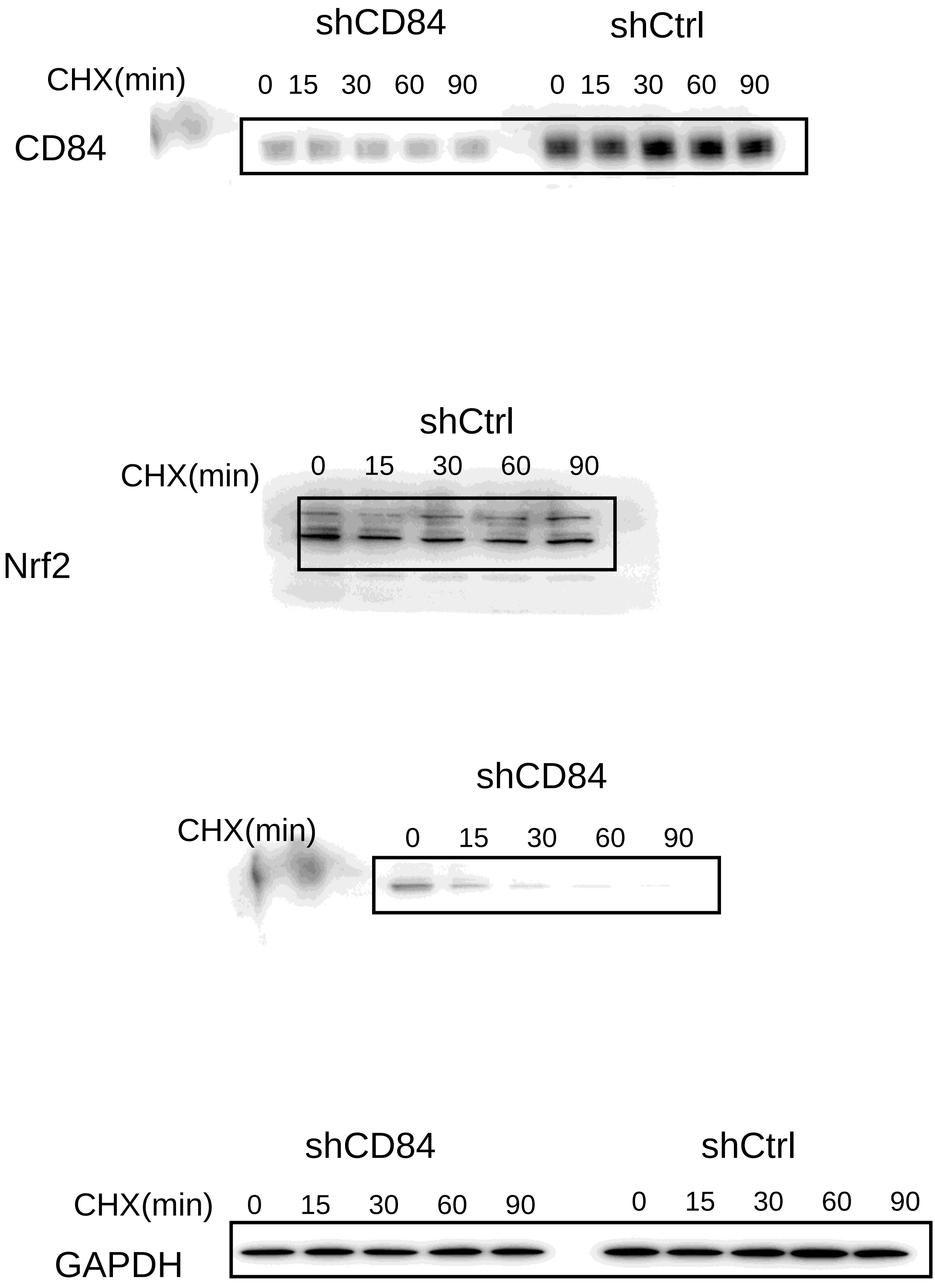
shCD84
shCtrl
CHX(min)
0 15 30 60 90
0 15 30 60 90
CD84
shCtrl
CHX(min)
0 15 30 60 90
Nrf2
shCD84
CHX(min)
0 15 30 60 90
shCD84
shCtrl
CHX(min)
0 15 30 60 90
0 15 30 60 90
GAPDH

Figure 9C

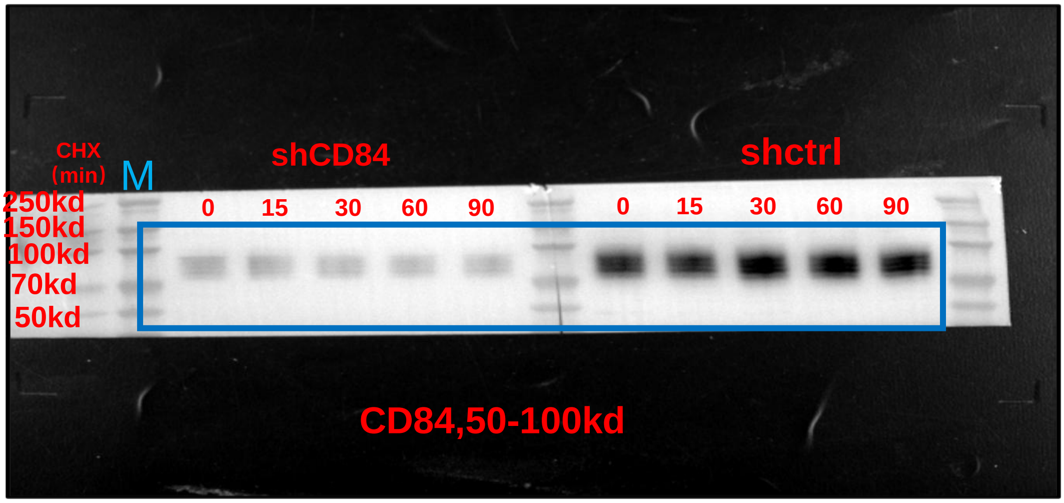

CHX (min)
M
shCD84
shctrl
250kd
150kd
100kd
70kd
50kd
0 15 30 60 90
0 15 30 60 90
CD84,50-100kd

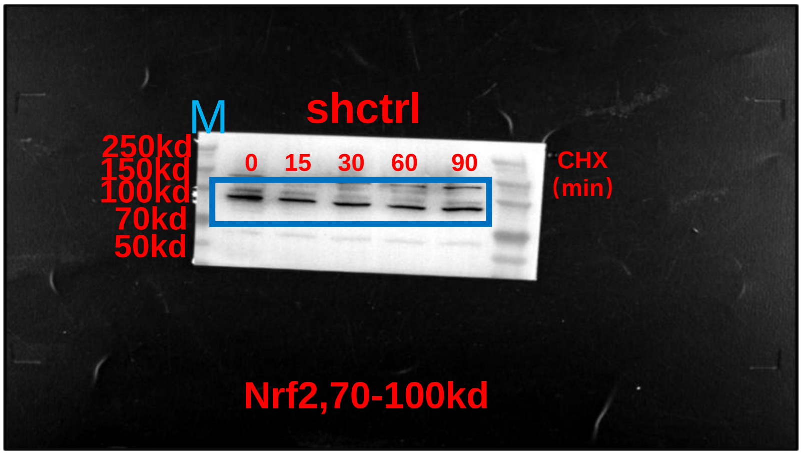

M
shctrl
250kd
150kd
100kd
70kd
50kd
0 15 30 60 90
CHX (min)
Nrf2,70-100kd

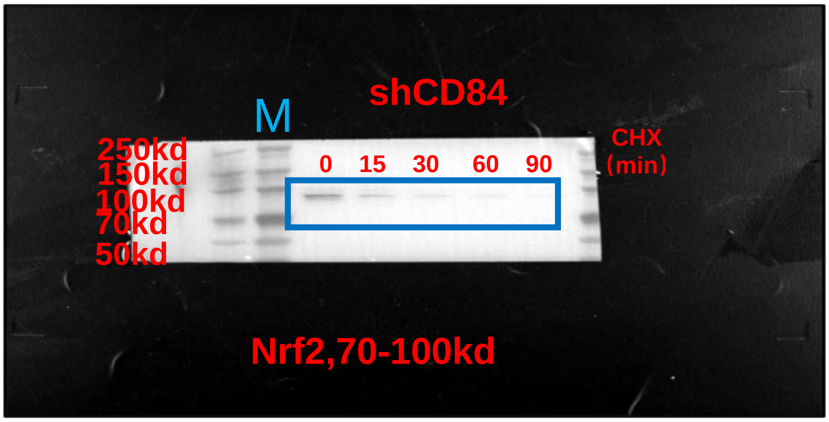

M
shCD84
250kd
150kd
100kd
70kd
50kd
0 15 30 60 90
CHX (min)
Nrf2,70-100kd

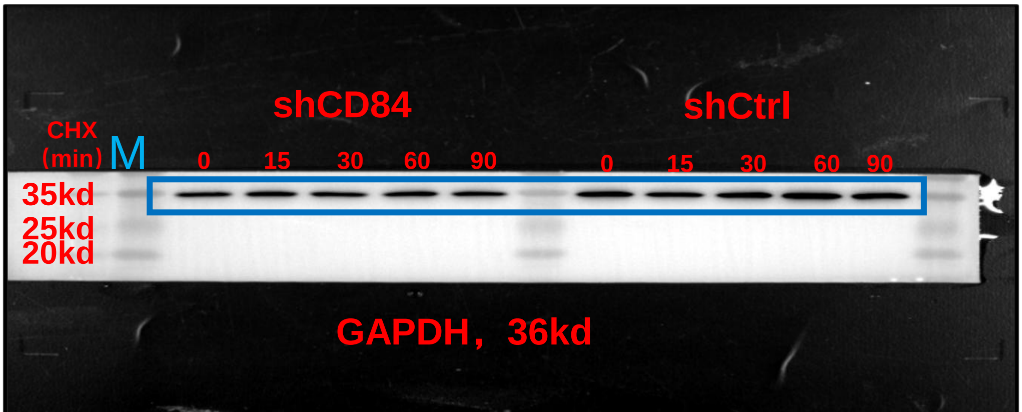

CHX (min)
M
shCD84
shCtrl
0 15 30 60 90
0 15 30 60 90
35kd
25kd
20kd
GAPDH，36kd

Figure 9D

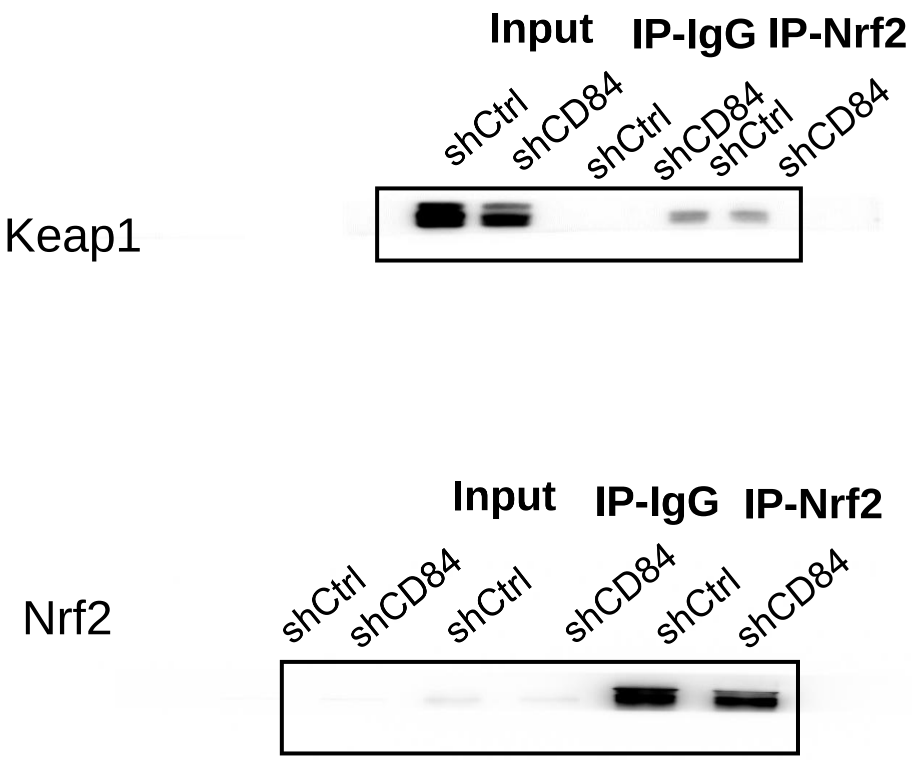

Input
IP-IgG
IP-Nrf2
shCtrl
shCD84
shCtrl
shCD84
shCtrl
shCD84
Keap1
Input
IP-IgG
IP-Nrf2
shCtrl
shCD84
shCtrl
shCD84
shCtrl
shCD84
Nrf2

Figure 9D

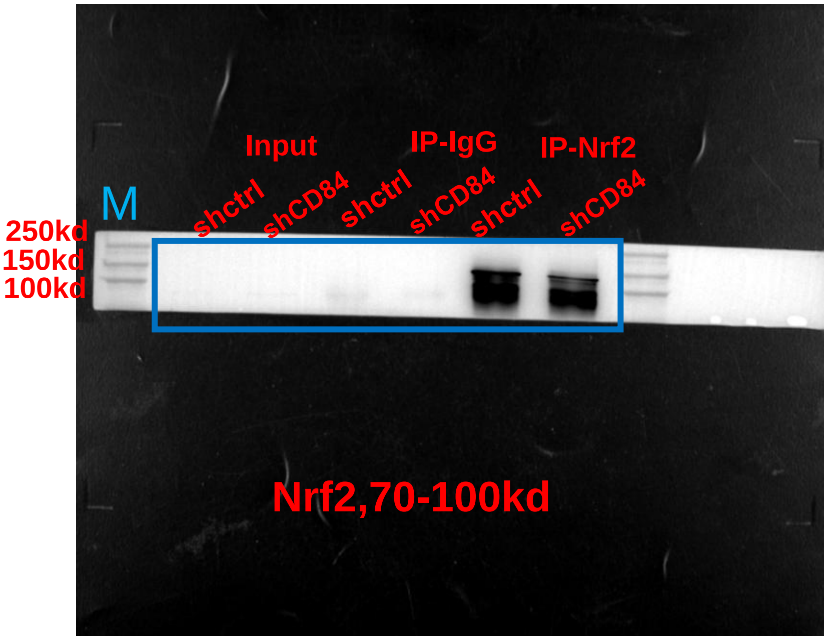
Input
IP-IgG
IP-Nrf2
M
shctrl
shCD84
shctrl
shCD84
shctrl
shCD84
250kd
150kd
100kd
Nrf2,70-100kd

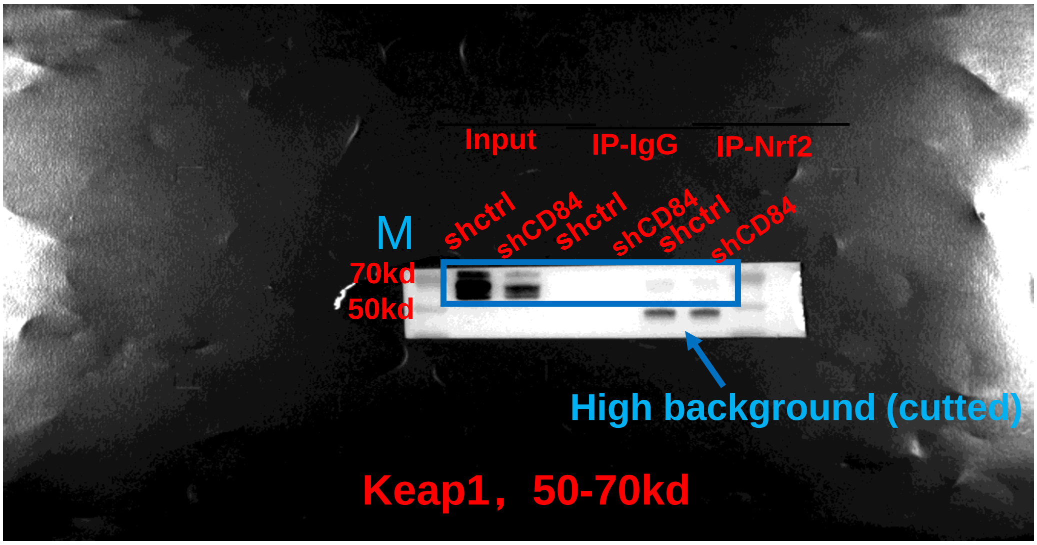
Input
IP-IgG
IP-Nrf2
M
shctrl
shCD84
shctrl
shCD84
shctrl
shCD84
70kd
50kd
High background (cutted)
Keap1，50-70kd

Figure 9E

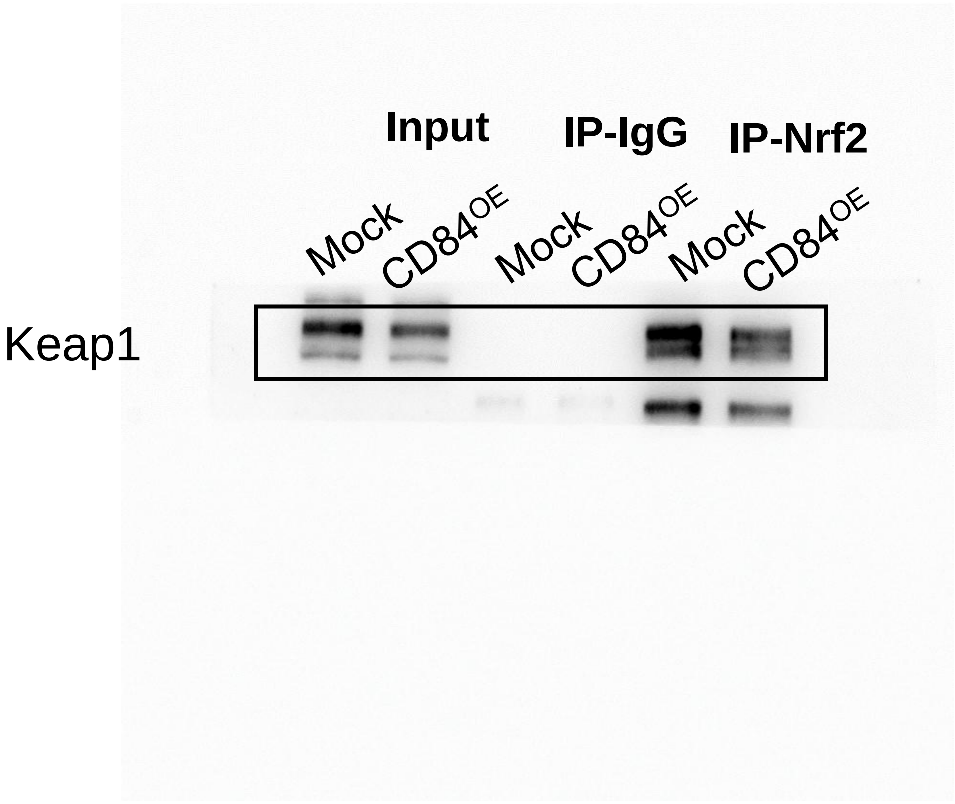

Input
IP-IgG
IP-Nrf2
Mock
CD84OE
Mock
CD84OE
Mock
CD84OE
Keap1

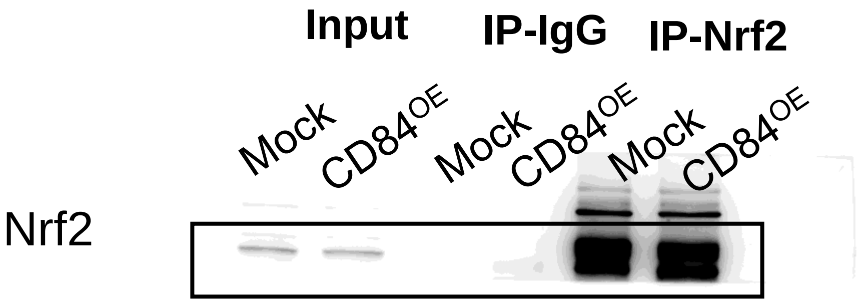

Input
IP-IgG
IP-Nrf2
Mock
CD84OE
Mock
CD84OE
Mock
CD84OE
Nrf2

Figure 9E

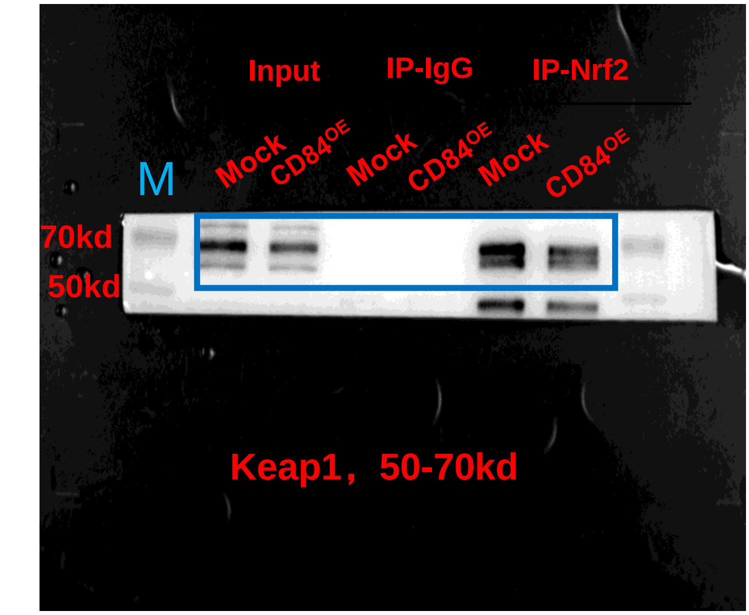
Input
IP-IgG
IP-Nrf2
M
Mock
CD84OE
Mock
CD84OE
Mock
CD84OE
70kd
50kd
Keap1，50-70kd

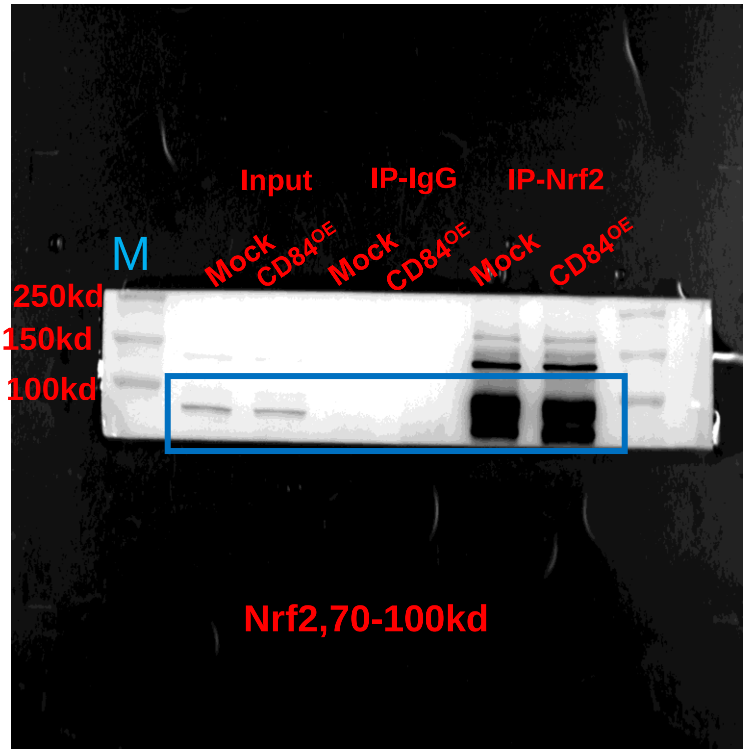
Input
IP-IgG
IP-Nrf2
M
Mock
CD84OE
Mock
CD84OE
Mock
CD84OE
250kd
150kd
100kd
Nrf2,70-100kd

Figure S6A

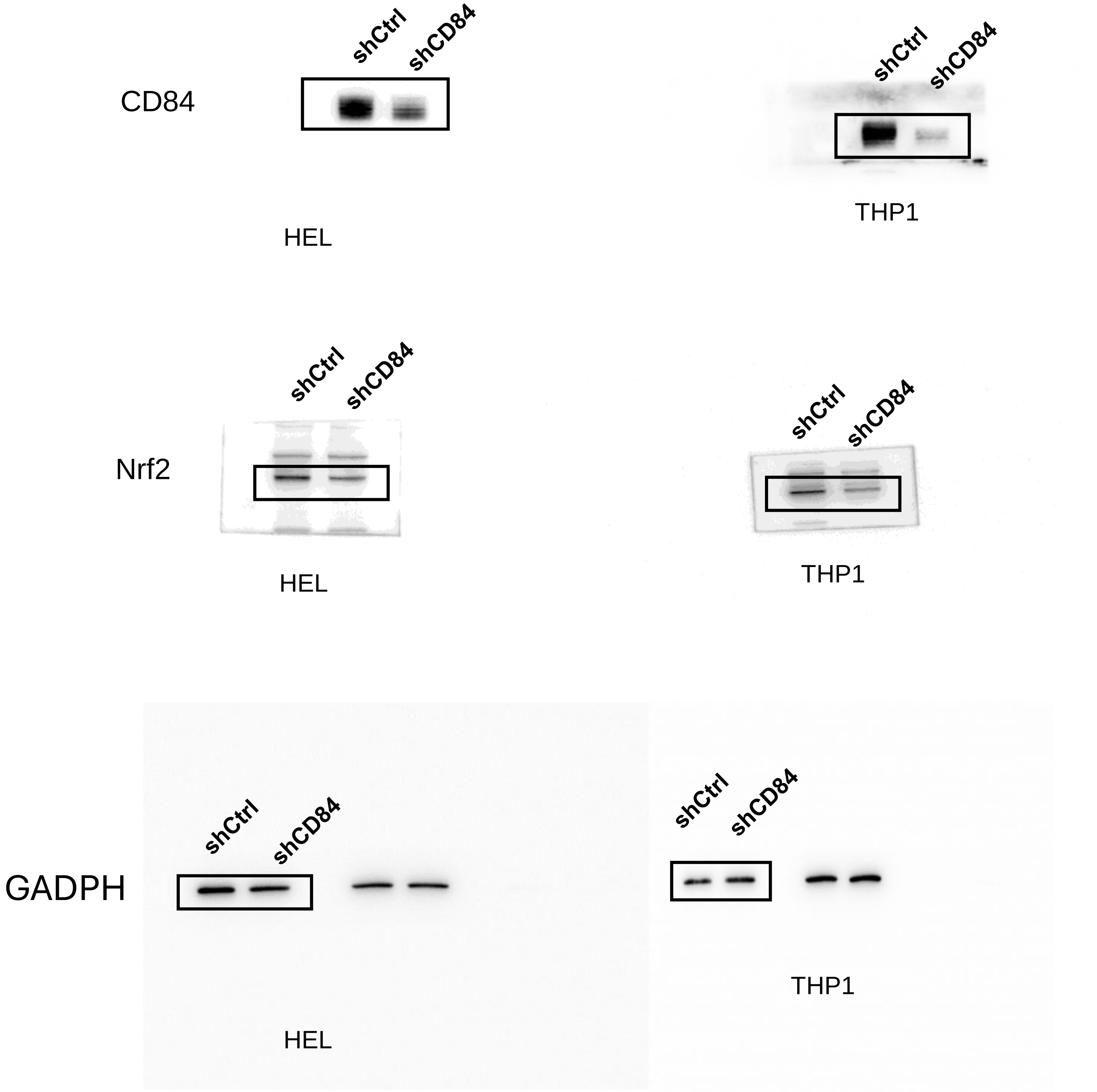

Figure S6A

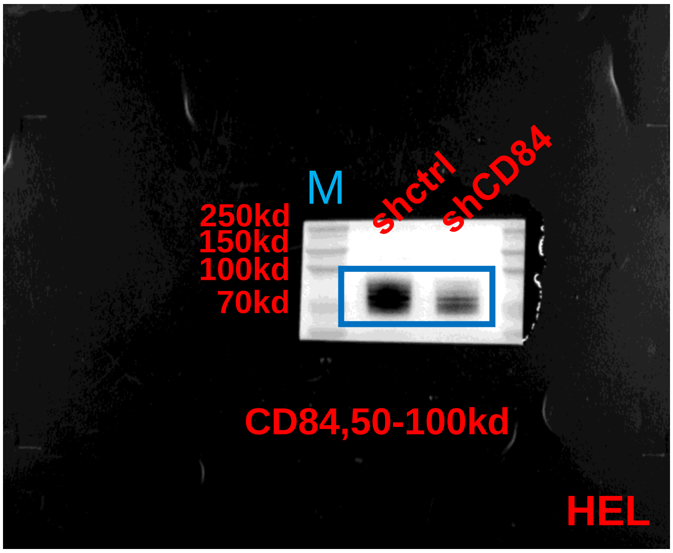

M
shctrl
shCD84
250kd
150kd
100kd
70kd
CD84,50-100kd
HEL

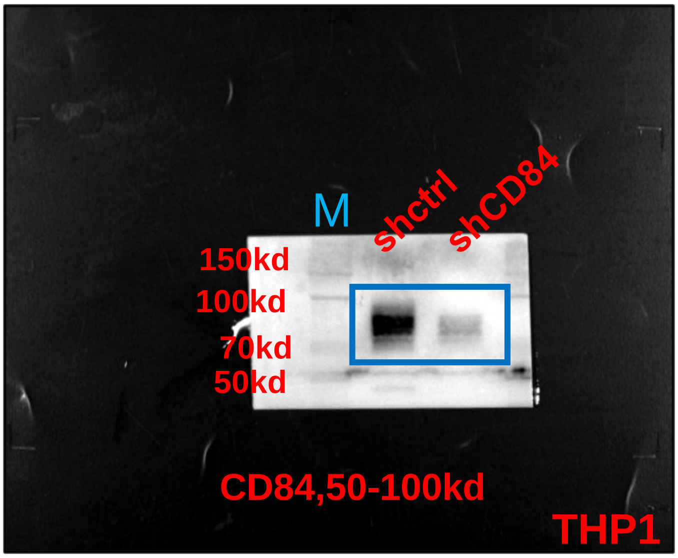

M
shctrl
shCD84
150kd
100kd
70kd
50kd
CD84,50-100kd
THP1

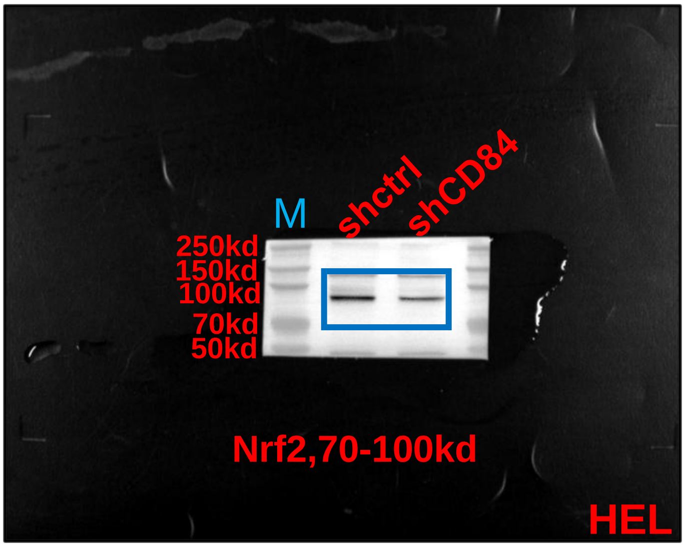

M
shctrl
shCD84
250kd
150kd
100kd
70kd
50kd
Nrf2,70-100kd
HEL

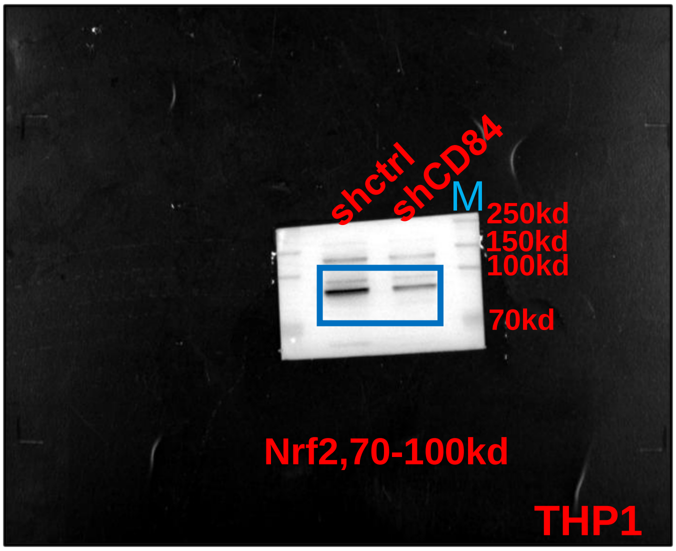

shctrl
shCD84
M
250kd
150kd
100kd
70kd
Nrf2,70-100kd
THP1

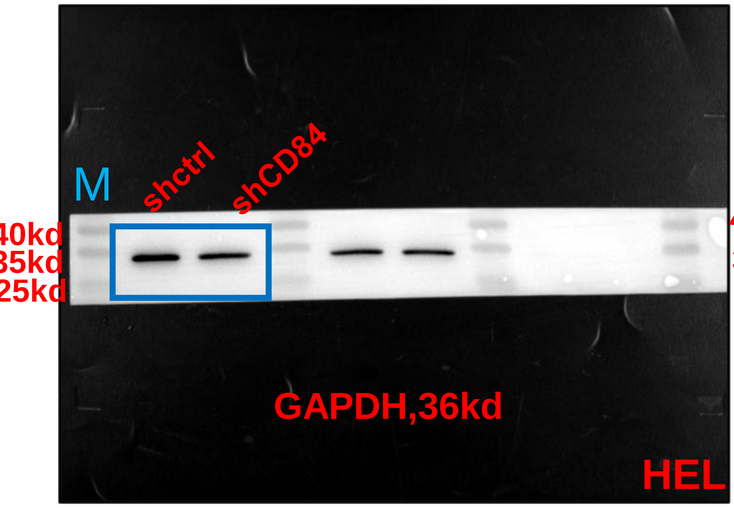

M
shctrl
shCD84
40kd
35kd
25kd
GAPDH,36kd
HEL

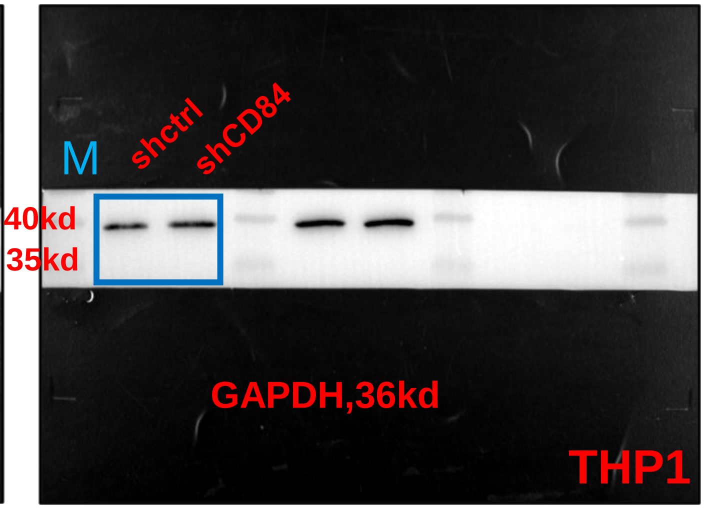

M
shctrl
shCD84
40kd
35kd
GAPDH,36kd
THP1

Figure S6B

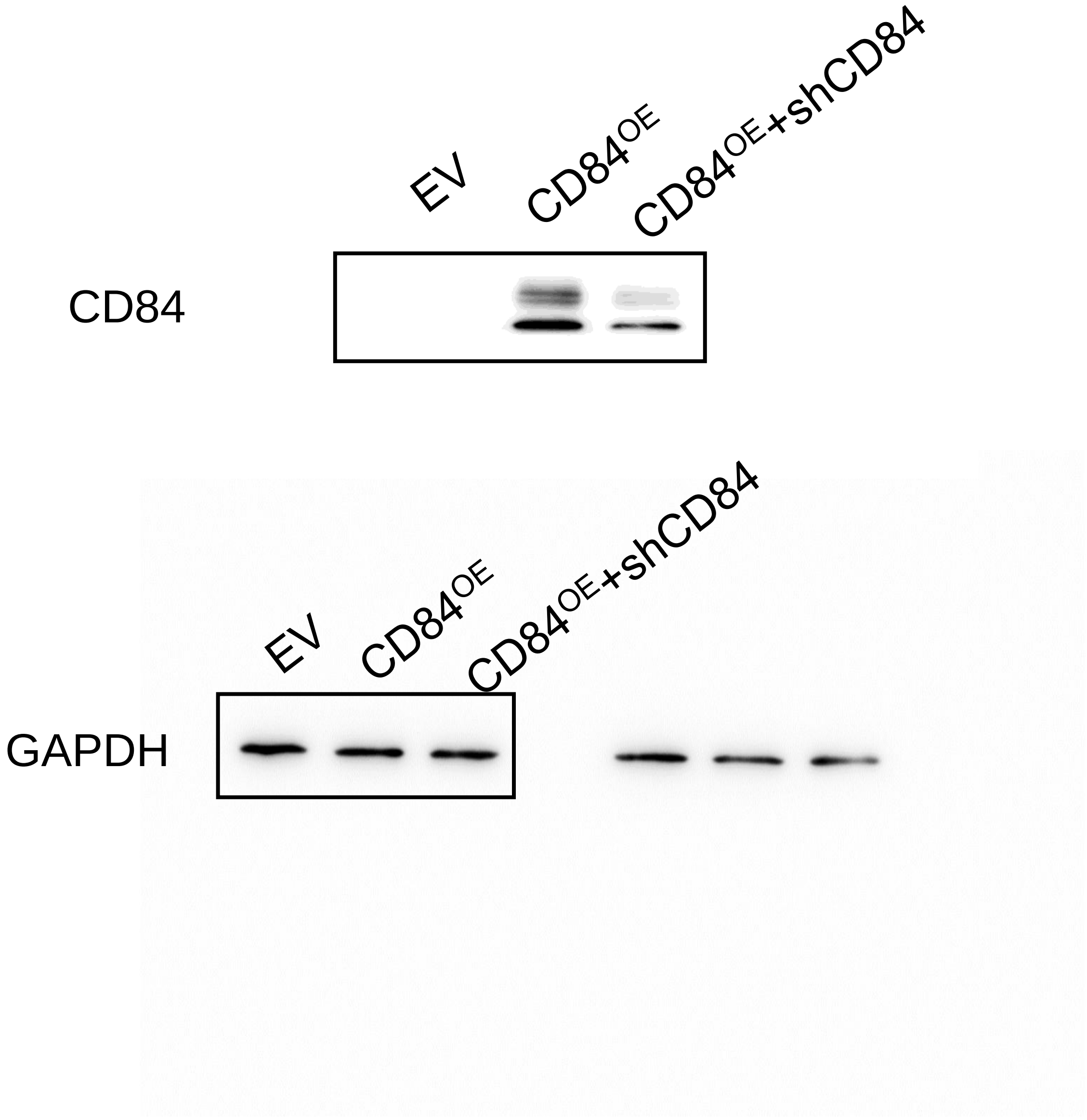

Figure S6B

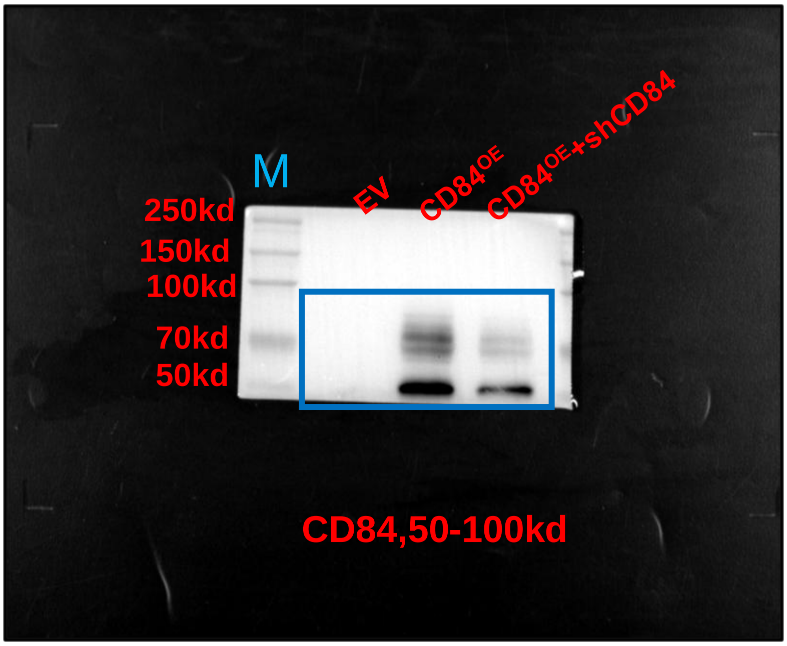
M
EV
CD84OE
CD84OE+shCD84
250kd
150kd
100kd
70kd
50kd
CD84,50-100kd

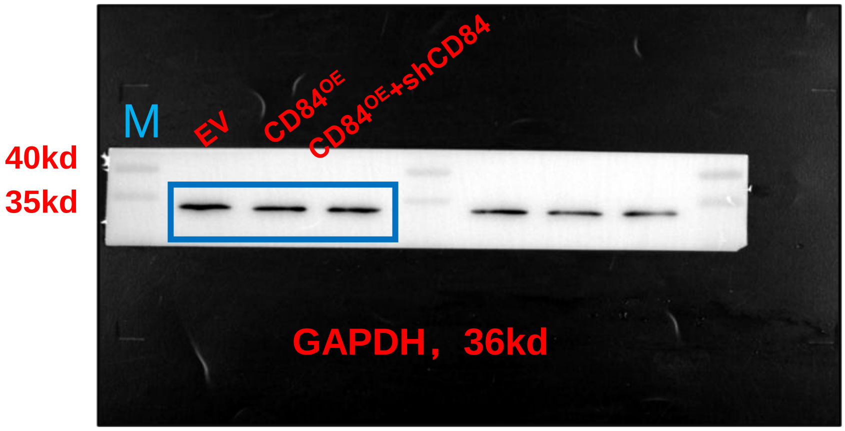
M
EV
CD84OE
CD84OE+shCD84
40kd
35kd
GAPDH，36kd

Figure S6D

CD84

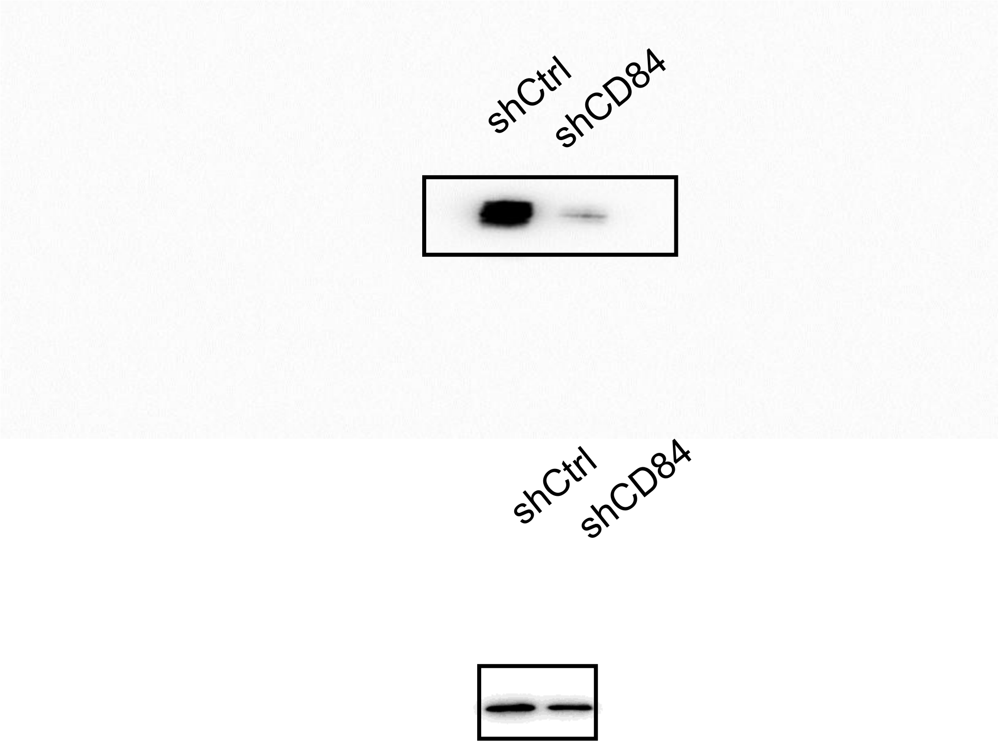

GAPDH

Figure S6D

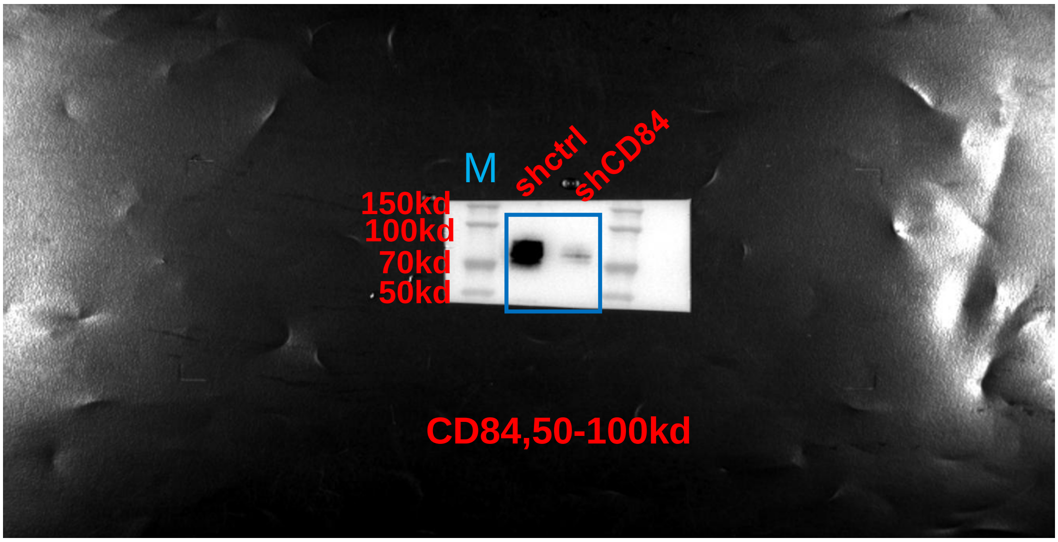
M
shctrl
shCD84
150kd
100kd
70kd
50kd
CD84,50-100kd

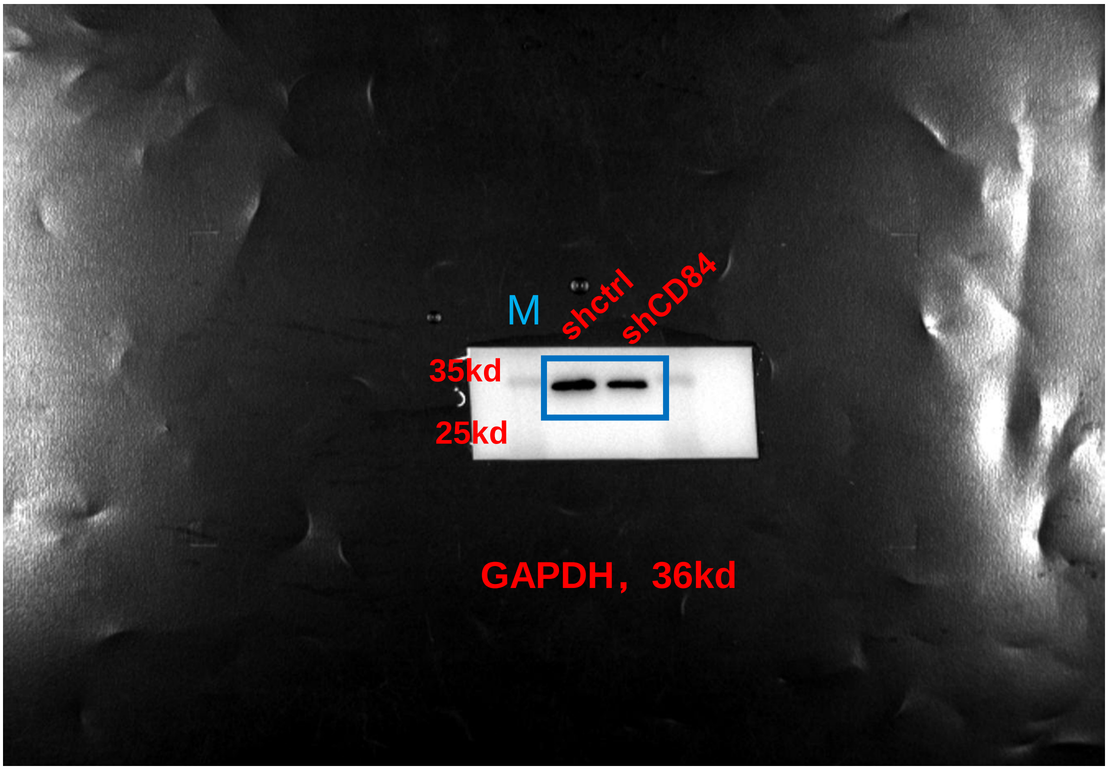
M
shctrl
shCD84
35kd
25kd
GAPDH，36kd

Figure S6E

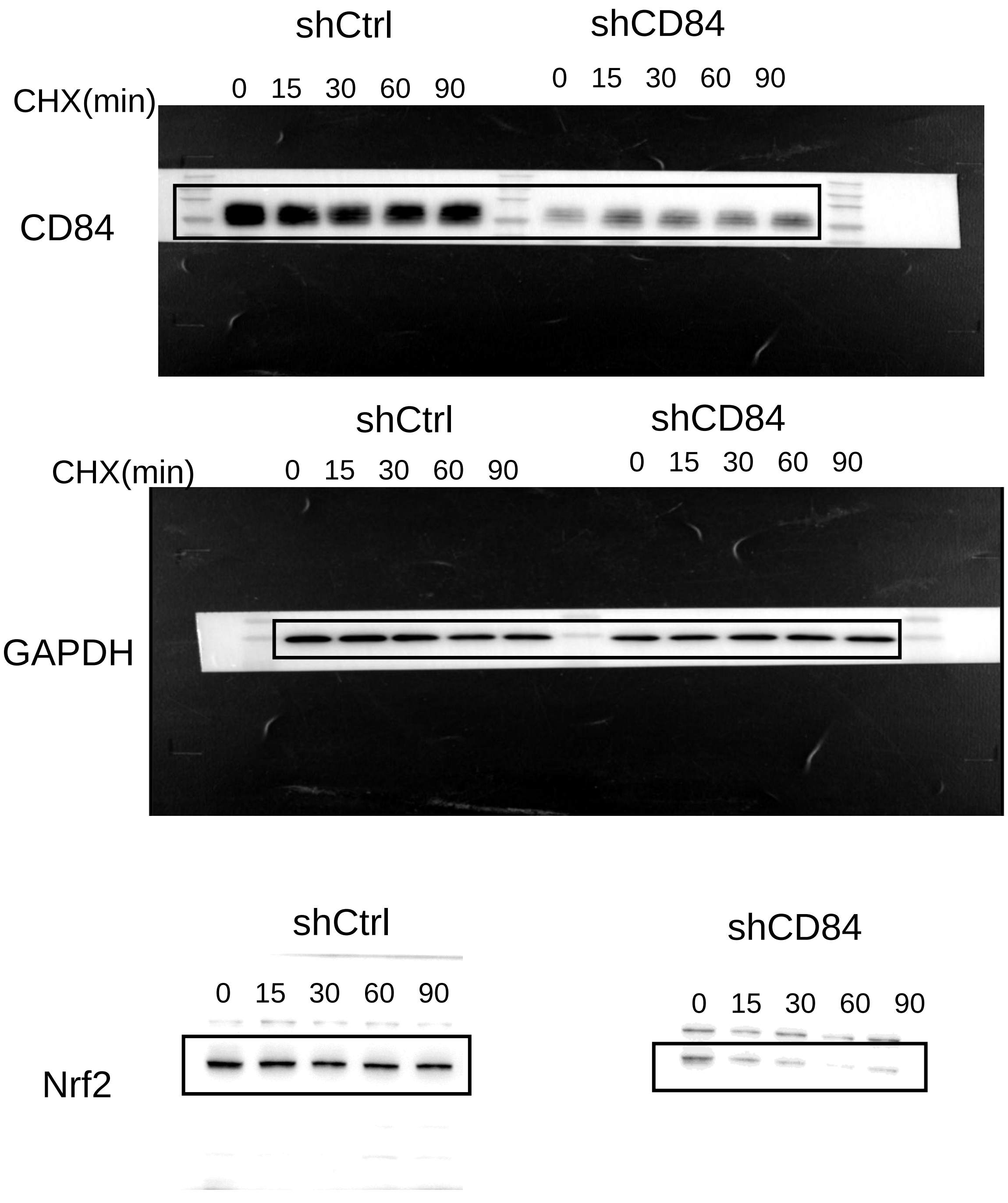
shCtrl
shCD84
CHX(min)
0 15 30 60 90
0 15 30 60 90
CD84
shCtrl
shCD84
CHX(min)
0 15 30 60 90
0 15 30 60 90
GAPDH
shCtrl
shCD84
0 15 30 60 90
0 15 30 60 90
Nrf2

Figure S6E

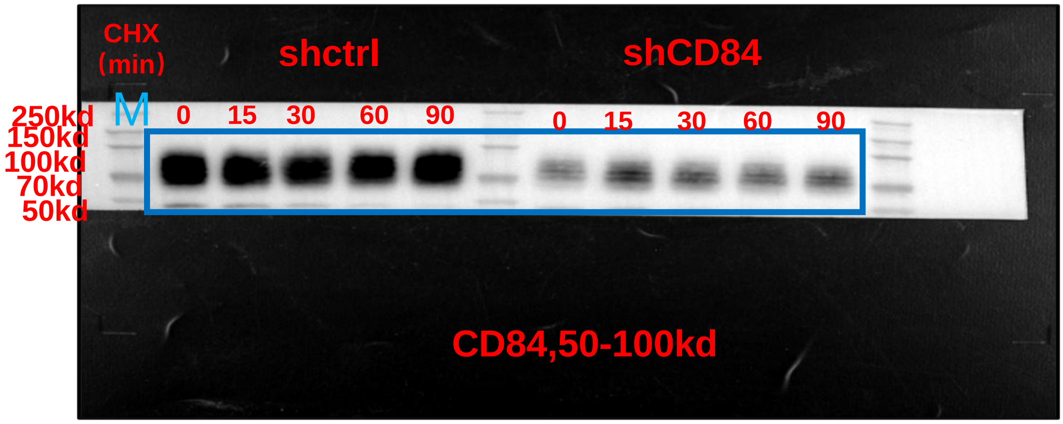
CHX
(min)
shctrl
shCD84
250kd
150kd
100kd
70kd
50kd
M
0
15
30
60
90
0
15
30
60
90
CD84,50-100kd

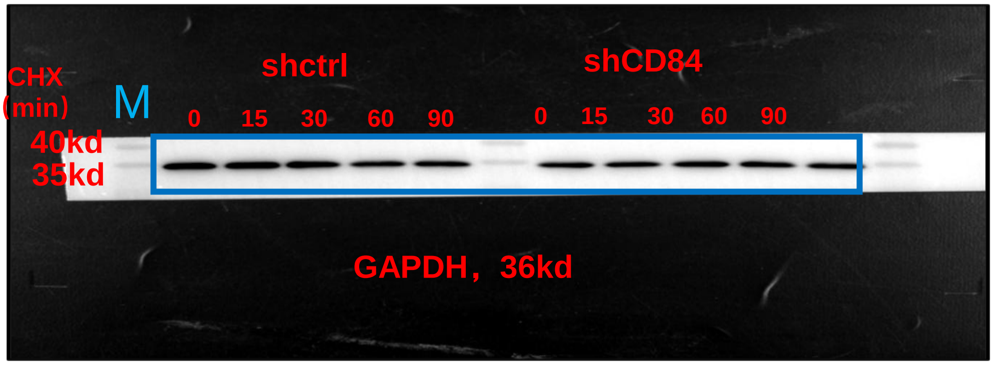
CHX
(min)
shctrl
shCD84
M
0
15
30
60
90
0
15
30
60
90
40kd
35kd
GAPDH，36kd

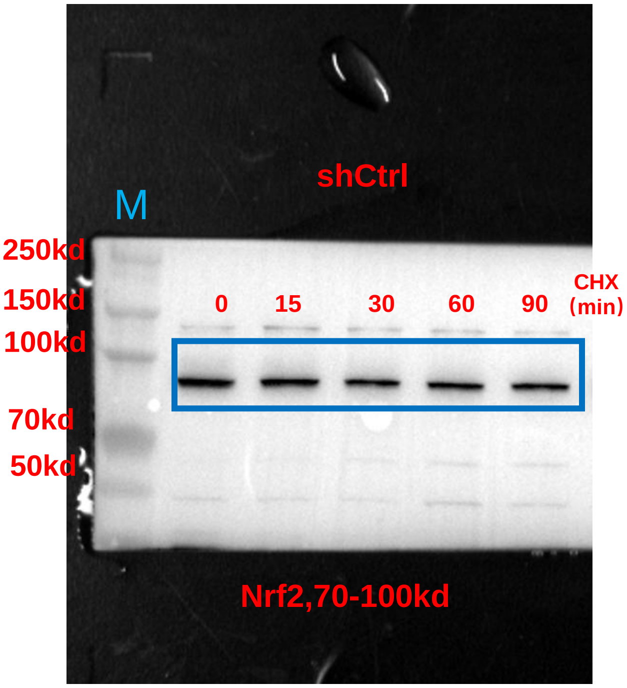
shCtrl
M
250kd
150kd
100kd
70kd
50kd
0
15
30
60
90
CHX
(min)
Nrf2,70-100kd

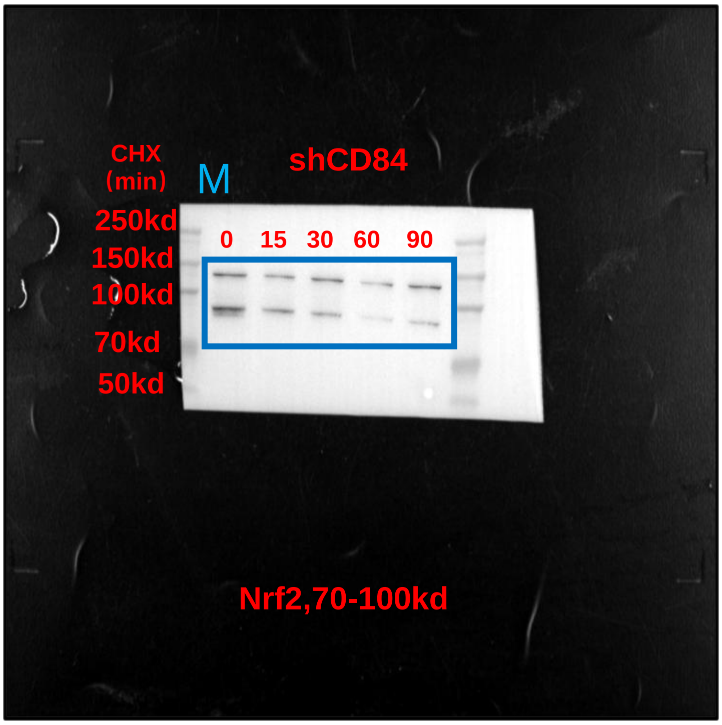
CHX
(min)
M
shCD84
250kd
150kd
100kd
70kd
50kd
0
15
30
60
90
Nrf2,70-100kd

Figure S6F

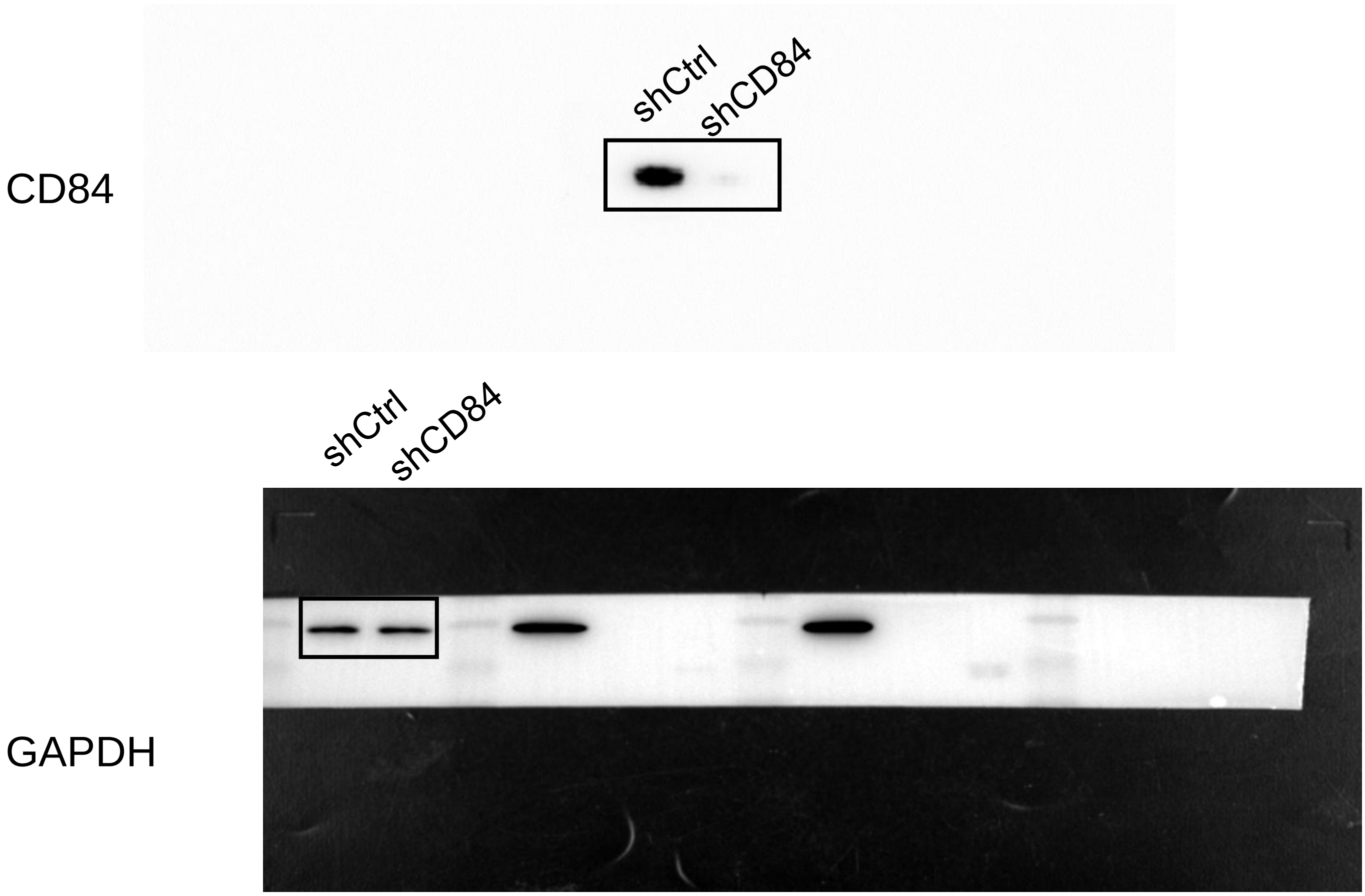

Figure S6F

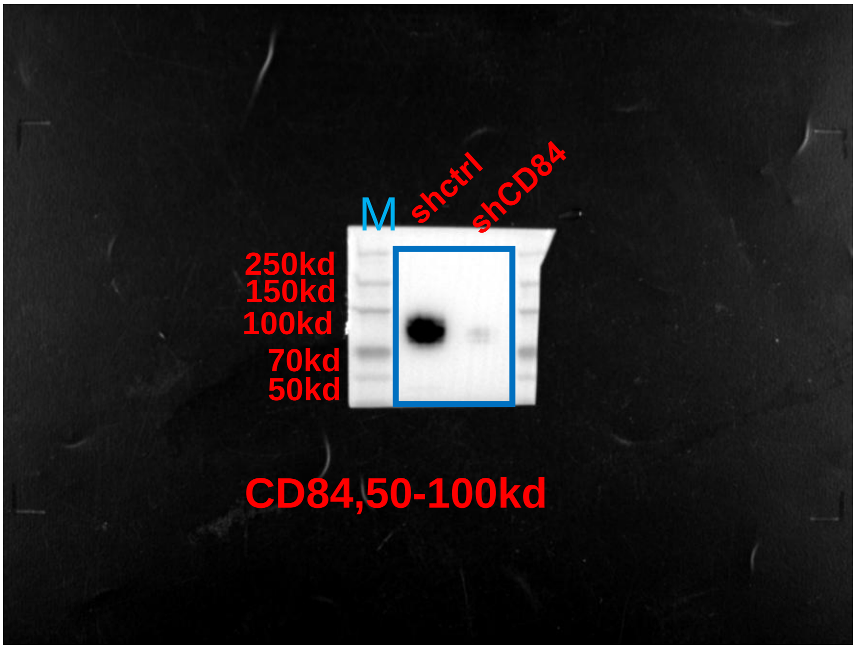
M
shctrl
shCD84
250kd
150kd
100kd
70kd
50kd
CD84,50-100kd

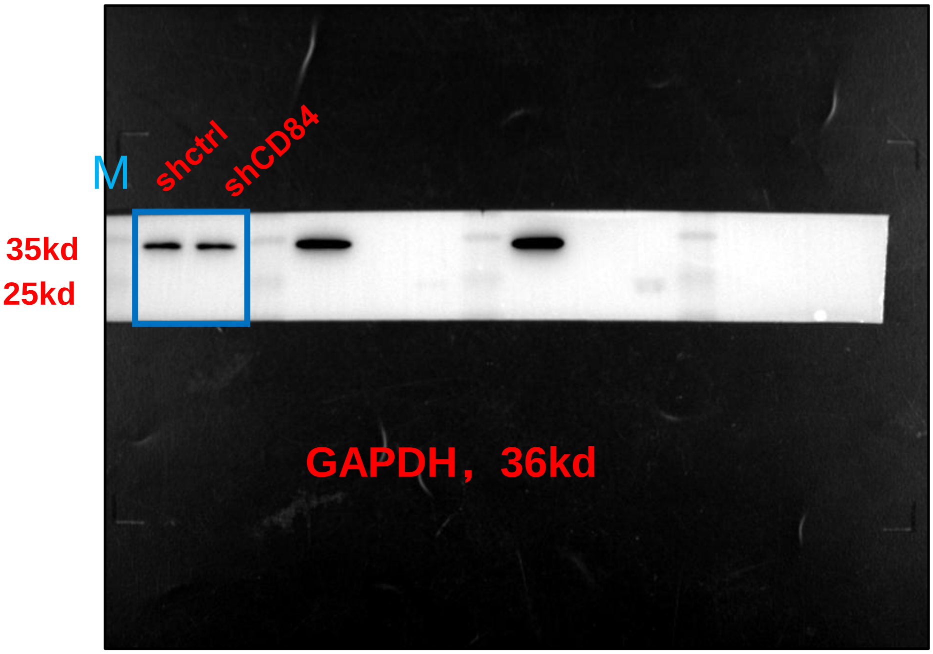
M
shctrl
shCD84
35kd
25kd
GAPDH，36kd

Figure S6G

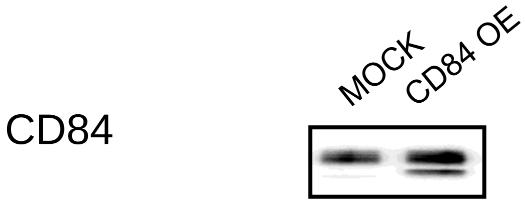

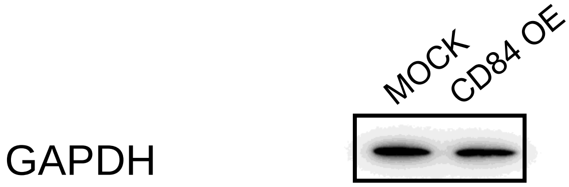

Figure S6G

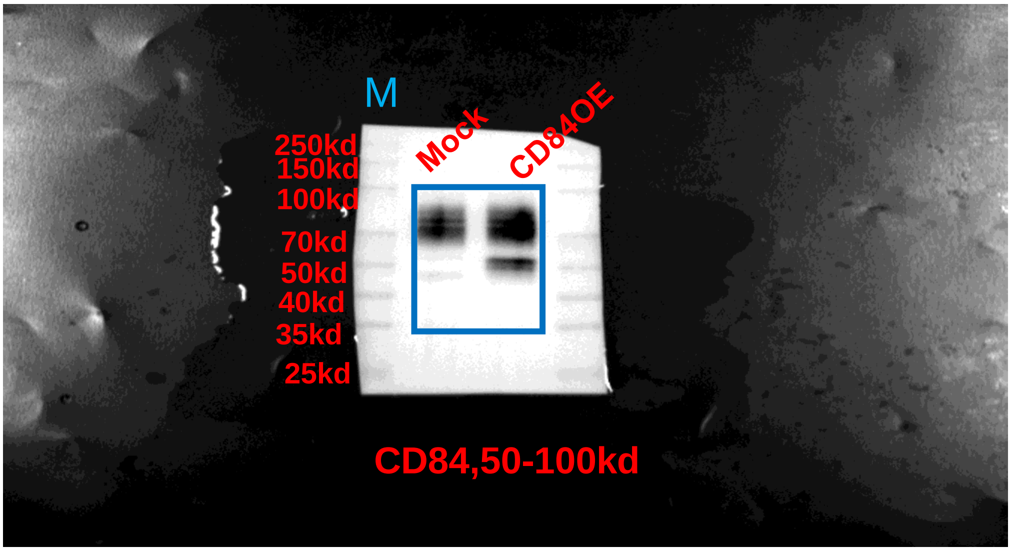
M
Mock
CD84OE
250kd
150kd
100kd
70kd
50kd
40kd
35kd
25kd
CD84,50-100kd

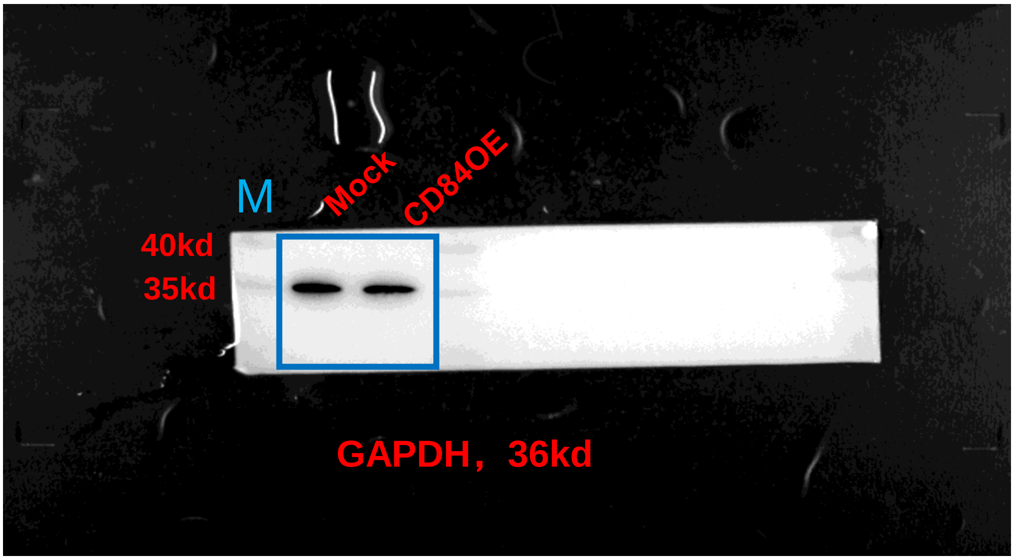
M
Mock
CD84OE
40kd
35kd
GAPDH，36kd
